# Mapping diphtheria-pertussis-tetanus vaccine coverage in Africa, 2000–2016: a spatial and temporal modelling study

**DOI:** 10.1016/S0140-6736(19)30226-0

**Published:** 2019-05-04

**Authors:** Jonathan F Mosser, William Gagne-Maynard, Puja C Rao, Aaron Osgood-Zimmerman, Nancy Fullman, Nicholas Graetz, Roy Burstein, Rachel L Updike, Patrick Y Liu, Sarah E Ray, Lucas Earl, Aniruddha Deshpande, Daniel C Casey, Laura Dwyer-Lindgren, Elizabeth A Cromwell, David M Pigott, Freya M Shearer, Heidi Jane Larson, Daniel J Weiss, Samir Bhatt, Peter W Gething, Christopher J L Murray, Stephen S Lim, Robert C Reiner, Simon I Hay

**Affiliations:** a Institute for Health Metrics and Evaluation, University of Washington, Seattle, WA, USA; b Department of Health Metrics Sciences, University of Washington, Seattle, WA, USA; c David Geffen School of Medicine, University of California Los Angeles, Los Angeles, CA, USA; d Big Data Institute, University of Oxford, Oxford, UK; e Department of Infectious Disease Epidemiology, London School of Hygiene & Tropical Medicine, London, UK; f Department of Infectious Disease Epidemiology, School of Public Health, Imperial College London, London, UK

## Abstract

**Background:**

Routine childhood vaccination is among the most cost-effective, successful public health interventions available. Amid substantial investments to expand vaccine delivery throughout Africa and strengthen administrative reporting systems, most countries still require robust measures of local routine vaccine coverage and changes in geographical inequalities over time.

**Methods:**

This analysis drew from 183 surveys done between 2000 and 2016, including data from 881 268 children in 49 African countries. We used a Bayesian geostatistical model calibrated to results from the Global Burden of Diseases, Injuries, and Risk Factors Study 2017, to produce annual estimates with high-spatial resolution (5 ×    5 km) of diphtheria-pertussis-tetanus (DPT) vaccine coverage and dropout for children aged 12–23 months in 52 African countries from 2000 to 2016.

**Findings:**

Estimated third-dose (DPT3) coverage increased in 72·3% (95% uncertainty interval [UI] 64·6–80·3) of second-level administrative units in Africa from 2000 to 2016, but substantial geographical inequalities in DPT coverage remained across and within African countries. In 2016, DPT3 coverage at the second administrative (ie, district) level varied by more than 25% in 29 of 52 countries, with only two (Morocco and Rwanda) of 52 countries meeting the Global Vaccine Action Plan target of 80% DPT3 coverage or higher in all second-level administrative units with high confidence (posterior probability ≥95%). Large areas of low DPT3 coverage (≤50%) were identified in the Sahel, Somalia, eastern Ethiopia, and in Angola. Low first-dose (DPT1) coverage (≤50%) and high relative dropout (≥30%) together drove low DPT3 coverage across the Sahel, Somalia, eastern Ethiopia, Guinea, and Angola.

**Interpretation:**

Despite substantial progress in Africa, marked national and subnational inequalities in DPT coverage persist throughout the continent. These results can help identify areas of low coverage and vaccine delivery system vulnerabilities and can ultimately support more precise targeting of resources to improve vaccine coverage and health outcomes for African children.

**Funding:**

Bill & Melinda Gates Foundation.

## Introduction

Routine childhood vaccination is among the most successful and cost-effective public health interventions,^1^, ^2^ substantially contributing to progress in child survival.^3^ Despite these gains, vaccine-preventable diseases remain a major cause of child mortality and morbidity, particularly in lower-income settings.^4^ Driven largely by the introduction of new vaccines and the global polio eradication initiative, development assistance for vaccination reached US$3·6 billion in 2014.^5^ Nonetheless, gaps in coverage persist for both new and established vaccines.^6^ To maximise the effect of these investments, a locally focused understanding of vaccine coverage patterns is crucial.

Because of its inclusion in the original Expanded Programme on Immunisation (EPI) and multidose schedule, diphtheria-pertussis-tetanus vaccine (DPT) coverage is a widely used measure of the performance of routine vaccine delivery systems. Dose-specific DPT coverage can be used to monitor initial engagement with the vaccine delivery system (first dose [DPT1] coverage), retention within the system (DPT1–3 dropout, or difference between DPT1 and third dose [DPT3] coverage), and completion of the initial routine infant vaccine series (DPT3 coverage).^7^ Amid calls for improved targeting of interventions in the precision public health era,^8^ robust measurement of local vaccine coverage is crucial. Estimates of vaccine coverage are routinely produced at the national level,^9^ but do not capture the local patterns of coverage required to provide optimal, child-focused vaccine delivery services.^10^ The importance of geographical parity in vaccine coverage is emphasised in the Global Vaccine Action Plan (GVAP), which sets dual targets of 90% national coverage and 80% coverage for all districts within countries by 2020, using DPT3 coverage as a marker.^11^ Efforts to track progress towards the GVAP district-level goals rely on subnational administrative data,^6^ but such data are not available for all countries and vary in quality.^12^ Local vaccine coverage estimates are also needed to precisely monitor progress towards the Sustainable Development Goals (SDGs), particularly those indicators focused on immunisation targets (ie, SDG indicator 3.b.1).^13^

**Research in context**

**Evidence before this study**

National level estimates of routine vaccine coverage are produced annually through the Global Burden of Diseases, Injuries, and Risk Factors Study (GBD) and the WHO–UNICEF Estimates of National Immunisation Coverage process. Demographic and Health Surveys, the UNICEF Multiple Indicator Cluster Surveys, and other survey series produce subnational estimates of vaccine coverage. However, these series are limited to the select countries and years in which surveys are done and are generally produced at coarse administrative levels because of limitations of sample size. Countries also routinely calculate both national and subnational administrative coverage by dividing the total number of doses given by the number of surviving infants. Administrative coverage, however, is prone to numerator errors (incorrect recording of the number of doses administered), denominator errors (incomplete knowledge of target population size), and numerator–denominator mismatch (ie, due to migration or care seeking across borders). Subnational administrative data is used to track progress towards district-level Global Vaccine Action Plan (GVAP) targets but is not available for all countries and varies greatly in quality. No universally comparable set of coverage estimates exists to benchmark progress towards this target.

Because geographical inequalities in vaccine coverage are likely to exist even at subdistrict levels, modelling approaches that use geolocated data might better define local patterns of coverage. With use of the search string “(“Geographic Mapping”[Mesh] OR “subnational”[All Fields] OR “geospatial”[All Fields] OR “geostatistical”[All Fields]) AND (“vaccination”[All Fields] OR “vaccines”[MeSH Terms] OR “vaccines”[All Fields] OR “vaccine”[All Fields] OR “vaccine coverage”[All Fields] OR “immunisation”[All Fields] OR “vaccination”[MeSH Terms] OR “vaccination”[All Fields] OR “immunization”[All Fields] OR “immunization”[MeSH Terms])”, we searched PubMed from inception to April 30, 2018, for English-language studies producing high-resolution subnational estimates of vaccine coverage. Several studies have used Bayesian model-based geostatistical methods to estimate local patterns of vaccine coverage, but they were limited to select countries, years, or both and included only a subset of available survey data.

**Added value of this study**

To our knowledge, this analysis provides the first annual estimates of diphtheria-pertussis-tetanus (DPT) coverage at a continental scale, with a resolution of 5 × 5 km, and at first and second administrative levels in children aged 12–23 months across 52 countries in Africa from 2000 to 2016. We sought to synthesise and geolocate all available subnationally-resolved survey data, incorporating data from 183 survey series encompassing 881 268 children in the final modelling process. With geostatistical methods, we produced estimates of vaccine coverage at the local and second administrative unit levels, whereas coverage estimation with traditional survey methods is often restricted to the first administrative level because of sample size limitations. To better define vaccine delivery system strengths and vulnerabilities, we produced local estimates of first-dose (DPT1) and third-dose (DPT3) coverage and DPT1–3 dropout. We used a uniform Bayesian model-based geostatistical modelling framework across all countries and calibrated results to national level estimates of vaccine coverage from GBD 2017 to enhance the comparability of estimates both within and between countries. These results allow tracking of progress towards subnational GVAP goals and provide a platform from which subnational administrative data can be triangulated with survey-based estimates.

**Implications of all the available evidence**

Together with existing subnational vaccine coverage estimates, these results show that national estimates alone are inadequate for monitoring trends in vaccine coverage. These subnational estimates can be triangulated with subnational administrative data, supporting efforts to strengthen administrative reporting systems. Despite widespread progress between 2000 and 2016, marked geographical inequalities in DPT coverage persist in Africa, both within and between countries. These enduring inequalities pose a substantial challenge to achieving GVAP targets and leave much of the continent at risk for preventable diseases and death. Our results allow local, national, and global decision makers to better understand local patterns of vaccine coverage and trends over time and to design more precise, high-impact interventions to increase vaccine coverage in Africa.

The use of subnational data for action is a key component of the Reaching Every District strategy,^14^, ^15^ which has been a key component of efforts to improve vaccine coverage across Africa since the early 2000s. Subnational vaccine coverages estimates have been produced at the first or second administrative levels from surveys or small-area-estimation analyses,^16^ but are available only for some countries and years. In many settings, administrative coverage^17^—calculated by dividing the number of doses administered by the estimated number of eligible children in an administrative unit^12^—is the only continuous source of subnational information available to track progress and guide vaccine policy.^14^ However, despite ongoing efforts to strengthen administrative data quality,^18^, ^19^, ^20^ problems persist. In 2016, 46 countries in Africa reported subnational administrative DPT3 coverage to WHO and UNICEF. In these, 38 countries reported at least one subnational administrative unit with more than 100% coverage, and more than half (24 of 46) reported coverage higher than 100% for more than a quarter of administrative units.^17^ These problems occur because subnational administrative coverage can be prone to numerator errors (incorrect number of doses delivered), denominator errors (inaccurate population estimates), and numerator–denominator mismatch (ie, due to mobility across subnational boundaries).^12^ Alternative subnational coverage estimates that incorporate non-administrative data sources—such as survey data—could be triangulated with administrative coverage to better understand vaccination patterns and further strengthen administrative data quality.

To date, no comparable subnational estimates of DPT coverage are available across countries and over time, which impedes the effective monitoring of key vaccination targets. Moreover, even subnational estimates conceal important local variation in vaccine coverage.^21^ Studies^22^, ^23^, ^24^, ^25^ published in the past few years have used Bayesian model-based geostatistical methods to map key health outcomes and determinants at high spatial resolution across Africa and over time. Other analyses^26^, ^27^ also have employed Bayesian geostatistical techniques to estimate vaccine coverage for selected antigens, countries, and years in Africa, but these analyses covered few countries, included only a subset of available survey data, or only estimated coverage for single years. To best inform vaccine policy and programme needs, local coverage estimates should be produced within a cohesive framework for all locations, support tracking changes in coverage over time, and capture patterns of both non-vaccination and under-vaccination. Our analysis aimed to provide the first comprehensive annual estimates of DPT1 and DPT3 coverage and DPT 1–3 dropout among children aged 12–23 months at local (5 × 5 km) and subnational (second administrative unit) spatial resolutions for 52 countries across Africa from 2000 to 2016.

## Methods

### Data collection

By use of the Global Health Data Exchange, a publicly-accessible catalogue of population health data, we identified population-based household surveys in Africa between 2000 and 2016 that included dose-specific information on DPT coverage (from vaccine cards or maternal recall in the absence of vaccine cards) and subnational geographical location for children aged 12–59 months (appendix). Vaccination status, age, and geographical location were extracted for 881 268 children in 183 surveys done in 49 countries across Africa. With use of individual-level survey data, we calculated dose-specific DPT coverage (the proportion of children with zero, one, two, or three or more doses) for four age cohorts (12–23, 24–35, 36–47, and 48–59 months) at the most precise geographical level possible. Data from children aged 24 months or older were reassigned to the year in which those children would have been 12–23 months old.

Latitude and longitude values were available for 42 384 survey clusters (so-called point data); for these, coverage was calculated at the cluster level and directly included in the geospatial model. For 45 112 survey clusters, no precise latitude and longitude was provided; for these data, dose-specific, and age-cohort-specific coverage was calculated for the most precise geographical unit available, taking survey design and sampling weights into account (appendix). The average coverage estimates for each surveyed geographical unit were then converted to point data by use of a previously described resampling method before inclusion in the geospatial model.^24^, ^25^, ^28^ Lastly, we assembled a collection of 26 spatial covariates (appendix), prioritising covariates previously linked to non-vaccination or under-vaccination.^29^

### Data analysis

This analysis combined survey data and a suite of spatial covariates in a two-step Bayesian model-based geostatistical framework^25^ to generate annual estimates of DPT1 and DPT3 coverage and DPT1–3 dropout for every 5 × 5 km area in Africa, from 2000 to 2016. This geostatistical model capitalises on the relationships between vaccine coverage and associated covariates while incorporating spatial and temporal correlations in the residuals to better predict coverage in places and years with scarce or no data available.

To model DPT3 coverage, we fitted separate models for five geographically contiguous regions in Africa, adapted from regions used in the Global Burden of Diseases, Injuries, and Risk Factors Study^4^ (GBD). This adaptation was necessary to exclude several countries in the GBD region of North Africa and Middle East that were outside of the geographical scope of this study (more details in the appendix). Each model was fit in two steps. In the first modelling step, stacked generalisation,^30^ several submodels (boosted regression trees, lasso regression, and generalised additive models) were used to predict vaccine coverage with the spatial covariates as predictors. This ensemble modelling step allows non-linear relationships and interactions between covariates to better predict coverage but does not explicitly account for spatial or temporal coverage patterns. In the second modelling step, a Bayesian geostatistical model was used to account for residual spatial and temporal correlation, improving local DPT coverage estimates. Vaccine coverage was modelled as binomial count data with a logit link function, using the results of stacked generalisation as predictors and including a correlated spatiotemporal error term, country-level random effects, and a nugget effect that represented irreducible observation-level error. To generate uncertainty for each model, 1000 samples (or draws) were obtained from the joint posterior distribution, where each draw represents one set of possible coverage values for each 5 × 5 km location across Africa and year in the study period. All models were fitted with integrated nested Laplace approximation in the R-INLA package in R, version 3.3.2.^31^, ^32^, ^33^

To ensure internal consistency—ie, that modelled dose-specific coverage estimates all sum to 100% in every place and time—we used a continuation ratio ordinal regression approach.^34^ The two-step modelling process previously described for DPT3 was repeated with identical input data and covariates for two conditional coverage quantities:

P(d=2|d≤2)

and

P(d=1|d≤1)

where *d* is the number of DPT doses received. Samples from the posterior of DPT3 coverage and these conditional coverage quantities were combined arithmetically to produce posterior distributions for DPT3 coverage (the proportion of children who received three or more doses of DPT), DPT1 coverage (one or more doses), absolute DPT1–3 dropout (DPT3–DPT1), and relative DPT1–3 dropout ([DPT3–DPT1]/DPT1). Compared with other options for ordinal regression, such as the proportional odds model, the continuation ratio model allows the association between covariates and the odds of vaccination to vary by the number of doses received. Covariate–coverage associations varied between the conditional coverage geostatistical models (appendix), supporting the use of the continuation ratio model. Estimates of DPT3, DPT1, and DPT1–3 dropout produced by this model agreed closely with values calculated directly from survey data in an out-of-sample validation framework (appendix).

We calibrated geospatial estimates in logit space to DPT coverage at the national level estimated by GBD (appendix).^35^ This process preserves relative spatial patterns while ensuring that the population-weighted average of the calibrated geospatial estimates for a given country and year equals the corresponding GBD estimate. Calibrated draws for each indicator were summarised as mean estimates, uncertainty intervals (UIs), and probabilities of achieving coverage targets. Second-level administrative estimates were calculated as population-weighted means by use of administrative boundaries from the Global Administrative Unit Layers (GAUL) database (GAUL boundary definitions were also used to define second-level administrative units in this analysis).^36^ We assessed absolute subnational inequalities by use of the range of second-level administrative unit mean estimates within a country. Relative subnational coverage variation was assessed by use of ratios of estimated second-level administrative coverage to the national average.

Both in-sample and out-of-sample model validation was done by use of spatiotemporal five-fold cross-validation, with spatial stratification at the second administrative level. Out-of-sample predictive metrics for DPT3 coverage indicated good model fit, including mean error (0·1%), mean absolute error (6·7%), root-mean-square error (0·097), and 95% coverage of predictive intervals (92·5%), with similar findings for other coverage indicators. By comparison with alternative models without spatiotemporal effects, with raw covariates or no spatial covariates, or both, the combination of ensemble modelling and spatiotemporal effects generally improved model performance (appendix). In three countries (South Africa, Libya, and Cape Verde), no surveys that included cluster-level information on both DPT vaccination coverage and subnational geographical information were identified. In South Africa, for instance, Demographic and Health Surveys were done in 2003 and 2016, but cluster-level subnational geographical information was not available at the time of this analysis. In these countries, the model produced coverage estimates by use of modelled relationships between DPT coverage and covariates (from other countries within the modelling region) and national-level coverage estimates from the GBD study.

Additional results and details on data preparation, modelling, estimation, validation, and comparison with existing estimates can be found in the appendix and the online visualisation tool.

### Role of the funding source

The funders of the study had no role in study design, data collection, data analysis, data interpretation, or writing of the report. The corresponding authors had full access to all the data in the study and had final responsibility for the decision to submit for publication.

## Results

### DPT3 coverage

**Figure 1:**
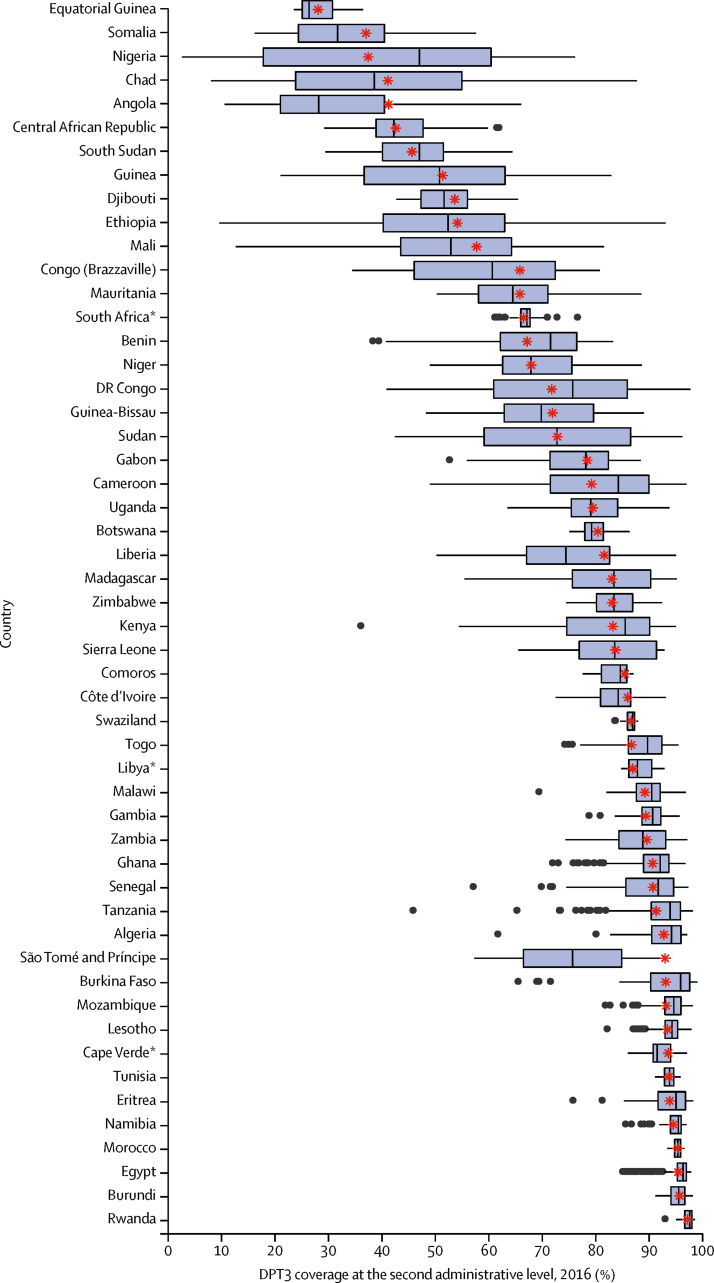
Distribution of diphtheria-pertussis-tetanus third-dose (DPT3) vaccine coverage at the second administrative level for 52 countries in Africa, 2016 Each box plot displays the distribution of estimated DPT3 coverage among second administrative units in 2016 for a single country. National mean DPT3 coverage estimates from GBD 2017 are shown as red asterisks. *No data were available for these countries.

For most countries in Africa, national coverage estimates inadequately conveyed subnational variation in DPT3 coverage (figure 1). In 2016, mean estimated DPT3 coverage at the second administrative level varied by more than 25% in 29 of 52 countries, and eight of 52 countries contained at least one second-level administrative unit with estimated DPT3 coverage that was less than half the national average (figure 1). Notably, the countries with the largest subnational relative inequalities in coverage were also among those with the lowest national level coverage in Africa, including Nigeria (where 2016 DPT3 coverage for the second administrative level ranged from 7% to 203% of the national average), Chad (20–213%), Ethiopia (18–172%), and Angola (26%–160%). However, large relative inequalities in second administrative units also were noted in higher-coverage countries, including Kenya (44–114% of the national average) and Tanzania (50–107%). Several countries displayed large absolute gaps in DPT3 coverage at the second administrative level. The largest absolute subnational variation was found in Ethiopia, where DPT3 coverage estimates at the second administrative level in 2016 ranged from 93·1% (95% UI 89·1–95·9) in Addis Ababa to 9·7% (5·7–15·1) in Fantena Rasu. Similarly, we observed a large variation in Chad (from 87·8% [95% UI 78·2–94·4] in Beboro to 8·1% [1·9–20·5] in Deredia) and Nigeria (from 76·2% [61·1–87·5] in Esan North-East to 2·7% [1·0–5·5] in Wurno).

**Figure 2:**
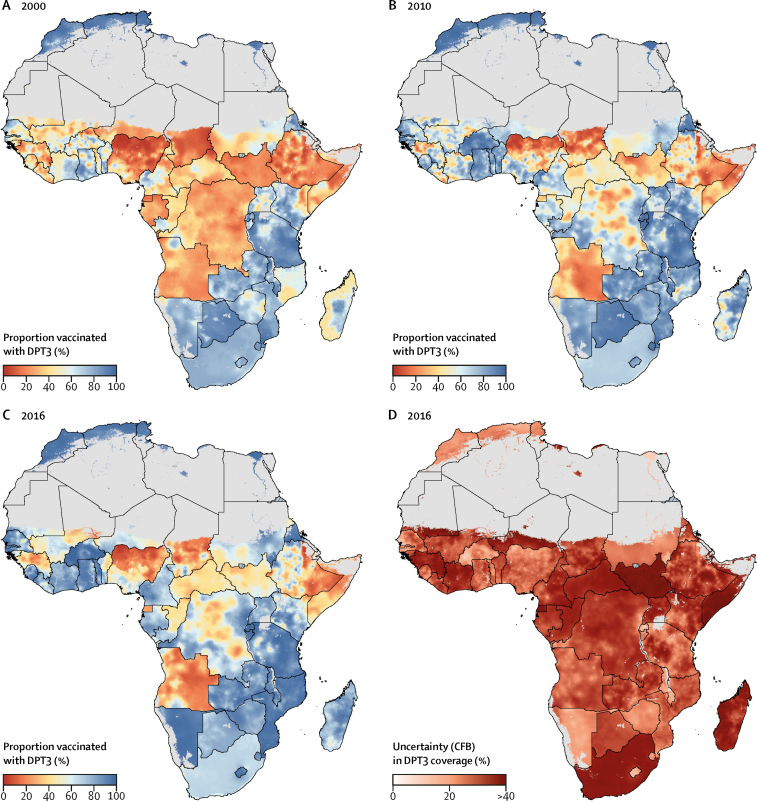
Estimated diphtheria-pertussis-tetanus third-dose (DPT3) vaccine coverage in Africa, 2000–16 (A–C) DPT3 coverage among children aged 12–23 months with a 5 × 5 km resolution in 2000, 2010, and 2016. (D) Model uncertainty in 2016; model uncertainty is displayed by use of the Coffey-Feingold-Bromberg metric (CFB), a measure of uncertainty that is comparable regardless of mean coverage and scales from 0% (no uncertainty) to 100% (highest possible uncertainty for a given mean). Results are masked in grey in areas where total population density was less than ten individuals per 1 × 1 km pixel in 2015 per WorldPop^37^ estimates, or where land cover was classified as “barren or sparsely vegetated” on the basis of MODIS^38^ satellite data in 2013. No data were available for Cape Verde, Libya, and South Africa.

**Figure 3:**
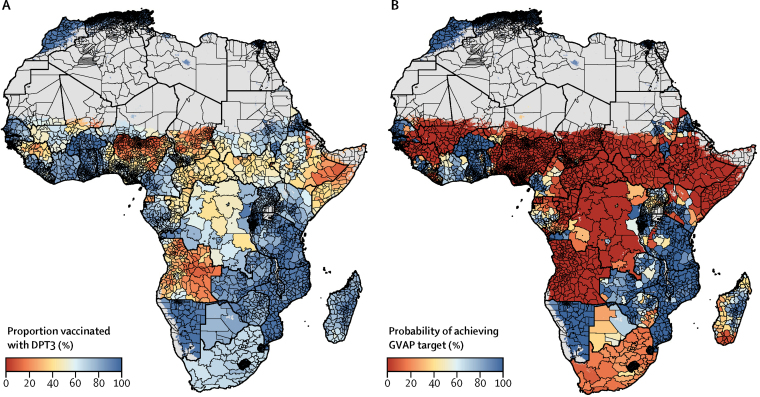
Estimated diphtheria-pertussis-tetanus third-dose (DPT3) vaccine coverage in Africa by administrative district and probabilities of achieving Global Vaccine Action Plan (GVAP) target coverage in 2016 (A) DPT3 coverage among children aged 12–23 months at the second administrative level. (B) Probability of second-level administrative unit achieving the GVAP target of 80% DPT3 coverage or higher in 2016. Results are masked in grey in areas where total population density was less than ten individuals per 1 × 1 km pixel in 2015 per WorldPop^37^ estimates, or where land cover was classified as “barren or sparsely vegetated” on the basis of MODIS^38^ satellite data in 2013. No data were available for Cape Verde, Libya, and South Africa.

At the local level, patterns of DPT3 coverage varied markedly across Africa in 2000–16 (figure 2). In 2016, DPT3 coverage was lowest across the Sahel region (a region spanning parts of Senegal, Mauritania, Mali, Burkina Faso, Niger, Nigeria, Chad, and Sudan), Somalia, eastern Ethiopia, South Sudan, Guinea, Central African Republic, Equatorial Guinea, parts of DR Congo, and most of Angola (figure 2C). Of the ten second-level administrative units with the lowest estimated DPT3 coverage in Africa in 2016, all were in Sokoto State in north-western Nigeria (figure 3A), and all had mean DPT3 coverage estimates lower than 5%. By contrast, DPT3 coverage estimates of second administrative divisions were highest in parts of Burkina Faso and Rwanda, with large areas of northern Africa, the African Great Lakes region, Zambia, Mozambique, Namibia, and Eritrea reaching DPT3 coverage of 90% or higher (figure 3A).

**Figure 4:**
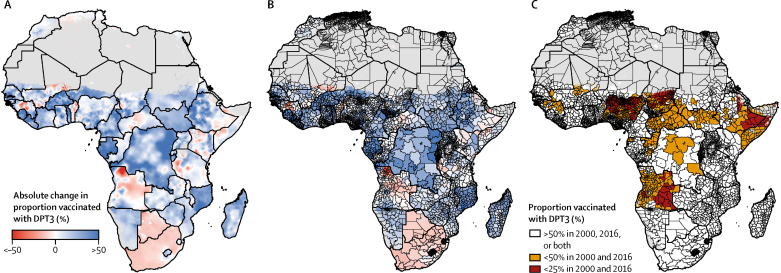
Estimated changes in diphtheria-pertussis-tetanus third-dose (DPT3) vaccine coverage in Africa, 2000–16 Mean estimated changes in DPT3 coverage among children aged 12–23 months between 2000 and 2016 with a 5 × 5 km resolution (A) and at the second administrative level (B). Colours represent estimated absolute change (%), with positive changes (increased coverage) represented in blue and negative changes (decreased coverage) in red. Increases and decreases of 50% or higher are represented by dark blue (increases) and dark red (decreases). (C) Areas of low DPT3 coverage over time; second administrative units with coverage lower than 25% in both 2000 and 2016 are represented in red, whereas units with coverage ranging from 25% to lower than 50% in both 2000 and 2016 are represented in orange. Results are masked in grey in areas where total population density was less than ten individuals per 1 × 1 km pixel in 2015 per WorldPop^37^ estimates, or where land cover was classified as “barren or sparsely vegetated” on the basis of MODIS^38^ satellite data in 2013. No data were available for Cape Verde, Libya, and South Africa.

Although continental and national geographical inequalities in DPT3 coverage persisted in 2016, these estimates also revealed substantial gains in DPT3 coverage since 2000. DPT3 coverage increased throughout much of Africa between 2000 and 2016 (figure 2, figure 4), though unevenly. From 2000 to 2016, mean estimated DPT3 coverage increased in 72·3% (95% UI 64·6–80·3) and decreased in 27·7% (19·7–35·4) of second-level administrative units in Africa, showing an overall trend of progress across the continent (appendix). Coverage decreases were estimated with high certainty (posterior probability >95%) in second-level administrative units in Angola, Benin, Botswana, Egypt, Ethiopia, Guinea, Kenya, Malawi, Mali, São Tomé and Príncipe, and Tanzania. Despite the overall trend towards progress, some subnational areas started and ended the study period with low DPT3 coverage. Second-level administrative units in Chad, Equatorial Guinea, Ethiopia, Somalia, Angola, and throughout most of northern Nigeria had estimated DPT3 coverage lower than 25% in both 2000 and 2016. Additional areas of Guinea, Benin, Mali, Central African Republic, Niger, Congo, DR Congo, Sudan, and South Sudan both started and ended the study period with estimated DPT3 coverage below 50% (figure 4C).

Subnational trends in DPT3 coverage have been particularly uneven in several countries with a large population and low DPT3 coverage (figure 4). In Nigeria, for instance, coverage gains were largely isolated to the south, whereas stagnating or decreasing DPT3 coverage was found throughout most of the northeast and northwest. In 2000, many areas of western Ethiopia had some of the lowest DPT3 coverage estimates in Africa, but recorded some of the continent's largest increases in coverage by 2016 (eg, in Asosa, coverage rose from 20·7% [95% UI 14·0–29·0] in 2000 to 84·7% [79·4–89·3] in 2016). At the same time, much of eastern Ethiopia achieved minimal gains. In Kilbet Rasu, for example, DPT3 coverage was low in 2000 (23·2% [95% UI 14·9–33·7]) and remained low in 2016 (12·9% [8·7–18·9]).

### DPT1 coverage

As with DPT3 coverage, DPT1 coverage increased throughout most of Africa from 2000 to 2016 (appendix), with estimated DPT1 coverage increasing in 69·5% (95% UI 61·0–76·8) of second-level administrative units and decreasing in 30·5% (23·2–39·0). Estimated changes in DPT1 coverage at the second administrative level ranged from a 67·9 percentage point increase to a 53·3 percentage point decrease, with the largest estimated gains found in parts of Sierra Leone, southern Chad, and western Ethiopia (appendix).

**Figure 5:**
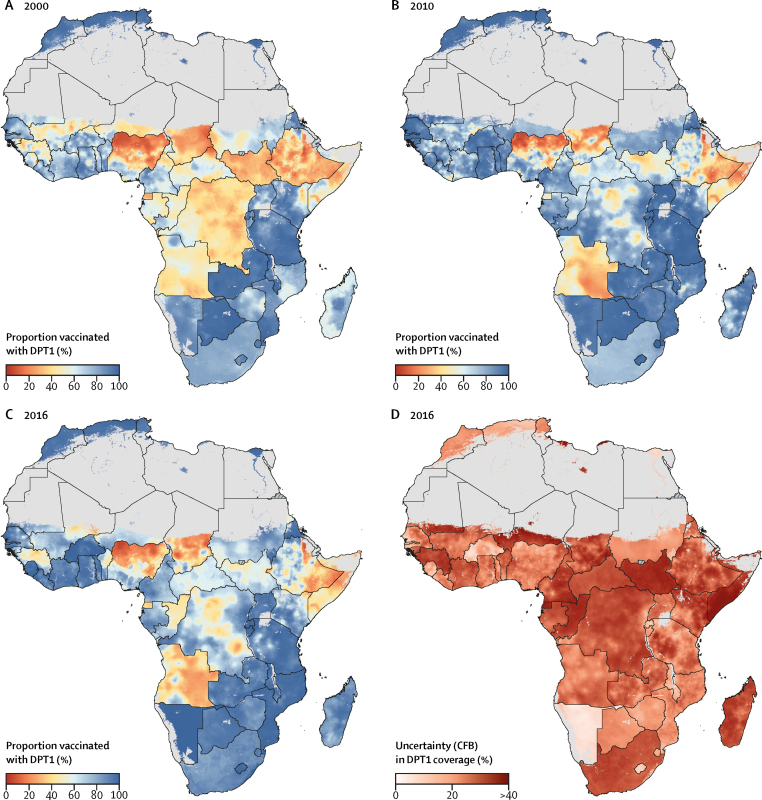
Estimated diphtheria-pertussis-tetanus first-dose (DPT1) vaccine coverage in Africa, 2000–16 (A–C) DPT1 coverage among children aged 12–23 months at the 5 × 5 km resolution in 2000, 2010, and 2016. (D) Model uncertainty in 2016; model uncertainty is displayed by use of the Coffey-Feingold-Bromberg metric (CFB), a measure of uncertainty that is comparable regardless of mean coverage and scales from 0% (no uncertainty) to 100% (highest possible uncertainty for a given mean). Results are masked in grey in areas where total population density was less than ten individuals per 1 × 1 km pixel in 2015 per WorldPop^37^ estimates, or where land cover was classified as “barren or sparsely vegetated” on the basis of MODIS^38^ satellite data in 2013. No data were available for Cape Verde, Libya, and South Africa.

In 2016, estimated DPT1 coverage exceeded 90% in much of Namibia, Zambia, Eritrea, Mozambique, and Lesotho and across northern Africa, along with large parts of the African Great Lakes region and western Africa (figure 5). However, in some areas—notably much of Angola, northern Nigeria, Chad, Somalia, Equatorial Guinea, Congo, eastern Ethiopia, as well as parts of South Sudan, Guinea, and DR Congo—estimated DPT1 coverage was lower than 50% in both 2000 and 2016 (figure 5, appendix).

Subnational disparities in initial vaccine delivery system engagement by the population, as measured by DPT1 coverage, can impede geographical parity in DPT3 coverage. In 2016, the largest absolute subnational disparity in DPT1 coverage at the second administrative level occurred in Nigeria, ranging from 5·0% (95% UI 3·0–8·4) in Wurno, Sokoto, to 90·8% (86·4–94·5) in Surulere, Lagos. Nigeria, Chad, Ethiopia, Mali, Angola, Kenya, and DR Congo all had mean DPT1 coverage disparities at the second administrative level of 50% or greater in 2016. Notably, DPT1 coverage was highly likely (posterior probability >95%) to be lower than 80% in 2016 across a continuous band stretching from northern Benin and northern Nigeria to Ethiopia and Somalia; in most of Angola; in Equatorial Guinea and contiguous areas of Central African Republic, Congo, and DR Congo; and in parts of Guinea and Mali (appendix).

### DPT1–3 dropout

**Figure 6:**
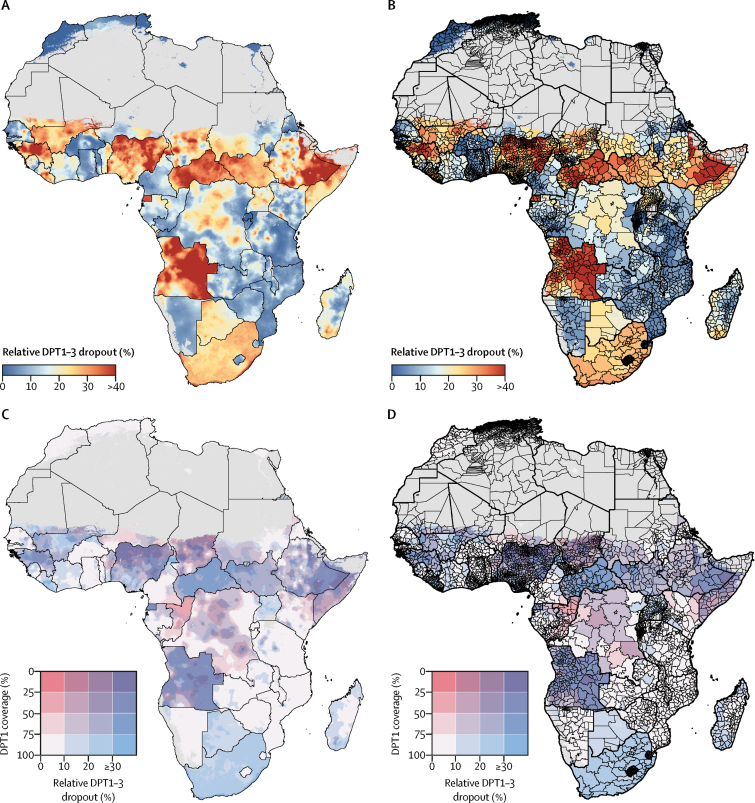
Estimated relative diphtheria-pertussis-tetanus (DPT) vaccine dropout (first dose minus third dose; DPT1–3) in Africa, 2016 DPT1–3 relative dropout with a 5 × 5 km resolution (A) and at the second administrative unit level (B). (C, D) Bivariate maps of DPT1 coverage and DPT1–3 relative dropout; each grid square represents a range of DPT1 coverage (vertical axis, white to red) and DPT1–3 dropout (horizontal axis, white to blue) for each modelled 5 × 5 km area (C) and second-level administrative unit (D) in Africa. Results are masked in grey in areas where total population density was less than ten individuals per 1 × 1 km pixel in 2015 per WorldPop^37^ estimates, or where land cover was classified as “barren or sparsely vegetated” on the basis of MODIS^38^ satellite data in 2013. No data were available for Cape Verde, Libya, and South Africa.

Areas with high DPT1–3 dropout were mostly similar to patterns of low DPT1 and DPT3 coverage. For instance, across parts of Nigeria, Angola, Chad, Mali, Guinea, Liberia, Equatorial Guinea, Central African Republic, South Africa, Somalia, and Ethiopia, estimated relative dropout exceeded 25% (figure 6). By contrast, northern Africa, Namibia, eastern Africa (including Rwanda, Burundi, parts of Kenya, Tanzania, Mozambique, Zimbabwe, and Zambia), and western Africa (namely Burkina Faso, Ghana, Togo, and western Senegal) had estimated relative dropout of 10% or lower in 2016 (figure 6A, 6B). In these places, more than 90% of children who received a first dose of DPT went on to finish the three-dose series.

From 2000 to 2016, DPT1–3 dropout decreased throughout much of Africa (appendix). 71·6% (95% UI 65·0–79·7) of second-level administrative units had estimated declines in relative dropout, suggesting broad improvements in vaccine system retention across the continent. At the same time, relative dropout rose in some areas. The largest increases in relative dropout were estimated in the Gao Region of northeastern Mali and the Uíge province in northern Angola. Angola also had the widest disparities in relative DPT1–3 dropout across second-level administrative units in 2016, ranging from 8·8% (95% UI 3·6–17·4) in Porto Amboim to 61·9% (37·4–82·5) in Cacolo (appendix).

Low DPT3 coverage could result from poor initial engagement with the routine infant immunisation system (low DPT1 coverage), poor retention within the system (high DPT1–3 dropout), or both. To illustrate these relationships, figures 6C, 6D show both DPT1 coverage and relative DPT1–3 dropout in a single plot. In some areas, such as northern Cameroon or parts of South Africa and Botswana, initial engagement with the vaccine system was strong (estimated DPT1 coverage >80%), but relative dropout lower than 20% resulted in substantially diminished DPT3 coverage. In other areas, such as parts of Congo, DPT1 coverage was 60% or less, but more than 90% of children who received a first dose of DPT completed the three-dose series. In some parts of northern Nigeria, Somalia, eastern Ethiopia, Chad, Equatorial Guinea, Guinea, and Angola, low DPT3 coverage resulted from both low DPT1 coverage (50% or lower DPT1 coverage) and high dropout (30% or higher DPT1–3 dropout), resulting in some of the lowest DPT3 coverage estimates in Africa.

### Progress toward GVAP targets

Of the 52 countries in this analysis, two (Morocco and Rwanda) were estimated to have already met the GVAP threshold of 80% DPT3 coverage or higher in all second-level administrative units in 2016 with high certainty (posterior probability >95%), an increase from no countries in 2000. For some countries, DPT3 coverage was uniformly low. For instance, several countries were very likely not to have any second-level administrative units with 80% DPT3 coverage or higher in 2016, such as Equatorial Guinea (posterior probability=98·2%) and Angola (95·2%). In Angola, no second-level administrative units had more than 2% posterior probability of achieving 80% DPT3 coverage in 2016 (figure 3B). Additionally, in Nigeria, only 0·7% (95% UI 0·0–1·8) of second-level administrative units were estimated to have met the 80% GVAP threshold by 2016.

In other countries, the probability of meeting the GVAP threshold varied substantially by second administrative level (figure 3B). In DR Congo, for instance, 42·0% (95% UI 34·0–52·0) of second-level administrative units were estimated to have achieved 80% DPT3 coverage or higher in 2016. However, in Ethiopia, only Addis Ababa and Asosa were highly certain to have achieved 80% DPT3 coverage or higher in 2016, whereas most (86·7%) second-level administrative units were highly unlikely to have met this threshold.

## Discussion

To our knowledge, this analysis represents the first continent-wide, high-resolution estimation of DPT coverage over space and time in 52 countries in Africa, a crucial input for strengthening local vaccine programmes and investments. In much of Africa, both DPT1 and DPT3 coverage improved and DPT1–3 dropout decreased during 2000–16. Notably, 2000–16 is a period of heightened financial and political commitment to scaling up vaccine delivery and the creation of Gavi, the Vaccine Alliance.^5^ However, progress was far from universal, leaving wide areas in Africa with low DPT1 and DPT3 coverage, high dropout between first and third doses of DPT, and vast subnational inequalities in coverage and dropout in many countries. The subnational inequalities observed in this study show that national estimates alone are inadequate to monitor vaccine coverage. National estimates can mask subnational pockets of low coverage across Africa, leaving children in those areas at risk for preventable diseases and death. These results offer a tool for decision makers to better understand local patterns of vaccine coverage and to identify where strengthening vaccine delivery systems might have the greatest effect.

By estimating local patterns of DPT1 and DPT3 coverage and DPT1–3 dropout, these results provide a window into subnational vaccine delivery system performance. For instance, Angola is in the process of transitioning out of Gavi support, but continues to receive Gavi support through a country-specific post-transition plan.^39^ These estimates reveal uneven progress over time, persistently low DPT1 and DPT3 coverage, and high DPT1–3 dropout across much of the country. Angola's transition period has been marked by vaccine stockouts and supply shortages in part because of large birth cohorts in the country and vaccine cofinancing challenges,^40^ and these estimates highlight the risk that Angola might exit Gavi support with large areas of persistently low DPT coverage. In Nigeria, which was facing imminent transition out of Gavi support until an exceptional extension to provide support through 2028 was granted,^41^ these estimates identified persistent striking subnational coverage inequalities. The successful transition from Gavi support in Nigeria will require reliable subnational coverage estimates, to both prioritise resources at the federal level and design targeted state-level strategies to improve coverage.^42^ By contrast, the large coverage gains estimated in western Ethiopia might hold important lessons for other areas with persistently low DPT coverage, and the contribution of specific strategies (such as Ethiopia's Health Extension Programme)^43^ to this progress is worthy of further investigation. Nonetheless, sizeable gaps persist between urban areas, such as Addis Ababa, and the rest of the country, and gains have been much harder to achieve in pastoralist areas in the northeast or the eastern region than in more urbanised areas. By tracking progress and geographical distribution over time, local vaccine coverage estimates could help to identify locations for further study, to assess whether successful efforts to strengthen health systems or enhance vaccine confidence in some locations could be more broadly applied.

Many countries in sub-Saharan Africa face substantial challenges in achieving the GVAP national level target of 90% coverage,^44^ and these challenges extend also to geographical parity targets. Although many countries have made substantial progress towards the GVAP target of 80% DPT3 coverage or higher in all of their second-level administrative units, only two of 52 countries were likely to have met this target by 2016. Attempts to monitor progress towards the district-level GVAP target rely on subnational administrative data,^6^ but this approach is considerably limited by data availability and reliability, which vary from country to country. By producing comparable estimates of coverage in a probabilistic framework, this analysis provides a new tool to monitor subnational progress towards the GVAP goals.

In some countries, the probability of meeting the GVAP threshold varied substantially by second administrative level (figure 3B). Focused improvements in lower-performing areas within these countries will be required to ensure that subnational GVAP targets are reached by 2020. Understanding patterns of low DPT1 coverage and DPT1–3 dropout at a subnational scale might help countries reach these targets. Our analysis showed that, in 2016, estimated DPT1 coverage exceeded 90% in numerous regions of the continent (figure 5), suggesting that initial infant engagement with the routine EPI schedule is robust in such regions. By contrast, in some regions, estimated DPT1 coverage was lower than 50% in both 2000 and 2016 (figure 5, appendix), suggesting that, in these regions, more than half of all children aged 12–23 months have never received a dose of DPT. Additionally, DPT1 coverage was highly likely to be lower than 80% in 2016 in large areas of the continent. In such areas, our results suggest that initial engagement with the routine infant schedule is a substantial barrier to attaining subnational GVAP goals for DPT3 coverage. Therefore, areas with low DPT1 coverage and low DPT1–3 dropout might benefit most from interventions targeting initial engagement. Where these results estimate high DPT1 coverage and low DPT1–3 dropout, enhancing retention within the vaccine delivery system would be important. Where estimated DPT1–3 dropout is high and DPT1 coverage is low, both approaches might be necessary to achieve DPT3 coverage targets (figure 6). By identifying areas of particularly low DPT1–3 dropout and trends over time, these results could improve the evidence base from which both national and local vaccine stakeholders can assess progress and allocate resources, a key pillar of the Reaching Every District approach.^14^, ^15^

Our study shows that Bayesian model-based geostatistical estimates can be a useful tool to analyse subnational vaccine coverage patterns. Changing administrative boundaries over time can make interpretation of trends in vaccine coverage from administrative data, surveys, and small area estimates challenging. By incorporating data at the finest geographical scale possible, estimating coverage at the 5 × 5 km level, and then calculating administrative-level coverage, this method is robust to such boundary changes, allowing for the analysis of changes in coverage over time. Compared with previous efforts to estimate local patterns of vaccine coverage,^26^, ^27^ this study draws from a much larger database of household surveys and maximises predictive accuracy through its Bayesian model-based geostatistical framework. These previous efforts mostly focused on estimating location-specific vaccine coverage but did not account for trends over time; by contrast, our study provides both geographical and temporal dimensions within a cohesive estimation approach. This study represents a novel application of a continuation-ratio ordinal regression framework to Bayesian geostatistical models, allowing the estimation of internally consistent and dose-specific coverage levels and dropout. Finally, this study was the first to estimate local patterns of vaccine coverage over time at a continental scale. By applying a uniform modelling framework across all estimated countries and calibrating to national level GBD estimates, this study enhanced the comparability of estimates within and between countries.

Both the data used in the analysis and these methods are subject to several limitations. First, data were survey-derived and included information obtained from vaccine cards and maternal recall. This approach improves data availability among populations where vaccine cards are not available but might introduce recall bias and might not explicitly account for doses administered during campaigns. Second, data were included at administrative levels if precise coordinates were not available, which might have resulted in a flattening of spatial variation in local estimates. However, excluding these data risked obscuring or skewing important trends in coverage where geolocated data were not available. Improved collection and availability of georeferenced location data will improve estimates of local disparities in vaccine coverage. Third, following the GBD approach, we used data from children aged 12–59 months alive at the time of survey to retrospectively estimate past coverage among children aged 12–23 months, to better capture historical trends and use coverage observations from geographical locations where only children aged 24 months or older were sampled. This method assumes negligible catch-up vaccination, does not incorporate uncertainty due to poor card retention rates in older age cohorts, and cannot account for potential effects of migration or differential mortality by vaccine status. Fourth, our ensemble modelling approach improved the ability of the model to predict vaccine coverage by allowing for complex and non-linear interactions between covariates and coverage. However, this predictive model was not designed to infer which covariates drive vaccine coverage. Further methods development is needed to design models that allow both high predictive accuracy and inferential analysis. Fifth, data were not available for all countries or years, and no data were available for Cape Verde, Libya, or South Africa. In some areas, such as conflict zones or outbreak settings (eg, in northeastern Nigeria), data availability might have been further reduced because of safety and security concerns. If the associations between covariates and vaccine coverage in such areas are fundamentally different than in areas where data are available, these estimates might be biased. Sixth, we defined second-level administrative units by use of boundary definitions from the GAUL database, but changes in subnational administrative boundaries are not always captured in publicly available boundary definitions. Updated, accurate, and publicly available boundary definitions are needed to best assess progress towards subnational GVAP targets. Finally, local coverage estimates can help to guide policy decisions but should be considered in a broader local health context. This model does not account for vaccine cost-effectiveness, local disease burden, competing priorities, or other supply-and-demand side factors that policy makers should consider when allocating resources.

The global community has made a worthwhile, substantial, and ongoing investment to support and expand vaccination services in Africa and around the world. However, despite substantial gains in DPT coverage in Africa between 2000 and 2016, geographical inequalities persist—both within countries and across national borders. To ensure continued progress and improved equality, local, national, and global vaccine delivery programmes require coverage estimates that measure and track coverage at policy-relevant scales. These continent-wide local-resolution estimates provide a novel precision public health tool to assess local patterns of DPT coverage in Africa, monitor progress towards district-level GVAP targets, and triangulate survey-based estimates with subnational administrative data. Local estimates of vaccine coverage can ultimately support more precise targeting of resources to ensure that all children have access to the essential health benefits of vaccination.

## Data sharing

This study follows the Guidelines for Accurate and Transparent Health Estimates Reporting (GATHER). The source code used to generate estimates is accessible online. The study data, including full sets of estimates at the first and second administrative levels, are available online.
