# Supplementary material for: Mapping diphtheria-pertussis-tetanus vaccine coverage in Africa, 2000–2016: a spatial and temporal modelling study
Source: Lancet. 2019 May 4;393(10183):1843–55. doi: 10.1016/S0140-6736(19)30226-0 (PMC6497987; doi:10.1016/S0140-6736(19)30226-0)
Supplement: Supplementary appendix [file mmc1.pdf]

THE LANCET

Supplementary appendix

This appendix formed part of the original submission and has been peer reviewed. We post it as supplied by the authors.

Supplement to: Mosser JF, Gagne-Maynard W, Rao PC, et al. Mapping diphtheria-pertussis-tetanus vaccine coverage in Africa, 2000–2016: a spatial and temporal modelling study. *Lancet* 2019; published online April 5. http://dx.doi.org/10.1016/S0140-6736(19)30226-0.

Supplementary Information for
Mapping diphtheria-pertussis-tetanus vaccine coverage in Africa, 2000-2016: a spatial and temporal modelling study

Table of Contents

Figures and Tables 2
1.0 eFigures 2
2.0 Supplementary Figures 2
3.0 Supplementary Tables 3
Supplementary Materials 13
1.0 GATHER checklist 13
2.0 STROBE checklist 13
3.0 Data Sources and Processing 15
3.1 Data sources excluded from model 35
3.2 Data preparation 35
3.3 Geographic positioning of clusters and spatial integration of areal data 38
4.0 Covariates 40
5.0 Geostatistical Model 44
5.1 Overview of modelling process 44
5.2 Regional model geographies 46
5.3 Covariate ensemble modelling using stacked generalization 46
5.3.1 Covariate extrapolation 49
5.3.2 Relative covariate importance 51
5.4 Geostatistical Model 54
5.4.1 Model description 54
5.4.2 Priors 55
5.4.3 Spatial mesh construction 56
5.4.4 Model fitting and estimate generation 56
5.4.5 Conditional ordinal regression modelling 57
5.5 Model validation 68
5.5.1 Metrics of predictive validity 68
5.5.2 Model comparisons 78
5.6 Post-estimation calibration to national estimates from the Global Burden of Disease Study 83
5.6.1 Production of GBD national coverage estimates 83
5.6.2 Calibration of geospatial estimates to GBD national coverage estimates 84
6.0 Comparison of results to existing estimates 86
6.1 Comparison of pre-calibration geospatial model to GBD estimates 86
6.2 Comparison to DHS estimates 89
6.3 Comparison to Gavi Full Country Evaluations Project small area estimates 93
6.0 References 98

Figures and Tables

1.0 eFigures

eFigure 1: Data availability 4
eFigure 2: Modelling regions used for geospatial estimation of DPT coverage. 5
eFigure 3: Estimated changes in DPT3 coverage between 2000-2016, by second-level administrative unit. 6
eFigure 4: Changes in DPT1 coverage in Africa, 2000-2016. 7
eFigure 5: Administrative-level DPT1 coverage in Africa, 2016 8
eFigure 6: Relative DPT1-DPT3 dropout in Africa, 2000-2016. 9
eFigure 7: Absolute DPT1-DPT3 dropout in Africa, 2000-2016. 10
eFigure 8: Estimated changes in DPT1-3 absolute dropout in Africa, 2000-2016. 11
eFigure 9: Administrative-level absolute and relative DPT1-DPT3 dropout in Africa, 2016. 12

2.0 Supplementary Figures

Supplementary Figure 1: Flow diagram of survey identification and data loss during data cleaning. 37
Supplementary Figure 2: Weighted DPT3 coverage, by age cohort and survey. 38
Supplementary Figure 3: Spatial resampling of areal data 40
Supplementary Figure 4: Maps of included high-resolution covariates. 43
Supplementary Figure 5: Overview of modelling process 45
Supplementary Figure 6: Example of covariate ensemble modelling 46
Supplementary Figure 7: Predicted in-sample DPT3 surfaces from the ensemble covariate modelling progress, 2016. 48
Supplementary Figure 8: Covariate extrapolation by model region 49
Supplementary Figure 9: Relative covariate importance plots. 51
Supplementary Figure 10: Example finite elements mesh. 56
Supplementary Figure 11: Ternary plot of estimates of the distribution of vaccination at a single 5x5 km pixel, by draw 59
Supplementary Figure 12: Model uncertainty for DPT3 coverage, 2000 – 2016. 60
Supplementary Figure 13: Model uncertainty for DPT1 coverage, 2000-2016 61
Supplementary Figure 14: Model uncertainty for DPT1-3 absolute dropout, 2000-2016 62
Supplementary Figure 15: Comparison of uncertainty and coverage, 2016. 63
Supplementary Figure 16: Beta coefficients for ensemble sub-models. 67
Supplementary Figure 17: Visualization of spatial holdouts at the second administrative level 69
Supplementary Figure 18: Out-of-sample 95% coverage of predictive intervals. 70
Supplementary Figure 19: Out-of-sample mean absolute error. 70
Supplementary Figure 20: Out-of-sample mean error (bias). 71
Supplementary Figure 21: Out-of-sample RMSE 71
Supplementary Figure 22: Out-of-sample predictions of DPT3 coverage vs. observed data at the second administrative level 72
Supplementary Figure 23: Out-of-sample predictions of DPT1 coverage vs. observed data at the second administrative level 73
Supplementary Figure 24: Out-of-sample predictions of DPT1-3 absolute dropout vs. observed data at the second administrative level 74
Supplementary Figure 25: Residual error maps for DPT3 coverage 75
Supplementary Figure 26: Residual error maps for DPT1 coverage 76
Supplementary Figure 27: Residual error maps for DPT 1-3 absolute dropout 77
Supplementary Figure 28: Out-of-sample model comparisons for DPT3 coverage 79
Supplementary Figure 37: Out-of-sample model comparisons for DPT1 coverage 80
Supplementary Figure 38: Out-of-sample model comparisons for DPT1-3 absolute dropout comparisons 81
Supplementary Figure 39: Estimated DPT3 coverage for each comparison model, 2016. 82
Supplementary Figure 40: Ternary plot of example pixel-level calibration to GBD estimates 85

Supplementary Figure 41: Comparison of national-level estimates of DPT3 coverage from model-based geostatistics (MBG) and the Global Burden of Disease study (GBD). 87
Supplementary Figure 42: Comparison of national-level estimates of DPT1 coverage from model-based geostatistics (MBG) and the Global Burden of Disease study (GBD). 88
Supplementary Figure 43: Comparison of national-level estimates of DPT1-3 absolute dropout from model-based geostatistics (MBG) and the Global Burden of Disease study (GBD). 89
Supplementary Figure 45: Comparison of calibrated model-based geostatistical (MBG) and DHS estimates of DPT3 coverage at the first administrative level. 90
Supplementary Figure 47: Comparison of calibrated model-based geostatistical (MBG) and DHS estimates of DPT1 coverage at the first administrative level. 91
Supplementary Figure 49: Comparison of calibrated model-based geostatistical (MBG) and DHS estimates of DPT 1-3 absolute dropout at the first administrative level. 92
Supplementary Figure 50: Comparison of MBG and Gavi FCE second-administrative-level estimates of DPT3 coverage for Cameroon, 2000-2015. 93
Supplementary Figure 51: Comparison of MBG and Gavi FCE second-administrative-level estimates of DPT3 coverage for Mozambique, 2000-2015. 94
Supplementary Figure 52: Comparison of MBG and Gavi FCE second-administrative-level estimates of DPT3 coverage for Chad, 2000-2015. 95
Supplementary Figure 53: Comparison of MBG and Gavi FCE second-administrative-level estimates of DPT3 coverage for Uganda, 2000-2015. 96
Supplementary Figure 54: Comparison of MBG and Gavi FCE second-administrative-level estimates of DPT3 coverage for Zambia, 2000-2015. 97

3.0 Supplementary Tables

Supplementary Table 1: Household surveys used in mapping vaccine coverage. 16
Supplementary Table 2: Data excluded from both the geostatistical model and GBD estimates 34
Supplementary Table 3: Data excluded from GBD estimates but included in geostatistical model 34
Supplementary Table 4: Covariates used in mapping of vaccine coverage. 41
Supplementary Table 5: Spatial hyperparameter priors selected by R-INLA, by region 55
Supplementary Table 6: Fitted geostatistical model parameters for DTP3 coverage 64
Supplementary Table 7: Fitted geostatistical model parameters for the 2-dose conditional coverage model 65
Supplementary Table 8: Fitted geostatistical model parameters for the 1-dose conditional coverage model 66

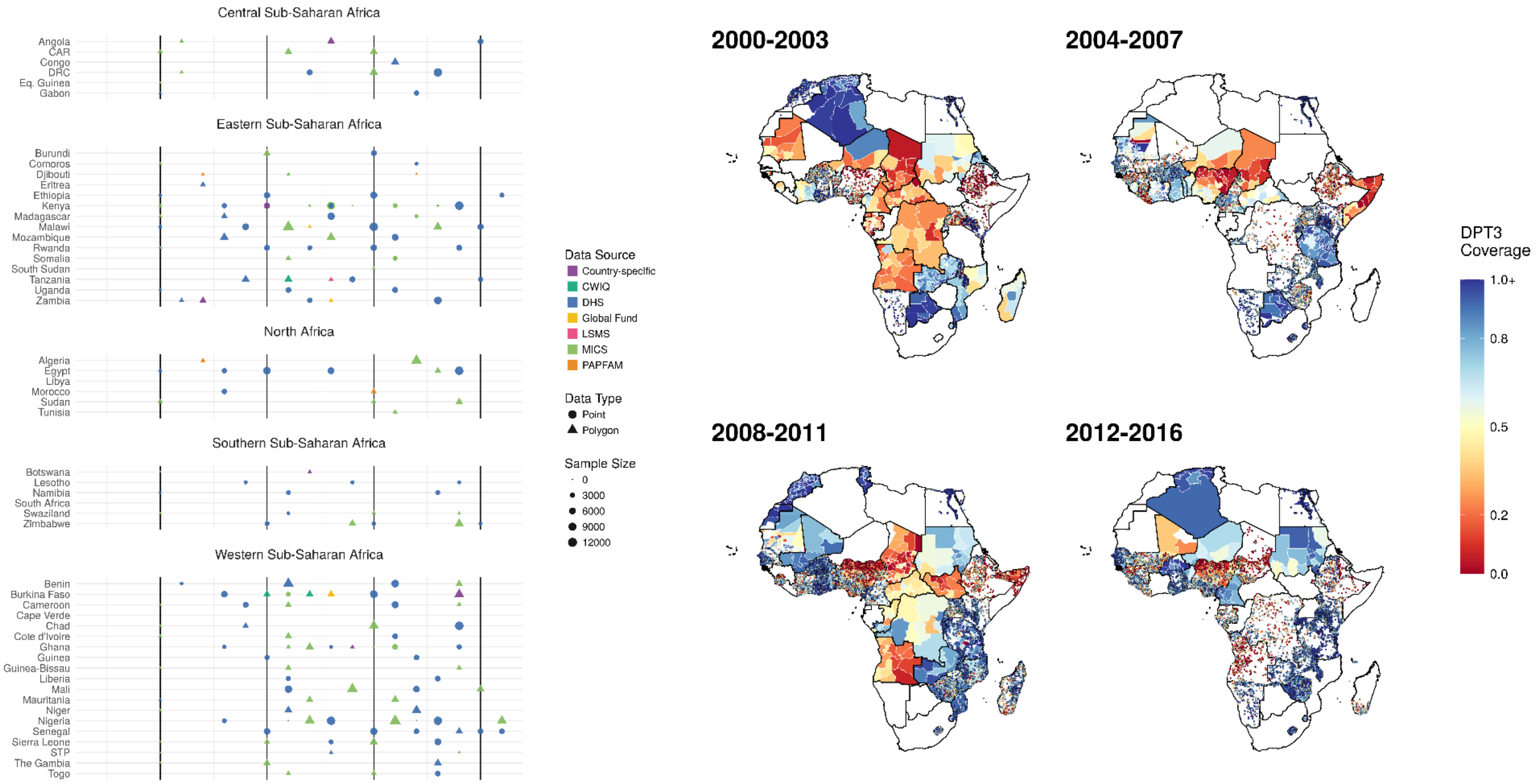

eFigure 1: Data availability

Data availability for DPT3 coverage. Left: scatter plot indicating the source, spatial resolution, and sample size of each survey included in the model. Right: spatial and temporal data availability, with the colour of each point or polygon representing the raw DPT3 coverage in the surveyed cluster or areal unit. Data used for modelling of the conditional vaccine coverage indicators was derived from the total data set shown here. Data sources are categorized by survey series as follows: Core Welfare Indicators Questionnaire Survey (CWIQ), Demographic and Health Survey (DHS), Global Fund Household Health Coverage Survey (Global Fund), Living Standards Measurement Study (LSMS), Multiple Indicator Cluster Survey (MICS), Pan Arab Project for Family Health (PAPFAM), or other country-specific surveys (see Supplementary Table 1 for details).

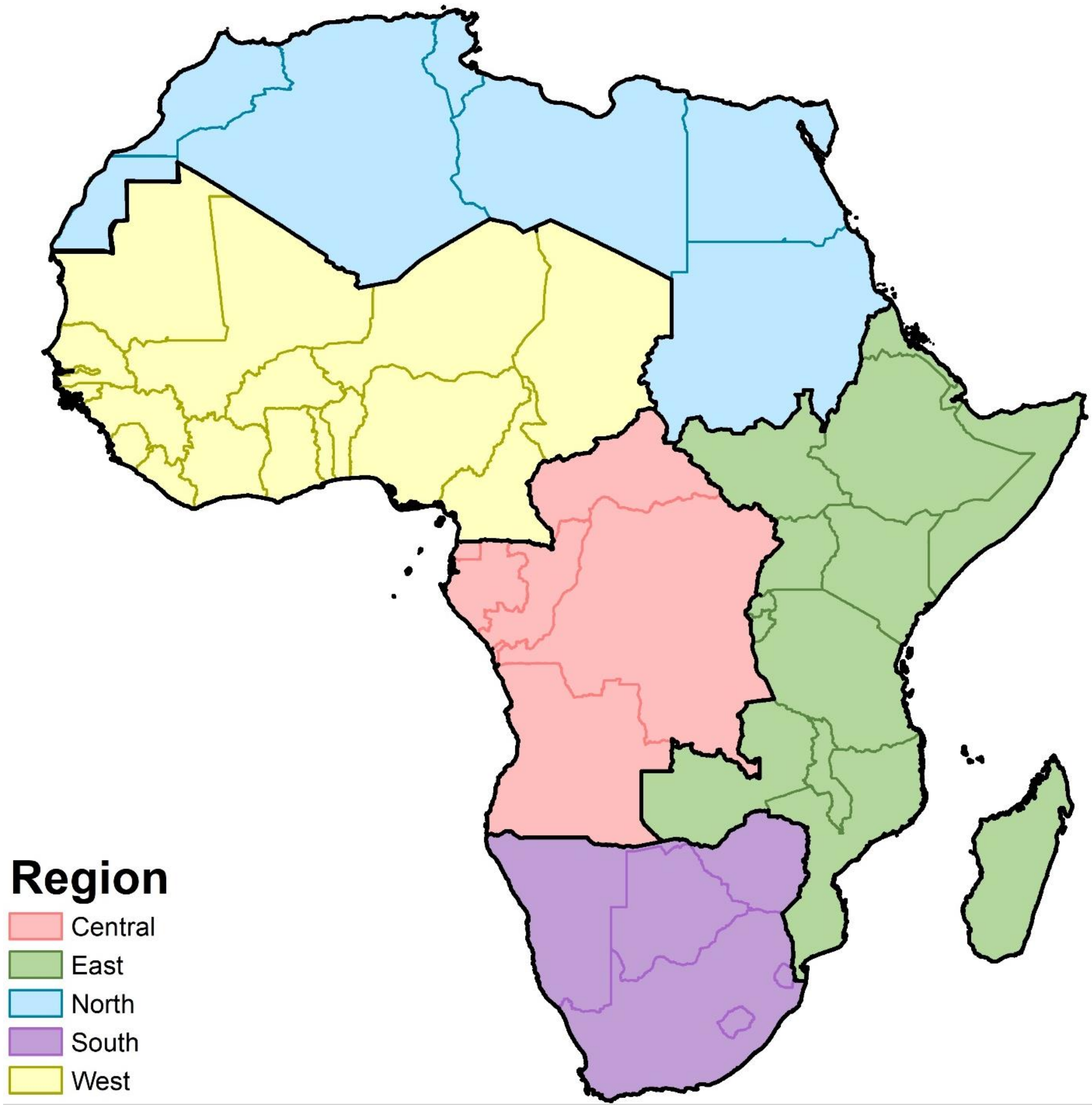

eFigure 2: Modelling regions used for geospatial estimation of DPT coverage.
Modelling regions were derived from five GBD regions: North Africa and the Middle East, Central sub-Saharan Africa, Eastern sub-Saharan Africa, Western sub-Saharan Africa, and Southern sub-Saharan Africa. The GBD region of North Africa and the Middle East includes several countries outside of the geographic scope of this study, which were excluded: Afghanistan, Bahrain, Iran, Iraq, Jordan, Kuwait, Lebanon, Oman, Palestine, Qatar, Saudi Arabia, Syria, Turkey, UAE, and Yemen.

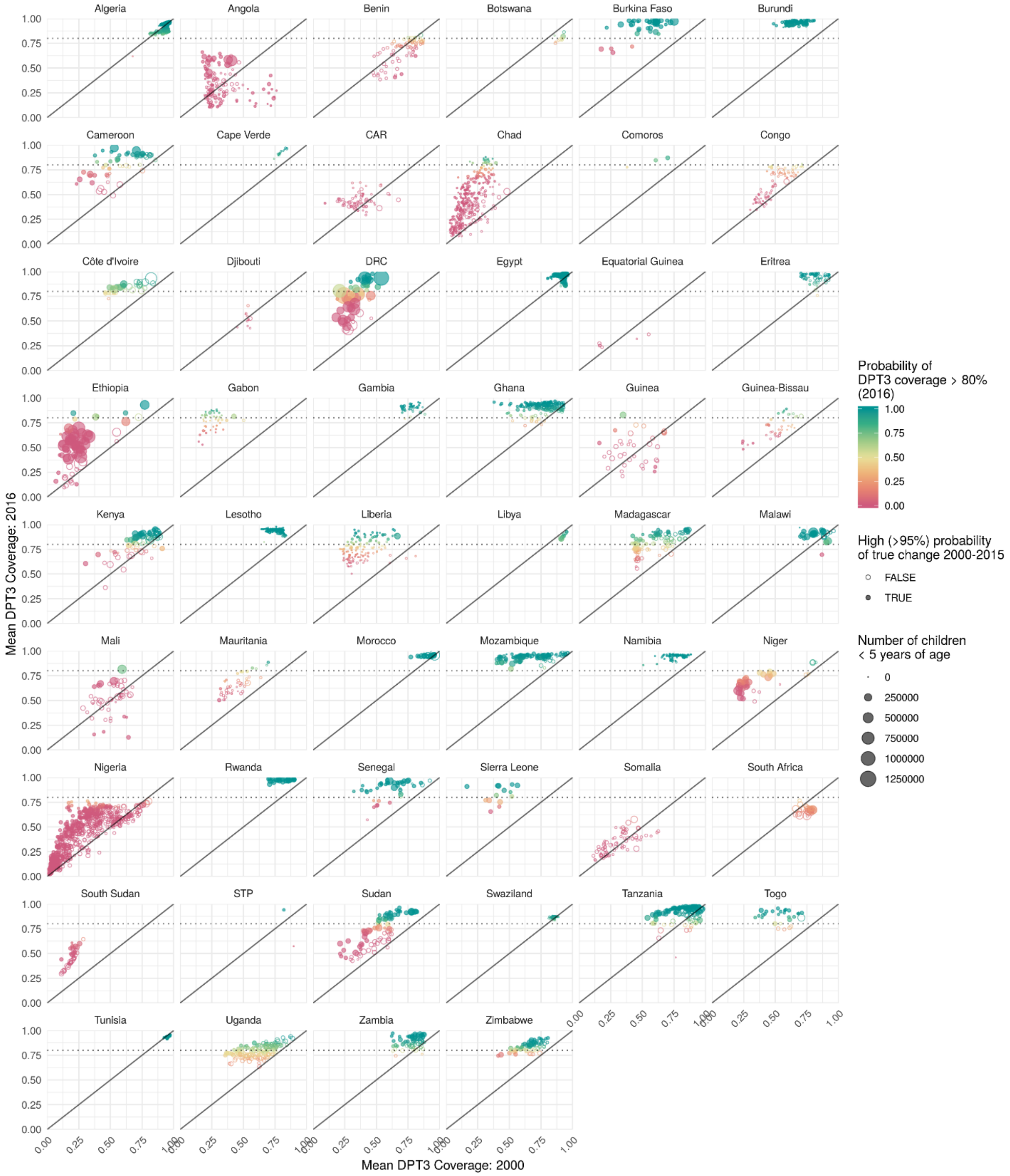

eFigure 3: Estimated changes in DPT3 coverage between 2000-2016, by second-level administrative unit.

Each point represents a single second-level administrative unit, with area proportional to the number of children < 5 years of age and colour representing the posterior probability of DPT3 coverage > 80% in that administrative unit in 2016. Administrative units with a high (> 95%) probability of having experienced a true increase or decrease in DPT3 coverage between 2000-2016 are rendered as filled points, while administrative units with intermediate probabilities of a true change are rendered as hollow circles.

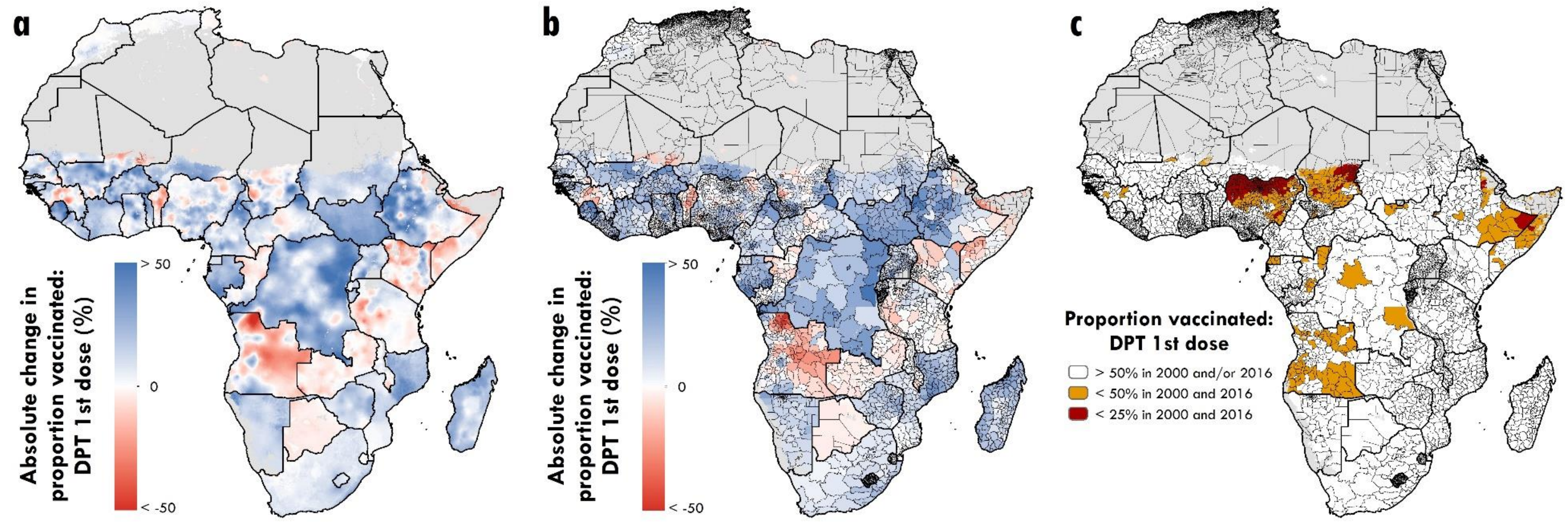

eFigure 4: Changes in DPT1 coverage in Africa, 2000-2016.

Mean estimated change in DPT1 coverage among 12-23 month old children at the 5x5 km resolution between 2000 and 2016 at the 5x5 km (a) and second administrative level (b). Colours represent estimated absolute change, in percentage points, where positive changes (coverage increases) are represented in blue and negative changes (coverage decreases) in red. Increases and decreases of ≥ 50 percentage points are represented by dark blue and dark red, respectively. Panel (c) displays areas of low DPT1 coverage over time. Second administrative units with coverage < 25% in both 2000 and 2016 are represented in red; additional units with coverage < 50% in both 2000 and 2016 are represented in orange. Results are masked in grey where total population density was less than 10 individuals per 1 km pixel in 2015 per WorldPop[1] estimates, or where land cover was classified as “barren or sparsely vegetated” based on MODIS[2] satellite data in 2013.

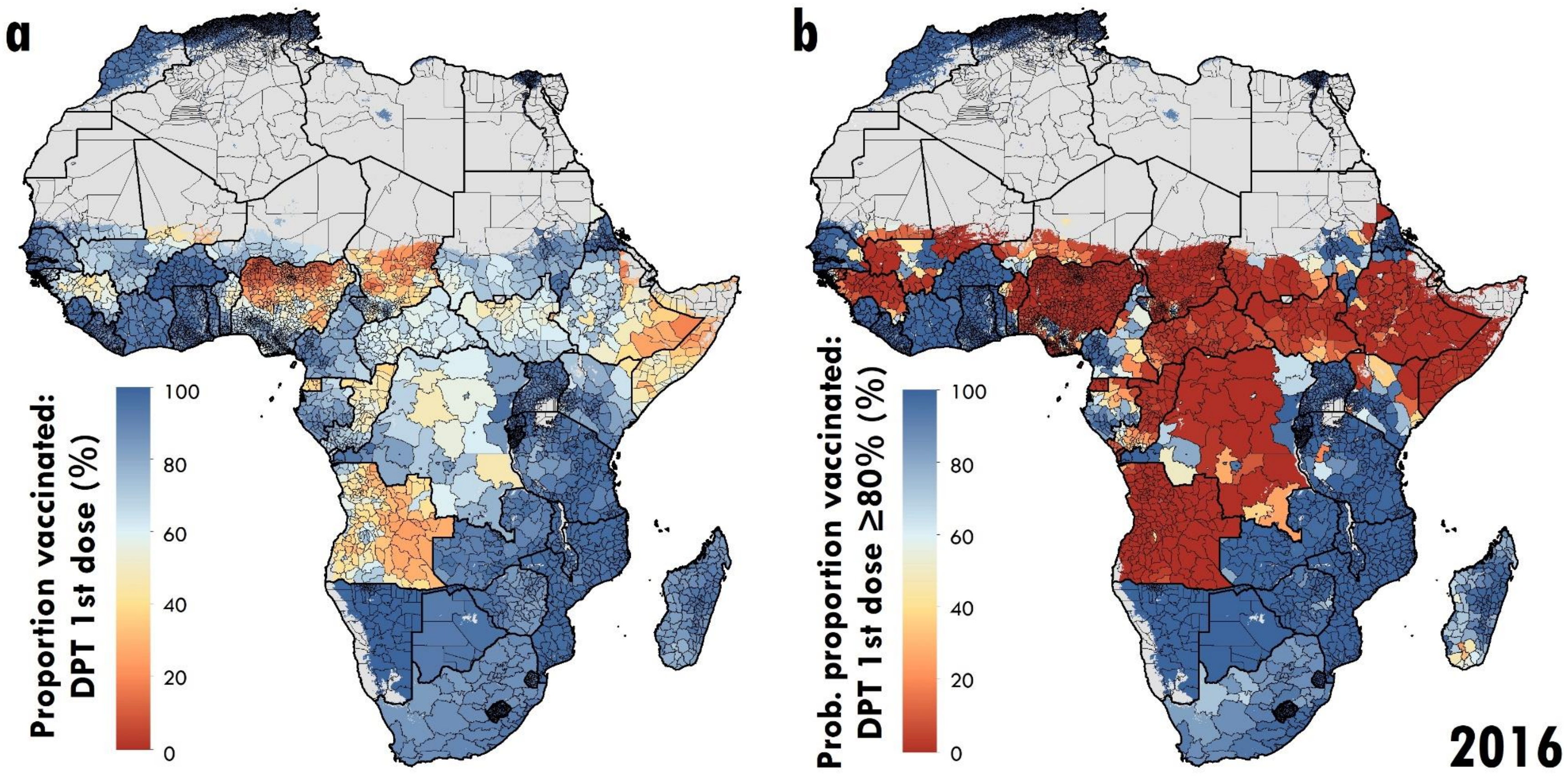

eFigure 5: Administrative-level DPT1 coverage in Africa, 2016

Estimated DPT1 coverage among 12-23 month old children at the second administrative level (a); probability of second-level administrative unit achieving ≥ 80% DPT1 coverage in 2016 (c). Results are masked in grey where total population density was less than 10 individuals per 1x1 km pixel in 2015 per WorldPop[1] estimates, or where land cover was classified as "barren or sparsely vegetated" based on MODIS[2] satellite data in 2013.

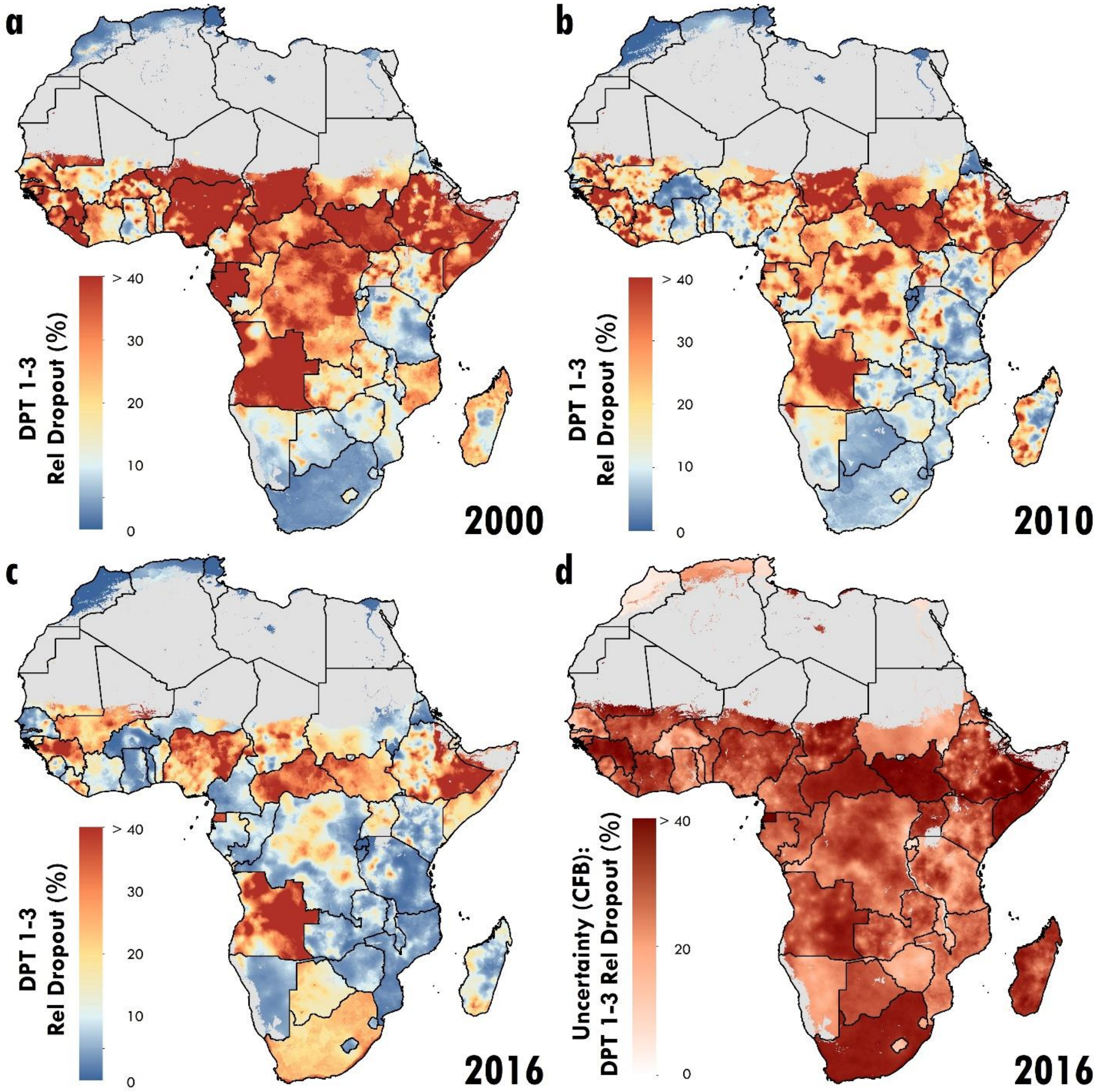

eFigure 6: Relative DPT1-DPT3 dropout in Africa, 2000-2016.
Estimated DPT1-DPT3 relative dropout among 12-23 month old children at the 5x5 kilometre resolution in 2000, 2010, and 2016 (a-c); model uncertainty in 2016 (d). Model uncertainty is displayed using the Coffey-Feingold-Bromberg metric (CFB), a measure of uncertainty that is comparable regardless of mean coverage and scales from 0% (no uncertainty) to 100% (highest possible uncertainty for a given mean). Results are masked in grey where total population density was less than 10 individuals per 1 km pixel in 2015 per WorldPop[1] estimates, or where land cover was classified as "barren or sparsely vegetated" based on MODIS[2] satellite data in 2013.

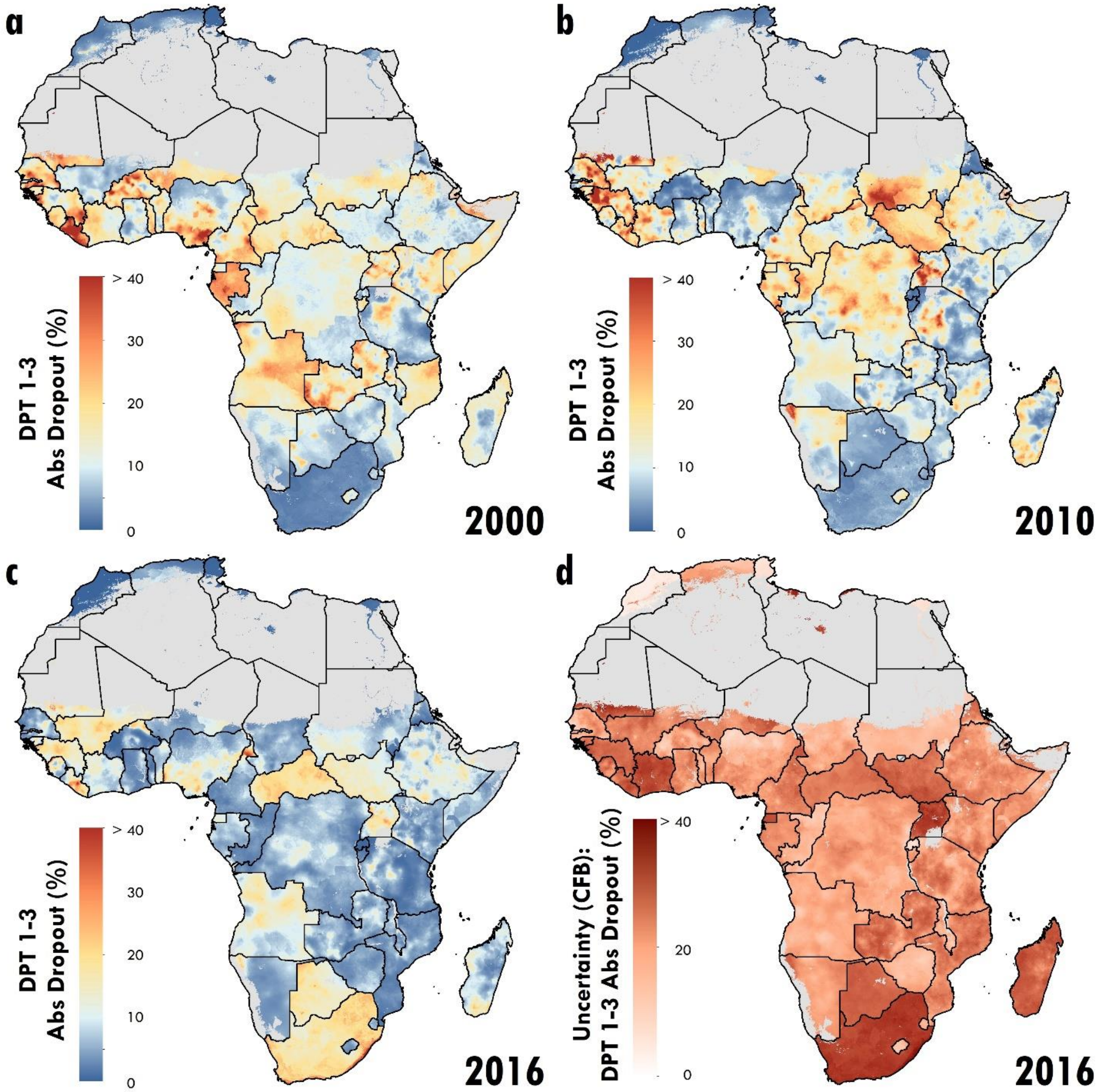

eFigure 7: Absolute DPT1-DPT3 dropout in Africa, 2000-2016.
Estimated DPT1-DPT3 relative dropout among 12-23 month old children at the 5x5 kilometre resolution in 2000, 2010, and 2016 (a-c); model uncertainty in 2016 (d). Model uncertainty is displayed using the Coffey-Feingold-Bromberg metric (CFB), a measure of uncertainty that is comparable regardless of mean coverage and scales from 0% (no uncertainty) to 100% (highest possible uncertainty for a given mean). Results are masked in grey where total population density was less than 10 individuals per 1 km pixel in 2015 per WorldPop[1] estimates, or where land cover was classified as “barren or sparsely vegetated” based on MODIS[2] satellite data in 2013.

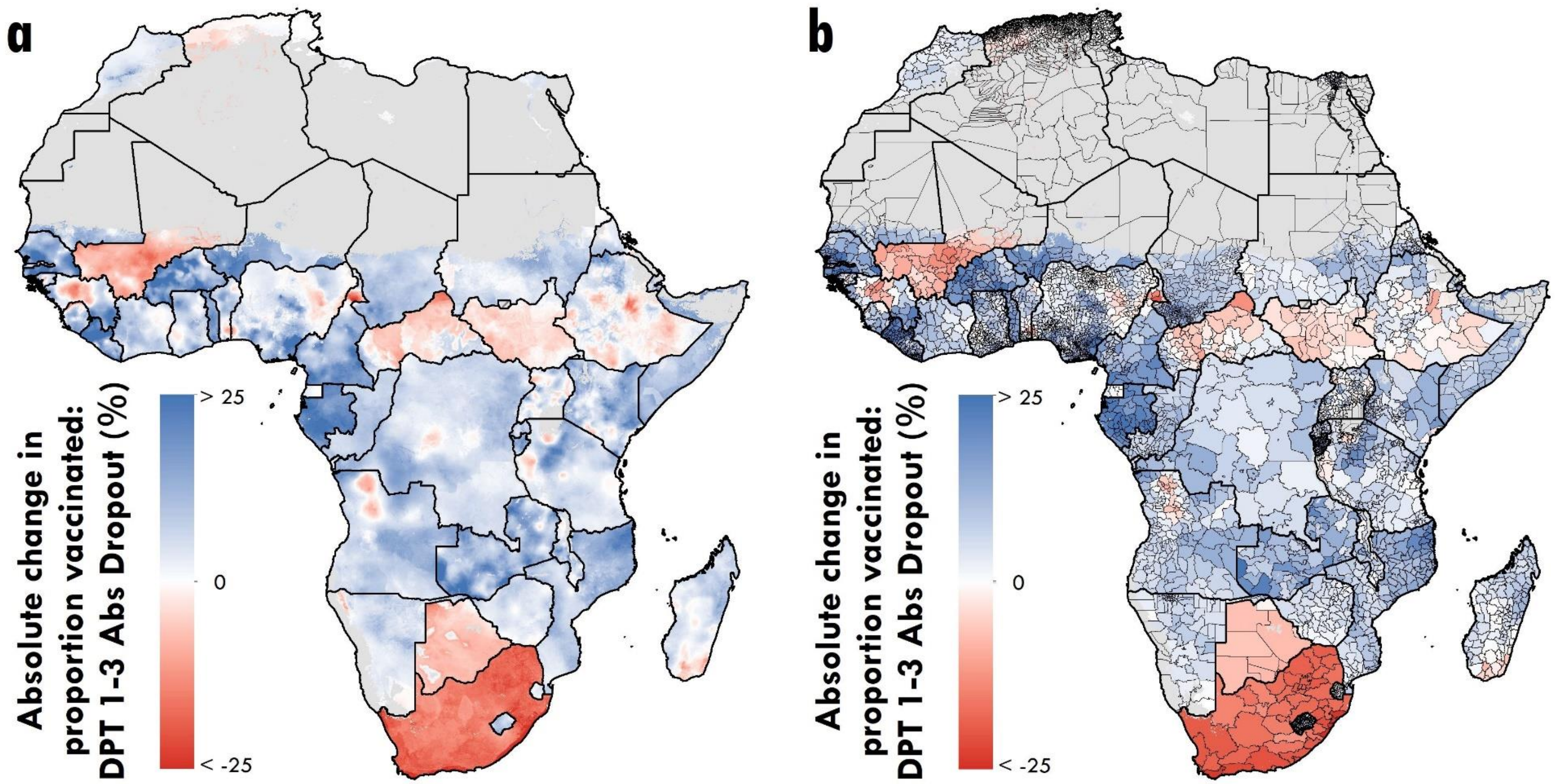

eFigure 8: Estimated changes in DPT1-3 absolute dropout in Africa, 2000-2016.
Mean estimated change in DPT1-3 absolute dropout among 12-23 month old children at the 5x5 kilometre resolution between 2000 and 2016 at the 5x5 km (a) and second administrative level (b). Results are masked in grey where total population density was less than 10 individuals per 1 km pixel in 2015 per WorldPop[1] estimates, or where land cover was classified as "barren or sparsely vegetated" based on MODIS[2] satellite data in 2013.

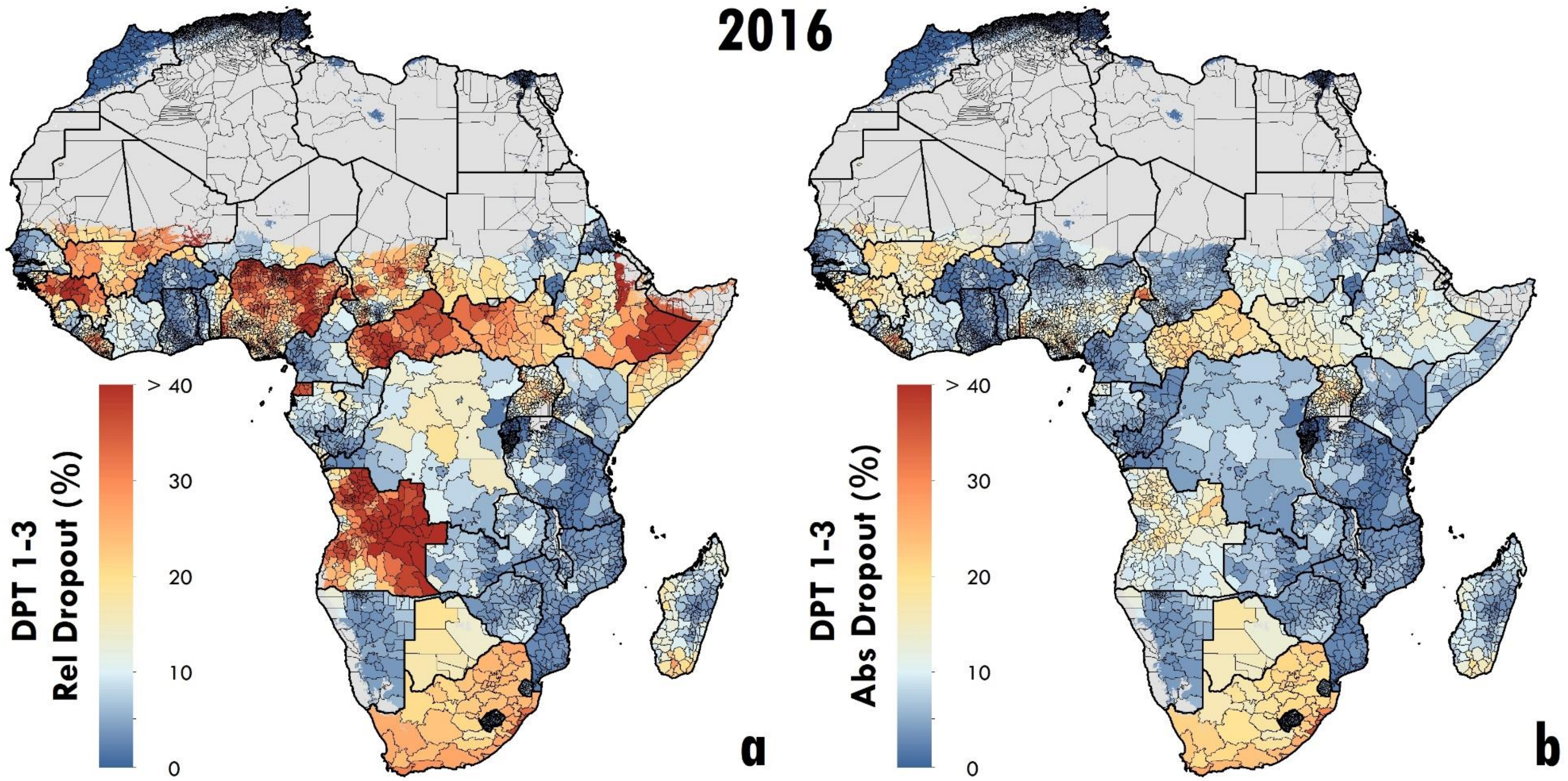

eFigure 9: Administrative-level absolute and relative DPT1-DPT3 dropout in Africa, 2016.
Estimated DPT1-DPT3 relative dropout (a) and absolute dropout (b) among 12-23 month old children at the second administrative level in 2016. Results are masked in grey where total population density was less than 10 individuals per 1 km pixel in 2015 per WorldPop[1] estimates, or where land cover was classified as "barren or sparsely vegetated" based on MODIS[2] satellite data in 2013.

Supplementary Materials

1.0 GATHER checklist

Item #	Checklist item	Reported on page #
**Objectives and funding**		
**1**	Define the indicator(s), populations (including age, sex, and geographic entities), and time period(s) for which estimates were made.	7-9
**2**	List the funding sources for the work.	18
**Data Inputs**		
For all data inputs from multiple sources that are synthesized as part of the study:		
**3**	Describe how the data were identified and how the data were accessed.	7-8
**4**	Specify the inclusion and exclusion criteria. Identify all ad-hoc exclusions.	7-8; Supplementary Information (SI) 34-35
**5**	Provide information on all included data sources and their main characteristics. For each data source used, report reference information or contact name/institution, population represented, data collection method, year(s) of data collection, sex and age range, diagnostic criteria or measurement method, and sample size, as relevant.	SI 16-43
**6**	Identify and describe any categories of input data that have potentially important biases (e.g., based on characteristics listed in item 5).	SI 18-44
For data inputs that contribute to the analysis but were not synthesized as part of the study:		
**7**	Describe and give sources for any other data inputs.	SI 48-51
For all data inputs:		
**8**	Provide all data inputs in a file format from which data can be efficiently extracted (e.g., a spreadsheet rather than a PDF), including all relevant meta-data listed in item 5. For any data inputs that cannot be shared because of ethical or legal reasons, such as third-party ownership, provide a contact name or the name of the institution that retains the right to the data.	SI 18-43
**Data analysis**		
**9**	Provide a conceptual overview of the data analysis method. A diagram may be helpful.	SI 45
**10**	Provide a detailed description of all steps of the analysis, including mathematical formulae. This description should cover, as relevant, data cleaning, data pre-processing, data adjustments and weighting of data sources, and mathematical or statistical model(s).	SI 44-46, 53-92
**11**	Describe how candidate models were evaluated and how the final model(s) were selected.	9, SI 68-82
**12**	Provide the results of an evaluation of model performance, if done, as well as the results of any relevant sensitivity analysis.	SI 68-92
**13**	Describe methods for calculating uncertainty of the estimates. State which sources of uncertainty were, and were not, accounted for in the uncertainty analysis.	7, SI 61-62
**14**	State how analytic or statistical source code used to generate estimates can be accessed.	7
**Results and Discussion**		
**15**	Provide published estimates in a file format from which data can be efficiently extracted.	7
**16**	Report a quantitative measure of the uncertainty of the estimates (e.g. uncertainty intervals).	7-11
**17**	Interpret results in light of existing evidence. If updating a previous set of estimates, describe the reasons for changes in estimates.	11-14
**18**	Discuss limitations of the estimates. Include a discussion of any modelling assumptions or data limitations that affect interpretation of the estimates.	13-14

2.0 STROBE checklist

	Item No	Recommendation	Reported on Page #
**Title and abstract**	**1**	(*a*) Indicate the study's design with a commonly used term in the title or the abstract	1-3
		(*b*) Provide in the abstract an informative and balanced summary of what was done and what was found	3
**Introduction**			**6**
Background/rationale	**2**	Explain the scientific background and rationale for the investigation being reported	6
Objectives	**3**	State specific objectives, including any prespecified hypotheses	7
**Methods**			**7**

Study design	**4**	Present key elements of study design early in the paper	7-9
Setting	**5**	Describe the setting, locations, and relevant dates, including periods of recruitment, exposure, follow-up, and data collection	7-9
Participants	**6**	(*a*) *Cohort study*—Give the eligibility criteria, and the sources and methods of selection of participants. Describe methods of follow-up<br>*Case-control study*—Give the eligibility criteria, and the sources and methods of case ascertainment and control selection. Give the rationale for the choice of cases and controls<br>*Cross-sectional study*—Give the eligibility criteria, and the sources and methods of selection of participants	7-8, Supplementary Information (SI) 24-42
		(*b*) *Cohort study*—For matched studies, give matching criteria and number of exposed and unexposed.31<br>*Case-control study*—For matched studies, give matching criteria and the number of controls per case	NA
Variables	**7**	Clearly define all outcomes, exposures, predictors, potential confounders, and effect modifiers. Give diagnostic criteria, if applicable	7-9
Data sources/ measurement	**8***	For each variable of interest, give sources of data and details of methods of assessment (measurement). Describe comparability of assessment methods if there is more than one group	7-9,SI 17-43
Bias	**9**	Describe any efforts to address potential sources of bias	SI 18-44
Study size	**10**	Explain how the study size was arrived at	7-8
Quantitative variables	**11**	Explain how quantitative variables were handled in the analyses. If applicable, describe which groupings were chosen and why	8-9
Statistical methods	**12**	(*a*) Describe all statistical methods, including those used to control for confounding	8-9
		(*b*) Describe any methods used to examine subgroups and interactions	8-9
		(*c*) Explain how missing data were addressed	8-9
		(*d*) *Cohort study*—If applicable, explain how loss to follow-up was addressed<br>*Case-control study*—If applicable, explain how matching of cases and controls was addressed<br>*Cross-sectional study*—If applicable, describe analytical methods taking account of sampling strategy	SI 44-46, 53-92
		(*e*) Describe any sensitivity analyses	SI 68-92

**Results**			
Participants	**13***	(a) Report numbers of individuals at each stage of study—eg numbers potentially eligible, examined for eligibility, confirmed eligible, included in the study, completing follow-up, and analysed	7, SI 43
		(b) Give reasons for non-participation at each stage	Non-participation reasons largely unknown; see individual survey reports for details.
		(c) Consider use of a flow diagram	SI 43
Descriptive data	**14***	(a) Give characteristics of study participants (eg demographic, clinical, social) and information on exposures and potential confounders	Varies by survey; see individual survey reports for details
		(b) Indicate number of participants with missing data for each variable of interest	SI 43
		(c) *Cohort study*—Summarise follow-up time (eg, average and total amount)	NA
Outcome data	**15***	*Cohort study*—Report numbers of outcome events or summary measures over time	NA
		Case-control study—Report numbers in each exposure category, or summary measures of exposure	NA
		Cross-sectional study—Report numbers of outcome events or summary measures	SI 44-46, 53-92
Main results	**16**	(*a*) Give unadjusted estimates and, if applicable, confounder-adjusted estimates and their precision (eg, 95% confidence interval). Make clear which confounders were adjusted for and why they were included	7, SI 61-62
		(*b*) Report category boundaries when continuous variables were categorized	SI 35, SI 40
		(*c*) If relevant, consider translating estimates of relative risk into absolute risk for a meaningful time period	NA
Other analyses	**17**	Report other analyses done—eg analyses of subgroups and interactions, and sensitivity analyses	SI 68-92

Discussion			14
Key results	**18**	Summarise key results with reference to study objectives	3-5
Limitations	**19**	Discuss limitations of the study, taking into account sources of potential bias or imprecision. Discuss both direction and magnitude of any potential bias	16-17
Interpretation	**20**	Give a cautious overall interpretation of results considering objectives, limitations, multiplicity of analyses, results from similar studies, and other relevant evidence	14-16
Generalisability	**21**	Discuss the generalisability (external validity) of the study results	14-15
**Other information**			
Funding	**22**	Give the source of funding and the role of the funders for the present study and, if applicable, for the original study on which the present article is based	17

3.0 Data Sources and Processing

The sources of data used to model vaccine coverage are described below in Supplementary Table 1 along with the number of individuals and clusters included as well as information about the spatial resolution available (GPS-located or areal data). Of 183 surveys, 88 were from the Demographic and Health (DHS) series, 73 from the UNICEF Multiple Indicator Cluster Survey (MICS) series, and 22 from other sources. eFigure 1 shows the spatial and temporal extent of data availability by country.

Note that in three countries (South Africa, Libya, and Cape Verde), no data were identified meeting the inclusion and exclusion criteria described below. In these places, coverage estimates are produced from the relationships between DPT coverage and covariates (from other countries within the modelling region) and national-level estimates of coverage from the Global Burden of Disease study.

Supplementary Table 1: Household surveys used in mapping vaccine coverage.
Sources can be located in the Global Health Data Exchange (GHDx, http://ghdx.healthdata.org) through the provided hyperlinks. The GHDx contains additional information about each survey source and links to the underlying source data where publicly available.

Country	GPS-located clusters	Areally-located clusters	Number of children included	Series	Year(s)	Citation	Link
Algeria	0	496	1911	Pan Arab Project for Family Health (PAPFAM)	2002-2003	National Office of Statistics (Algeria), Ministry of Health, Population and Hospital Reform (Algeria), League of Arab States. Algeria Family Health Survey 2002-2003.	GHDx
Algeria	0	1119	11042	Multiple Indicator Cluster Survey (MICS)	2012-2013	Ministry of Health and Population (Algeria), United Nations Children's Fund (UNICEF). Algeria Multiple Indicator Cluster Survey 2012-2013. New York, United States: United Nations Children's Fund (UNICEF), 2016.	GHDx
Angola	0	330	2240	Multiple Indicator Cluster Survey (MICS)	2001	National Institute of Statistics (Angola), United Nations Children's Fund (UNICEF). Angola Multiple Indicator Cluster Survey 2001. New York, United States: United Nations Children's Fund (UNICEF).	GHDx
Angola	0	18	6167	Angola Integrated Inquiry into People's Well-Being	2008-2009	National Institute of Statistics (Angola), Oxford Policy Management, United Nations Children's Fund (UNICEF). Angola Integrated Inquiry into People's Well-Being 2008-2009.	GHDx
Angola	624	0	5425	Demographic and Health Survey (DHS)	2015-2016	ICF International, Ministry of Health (Angola), National Institute of Statistics (Angola), United Nations Children's Fund (UNICEF). Angola Demographic and Health Survey 2015-2016. Fairfax, United States: ICF International, 2017.	GHDx
Benin	243	0	1854	Demographic and Health Survey (DHS)	2001	National Institute of Statistics and Economic Analysis (INSAE) (Benin), ORC Macro. Benin Demographic and Health Survey 2001. Calverton, United States: ORC Macro.	GHDx
Benin	0	750	11234	Demographic and Health Survey (DHS)	2006	National Institute of Statistics and Economic Analysis (INSAE) (Benin), National Program Against AIDS (PNLS) (Benin), Macro International, Inc. Benin Demographic and Health Survey 2006. Calverton, United States: Macro International, Inc.	GHDx
Benin	746	0	9844	Demographic and Health Survey (DHS)	2011-2012	ICF International, National Institute of Statistics and Economic Analysis (INSAE) (Benin), National Program Against AIDS (PNLS) (Benin). Benin Demographic and Health Survey 2011-2012. Fairfax, United States: ICF International, 2014.	GHDx
Benin	0	723	4612	Multiple Indicator Cluster Survey (MICS)	2014	National Institute of Statistics and Economic Analysis (INSAE) (Benin), United Nations Children's Fund (UNICEF). Benin Multiple Indicator Cluster Survey 2014. New York, United States: United Nations Children's Fund (UNICEF), 2017.	GHDx

Country	GPS-located clusters	Areally-located clusters	Number of children included	Series	Year(s)	Citation	Link
Botswana	0	21	544	Multiple Indicator Cluster Survey (MICS)	2000	Central Statistics Office (Botswana), United Nations Children's Fund (UNICEF). Botswana Multiple Indicator Cluster Survey 2000. New York, United States: United Nations Children's Fund (UNICEF), 2015.	GHDx
Botswana	0	358	1790	Botswana Family Health Survey	2007-2008	Central Statistics Office (Botswana). Botswana Family Health Survey 2007-2008. Gaborone, Botswana: Central Statistics Office (Botswana), 2009.	GHDx
Burkina Faso	397	0	7133	Demographic and Health Survey (DHS)	2003	Macro International, Inc, National Institute of Statistics and Demography (Burkina Faso). Burkina Faso Demographic and Health Survey 2003. Calverton, United States: Macro International, Inc.	GHDx
Burkina Faso	0	422	4537	Core Welfare Indicators Questionnaire Survey (CWIQ)	2005	National Institute of Statistics and Demography (Burkina Faso), World Bank. Burkina Faso Core Welfare Indicators Questionnaire Survey 2005. Ouagadougou, Burkina Faso: National Institute of Statistics and Demography (Burkina Faso).	GHDx
Burkina Faso	195	0	3519	Multiple Indicator Cluster Survey (MICS)	2006	National Institute of Statistics and Demography (Burkina Faso), United Nations Children's Fund (UNICEF). Burkina Faso Multiple Indicator Cluster Survey 2006. New York, United States: United Nations Children's Fund (UNICEF).	GHDx
Burkina Faso	0	424	5128	Core Welfare Indicators Questionnaire Survey (CWIQ)	2007	National Institute of Statistics and Demography (INSD). Burkina Faso Core Welfare Indicators Questionnaire Survey 2007. Ouagadougou, Burkina Faso: National Institute of Statistics and Demography (INSD), 2008.	GHDx
Burkina Faso	0	324	5156	Global Fund Household Health Coverage Survey	2008	Global Fund to Fight Aids Tuberculosis and Malaria (GFATM). Burkina Faso Global Fund Household Health Coverage Survey 2008.	GHDx
Burkina Faso	541	0	10132	Demographic and Health Survey (DHS)	2010-2011	ICF Macro, Ministry of Health (Burkina Faso), National Institute of Statistics and Demography (Burkina Faso). Burkina Faso Demographic and Health Survey 2010-2011. Calverton, United States: ICF Macro.	GHDx
Burkina Faso	0	890	9124	Living Standards Measurement Study - Integrated Surveys on Agriculture (LSMS-ISA)	2014	National Institute of Statistics and Demography (Burkina Faso), World Bank. Burkina Faso Continuous Multisectoral Survey 2014. Washington DC, United States: World Bank.	GHDx
Burundi	0	547	4595	Multiple Indicator Cluster Survey (MICS)	2005	United Nations Children's Fund (UNICEF), Burundi Institute of Statistics and Economic Studies, United Nations Population Fund (UNFPA). Burundi Multiple Indicator Cluster Survey 2005. New York, United States: United Nations Children's Fund (UNICEF).	GHDx
Burundi	376	0	5732	Demographic and Health Survey (DHS)	2010-2011	Burundi Institute of Statistics and Economic Studies, ICF International, Ministry of Public Health and the Fight against AIDS (Burundi). Burundi Demographic and Health Survey 2010-2011. Fairfax, United States: ICF International, 2012.	GHDx

Country	GPS-located clusters	Areally-located clusters	Number of children included	Series	Year(s)	Citation	Link
Cameroon	0	140	670	Multiple Indicator Cluster Survey (MICS)	2000	Department of Statistics and Accounting, Ministry of the Economy and Finance (Cameroon) and United Nations Children's Fund (UNICEF). Cameroon Multiple Indicator Cluster Survey 2000. New York, United States: United Nations Children's Fund (UNICEF).	GHDx
Cameroon	460	0	5590	Demographic and Health Survey (DHS)	2004	Macro International, Inc, National Institute of Statistics (Cameroon). Cameroon Demographic and Health Survey 2004. Calverton, United States: Macro International, Inc.	GHDx
Cameroon	0	459	3867	Multiple Indicator Cluster Survey (MICS)	2006	United Nations Children's Fund (UNICEF), National Institute of Statistics (Cameroon). Cameroon Multiple Indicator Cluster Survey 2006. New York, United States: United Nations Children's Fund (UNICEF).	GHDx
Cameroon	576	0	8258	Demographic and Health Survey (DHS)	2011	ICF International, Ministry of Economy, Planning and Regional Development (Cameroon), Ministry of Public Health (Cameroon), National Institute of Statistics (Cameroon), Pasteur Center of Cameroon. Cameroon Demographic and Health Survey 2011. Fairfax, United States: ICF International.	GHDx
Cameroon	0	471	2536	Multiple Indicator Cluster Survey (MICS)	2014	Ministry of Public Health (Cameroon), National Institute of Statistics (Cameroon), United Nations Children's Fund (UNICEF). Cameroon Multiple Indicator Cluster Survey 2014. New York, United States: United Nations Children's Fund (UNICEF), 2017.	GHDx
Central African Republic	0	500	2865	Multiple Indicator Cluster Survey (MICS)	2000	Division of Statistics and Economic Studies (Central African Republic), Ministry of Economy, Planning and International Cooperation (Central African Republic), United Nations Children's Fund (UNICEF). Central African Republic Multiple Indicator Cluster Survey 2000. New York, United States: United Nations Children's Fund (UNICEF).	GHDx
Central African Republic	0	265	5565	Multiple Indicator Cluster Survey (MICS)	2006	United Nations Children's Fund (UNICEF). Central African Republic Multiple Indicator Cluster Survey 2006. New York, United States: United Nations Children's Fund (UNICEF).	GHDx
Central African Republic	0	428	6329	Multiple Indicator Cluster Survey (MICS)	2010-2011	Central African Institute of Statistics, Economic and Social Studies (ICASEES) (Central African Republic), ICF International. Central African Republic Multiple Indicator Cluster Survey 2010-2011. Fairfax, United States: ICF International, 2013.	GHDx
Chad	0	174	936	Multiple Indicator Cluster Survey (MICS)	2000	United Nations Children's Fund (UNICEF), Census Bureau (Chad), National Institute of Statistical, Economic and Demographic Studies (Chad). Chad Multiple Indicator Cluster Survey 2000. New York, United States: United Nations Children's Fund (UNICEF).	GHDx

Country	GPS-located clusters	Areally-located clusters	Number of children included	Series	Year(s)	Citation	Link
Chad	0	196	3756	Demographic and Health Survey (DHS)	2004	Macro International, Inc, National Institute of Statistical, Economic and Demographic Studies (Chad). Chad Demographic and Health Survey 2004. Calverton, United States: Macro International, Inc.	GHDx
Chad	0	459	9967	Multiple Indicator Cluster Survey (MICS)	2010	Ministry of Planning, Economy, and International Cooperation (Chad), National Institute of Statistical, Economic and Demographic Studies (Chad), United Nations Children's Fund (UNICEF). Chad Multiple Indicator Cluster Survey 2010. New York, United States: United Nations Children's Fund (UNICEF), 2014.	GHDx
Chad	624	0	13057	Demographic and Health Survey (DHS)	2014-2015	ICF International, National Institute of Statistical, Economic and Demographic Studies (Chad). Chad Demographic and Health Survey 2014-2015. Fairfax, United States: ICF International, 2016.	GHDx
Comoros	0	218	951	Multiple Indicator Cluster Survey (MICS)	2000	United Nations Development Programme (UNDP), United Nations Children's Fund (UNICEF). Comoros Multiple Indicator Cluster Survey 2000. New York, United States: United Nations Children's Fund (UNICEF).	GHDx
Comoros	242	0	2196	Demographic and Health Survey (DHS)	2012-2013	General Directorate of Statistics and Forecasting (Comoros), ICF International. Comoros Demographic and Health Survey 2012-2013. Fairfax, United States: ICF International, 2014.	GHDx
Congo	0	384	6876	Demographic and Health Survey (DHS)	2011-2012	ICF International, Ministry of Health (Congo, Rep.), National Center for Statistics and Economic Studies (Congo, Rep.). Congo Demographic and Health Survey 2011-2012. Fairfax, United States: ICF International, 2013.	GHDx
Cote d'Ivoire	0	289	1572	Multiple Indicator Cluster Survey (MICS)	2000	National School for Statistics and Economics Applied (ENSEA), United Nations Children's Fund (UNICEF), United Nations Educational, Scientific and Cultural Organization (UNESCO). Côte d'Ivoire Multiple Indicator Cluster Survey 2000. New York, United States: United Nations Children's Fund (UNICEF).	GHDx
Cote d'Ivoire	0	304	5457	Multiple Indicator Cluster Survey (MICS)	2006	United Nations Children's Fund (UNICEF), National Institute of Statistics (Côte d'Ivoire). Côte d'Ivoire Multiple Indicator Cluster Survey 2006. New York, United States: United Nations Children's Fund (UNICEF).	GHDx
Cote d'Ivoire	341	0	5252	Demographic and Health Survey (DHS)	2011-2012	ICF International, Ministry of the Fight Against AIDS (Côte d'Ivoire), National Institute of Statistics (Côte d'Ivoire). Côte d'Ivoire Demographic and Health Survey 2011-2012. Fairfax, United States: ICF International, 2013.	GHDx
Democratic Republic of the Congo	0	285	1601	Multiple Indicator Cluster Survey (MICS)	2001	Ministry of Planning and Reconstruction (Congo, DR), United Nations Children's Fund (UNICEF). Congo, DR Multiple Indicator Cluster Survey 2001. New York, United States: United Nations Children's Fund (UNICEF).	GHDx

Country	GPS-located clusters	Areally-located clusters	Number of children included	Series	Year(s)	Citation	Link
Democratic Republic of the Congo	293	0	5855	Demographic and Health Survey (DHS)	2007	Macro International, Inc, Ministry of Planning (Congo, DR). Democratic Republic of the Congo Demographic and Health Survey 2007. Calverton, United States: Macro International, Inc.	GHDx
Democratic Republic of the Congo	0	383	7462	Multiple Indicator Cluster Survey (MICS)	2010	National Statistical Institute (Congo, DR), Ministry of Planning (Congo, DR), United Nations Children's Fund (UNICEF). Congo, DR Multiple Indicator Cluster Survey 2010. New York, United States: United Nations Children's Fund (UNICEF).	GHDx
Democratic Republic of the Congo	491	1	12205	Demographic and Health Survey (DHS)	2013-2014	ICF International, Ministry of Planning and Monitoring Implementation of the Revolution of Modernity (Congo, DR), Ministry of Public Health (Congo, DR), National Institute of Statistics (Congo, DR). Democratic Republic of the Congo Demographic and Health Survey 2013-2014. Fairfax, United States: ICF International, 2014.	GHDx
Djibouti	0	212	846	Pan Arab Project for Family Health (PAPFAM)	2002	Department of Statistics and Demographic Studies (Djibouti), League of Arab States, Ministry of Health (Djibouti), Pan Arab Project for Family Health (PAPFAM). Djibouti Family Health Survey 2002.	GHDx
Djibouti	96	113	1605	Multiple Indicator Cluster Survey (MICS)	2006	Ministry of Economy, Finance, and Planning in charge of Privatization (Djibouti), Ministry of Health (Djibouti), United Nations Children's Fund (UNICEF). Djibouti Multiple Indicator Cluster Survey 2006. New York, United States: United Nations Children's Fund (UNICEF).	GHDx
Djibouti	0	198	686	Family Health Survey	2012	Department of Statistics and Demographic Studies (Djibouti), League of Arab States, Ministry of Health (Djibouti), Pan Arab Project for Family Health (PAPFAM). Djibouti Family Health Survey 2012.	GHDx
Egypt	809	0	2186	Demographic and Health Survey (DHS)	2000	Macro International, Inc, National Population Council (Egypt). Egypt Demographic and Health Survey 2000. Calverton, United States: Macro International, Inc.	GHDx
Egypt	861	0	4268	DHS Interim Demographic and Health Survey	2003	El-Zanaty and Associates, Macro International, Inc, Ministry of Health and Population (Egypt), National Population Council (Egypt). Egypt Interim Demographic and Health Survey 2003. Calverton, United States: Macro International, Inc.	GHDx
Egypt	1280	0	9873	Demographic and Health Survey (DHS)	2005	El-Zanaty and Associates, Macro International, Inc, Ministry of Health and Population (Egypt), National Population Council (Egypt). Egypt Demographic and Health Survey 2005. Calverton, United States: Macro International, Inc.	GHDx
Egypt	1208	0	7809	Demographic and Health Survey (DHS)	2008	El-Zanaty and Associates, Macro International, Inc, Ministry of Health and Population (Egypt). Egypt Demographic and Health Survey 2008. Calverton, United States: Macro International, Inc, 2009.	GHDx

Country	GPS-located clusters	Areally-located clusters	Number of children included	Series	Year(s)	Citation	Link
Egypt	0	234	3937	Multiple Indicator Cluster Survey (MICS)	2013-2014	El-Zanaty and Associates, Ministry of Health and Population (Egypt), United Nations Children's Fund (UNICEF). Egypt IPHN Rural Districts Multiple Indicator Cluster Survey 2013-2014. New York, United States: United Nations Children's Fund (UNICEF), 2016.	GHDx
Egypt	1714	0	12048	Demographic and Health Survey (DHS)	2014	El-Zanaty and Associates, ICF International, Ministry of Health and Population (Egypt). Egypt Demographic and Health Survey 2014. Fairfax, United States: ICF International, 2015.	GHDx
Equatorial Guinea	0	147	520	Multiple Indicator Cluster Survey (MICS)	2000	Ministry of Planning, Economic Development and Public Investment (Equatorial Guinea), United Nations Children's Fund (UNICEF). Equatorial Guinea Multiple Indicator Cluster Survey 2000. New York, United States: United Nations Children's Fund (UNICEF).	GHDx
Eritrea	0	368	3222	Demographic and Health Survey (DHS)	2002	Macro International, Inc, National Statistics and Evaluation Office (Eritrea). Eritrea Demographic and Health Survey 2002. Calverton, United States: Macro International, Inc.	GHDx
Ethiopia	500	0	1807	Demographic and Health Survey (DHS)	2000	Central Statistical Agency (Ethiopia), ORC Macro. Ethiopia Demographic and Health Survey 2000. Calverton, United States: ORC Macro, 2001.	GHDx
Ethiopia	527	0	6839	Demographic and Health Survey (DHS)	2005	Macro International, Inc, Population and Housing Census Commissions Office (PHCCO). Ethiopia Demographic and Health Survey 2005. Calverton, United States: Macro International, Inc.	GHDx
Ethiopia	571	0	8152	Demographic and Health Survey (DHS)	2010-2011	Central Statistical Agency (Ethiopia), ICF Macro, Ministry of Health (Ethiopia). Ethiopia Demographic and Health Survey 2010-2011. Calverton, United States: ICF Macro.	GHDx
Ethiopia	611	0	3757	Demographic and Health Survey (DHS)	2016	Central Statistical Agency (Ethiopia), ICF International. Ethiopia Demographic and Health Survey 2016. Fairfax, United States: ICF International, 2017.	GHDx
Gabon	0	226	883	Demographic and Health Survey (DHS)	2000-2001	General Directorate of Statistics and Economic Studies (Gabon), Macro International, Inc. Gabon Demographic and Health Survey 2000-2001. Calverton, United States: Macro International, Inc.	GHDx
Gabon	329	0	4293	Demographic and Health Survey (DHS)	2012	General Directorate of Statistics (Gabon), ICF International, Ministry of Economy, Employment and Sustainable Development (Gabon), Ministry of Health (Gabon). Gabon Demographic and Health Survey 2012. Fairfax, United States: ICF International, 2013.	GHDx
Ghana	407	0	2717	Demographic and Health Survey (DHS)	2003	Ghana Statistical Service, Macro International, Inc. Ghana Demographic and Health Survey 2003. Calverton, United States: Macro International, Inc.	GHDx
Ghana	0	282	1806	Multiple Indicator Cluster Survey (MICS)	2006	Ministry of Health (MOH) (Ghana), Ghana Statistical Service and United Nations Children's Fund (UNICEF). Ghana Multiple Indicator Cluster Survey 2006. New York, United States: United Nations Children's Fund (UNICEF).	GHDx

Country	GPS-located clusters	Areally-located clusters	Number of children included	Series	Year(s)	Citation	Link
Ghana	0	631	6361	Multiple Indicator Cluster Survey (MICS)	2007-2008	Ghana Statistical Service, Ministry of Health (Ghana), United Nations Children's Fund (UNICEF). Ghana District Multiple Indicator Cluster Survey 2007-2008.	GHDx
Ghana	400	0	2087	Demographic and Health Survey (DHS)	2008	Ghana Statistical Service, Macro International, Inc, Ministry of Health (Ghana). Ghana Demographic and Health Survey 2008. Calverton, United States: Macro International, Inc.	GHDx
Ghana	0	293	1686	Living Standards Measurement Study (LSMS)	2009-2010	Economic Growth Center, Yale University, Institute of Statistical, Social and Economic Research, University of Ghana. Ghana Socioeconomic Panel Survey 2009-2010. Washington DC, United States: World Bank.	GHDx
Ghana	96	0	339	Multiple Indicator Cluster Survey (MICS)	2010-2011	Institute of Statistical, Social and Economic Research, University of Ghana, United Nations Children's Fund (UNICEF). Ghana - Accra Multiple Indicator Cluster Survey 2010-2011. New York, United States: United Nations Children's Fund (UNICEF), 2014.	GHDx
Ghana	738	0	5398	Multiple Indicator Cluster Survey (MICS)	2011	Centers for Disease Control and Prevention (CDC), Ghana Statistical Service, Government of Japan, Ministry of Health (Ghana), Navrongo Health Research Centre, USAID, United Nations Children's Fund (UNICEF), United Nations Population Fund (UNFPA). Ghana Multiple Indicator Cluster Survey 2011. New York, United States: United Nations Children's Fund (UNICEF), 2013.	GHDx
Ghana	422	0	4307	Demographic and Health Survey (DHS)	2014	Ghana Health Service, Ghana Statistical Service, ICF International. Ghana Demographic and Health Survey 2014. Fairfax, United States: ICF International, 2015.	GHDx
Guinea	291	0	4132	Demographic and Health Survey (DHS)	2005	Macro International, Inc, National Statistics Directorate (Guinea). Guinea Demographic and Health Survey 2005. Calverton, United States: Macro International, Inc.	GHDx
Guinea	300	0	4975	Demographic and Health Survey (DHS)	2012	ICF Macro, Ministry of Health and Public Hygiene (Guinea), National Institute of Statistics (Guinea). Guinea Demographic and Health Survey 2012. Calverton, United States: ICF Macro, 2014.	GHDx
Guinea-Bissau	0	17	1116	Multiple Indicator Cluster Survey (MICS)	2000	Secretary State of Planning, National Institute of Statistics and Census (INEC), United Nations Children's Fund (UNICEF). Guinea-Bissau Multiple Indicator Cluster Survey 2000. New York, United States: United Nations Children's Fund (UNICEF).	GHDx
Guinea-Bissau	0	321	3871	Multiple Indicator Cluster Survey (MICS)	2006	United Nations Children's Fund (UNICEF), Government of Guinea-Bissau. Guinea-Bissau Multiple Indicator Cluster Survey 2006. New York, United States: United Nations Children's Fund (UNICEF).	GHDx
Guinea-Bissau	0	341	2915	Multiple Indicator Cluster Survey (MICS)	2014	National Statistics Institute (Guinea-Bissau), United Nations Children's Fund (UNICEF). Guinea-Bissau Multiple Cluster Indicator Survey 2014. New York, United States: United Nations Children's Fund (UNICEF), 2016.	GHDx

Country	GPS-located clusters	Areally-located clusters	Number of children included	Series	Year(s)	Citation	Link
Kenya	678	0	1530	Multiple Indicator Cluster Survey (MICS)	2000	Central Bureau of Statistics (Kenya), United Nations Children's Fund (UNICEF). Kenya Multiple Indicator Cluster Survey 2000. New York, United States: United Nations Children's Fund (UNICEF).	GHDx
Kenya	398	0	4186	Demographic and Health Survey (DHS)	2003	Centers for Disease Control and Prevention (CDC), Central Bureau of Statistics (Kenya), Macro International, Inc, Ministry of Health (Kenya), National Council for Population and Development (Kenya). Kenya Demographic and Health Survey 2003. Calverton, United States: Macro International, Inc.	GHDx
Kenya	1294	0	6086	Kenya Integrated Household Budget Survey	2005-2006	Central Bureau of Statistics (Kenya), UK Department for International Development (DFID), United States Agency for International Development (USAID), European Union (EU), Danish International Development Agency (DANIDA), World Bank (WB), United Nations Development Programme (UNDP). Kenya Integrated Household Budget Survey 2005-2006. Nairobi, Kenya: Central Bureau of Statistics (Kenya).	GHDx
Kenya	78	0	706	Multiple Indicator Cluster Survey (MICS)	2007	Kenya National Bureau of Statistics, United Nations Children's Fund (UNICEF). Kenya - North Eastern Province Multiple Indicator Cluster Survey 2007. Nairobi, Kenya: Kenya National Bureau of Statistics.	GHDx
Kenya	650	0	10589	Multiple Indicator Cluster Survey (MICS)	2008	Kenya National Bureau of Statistics, United Nations Children's Fund (UNICEF). Kenya - Eastern Province Multiple Indicator Cluster Survey 2008. Nairobi, Kenya: Kenya National Bureau of Statistics.	GHDx
Kenya	396	0	4419	Demographic and Health Survey (DHS)	2008-2009	ICF Macro, Kenya Medical Research Institute (KEMRI), Kenya National Bureau of Statistics, Ministry of Public Health and Sanitation (Kenya), National AIDS and STI Control Program (Kenya), National Aids Control Council (NACC), National Coordinating Agency for Population and Development (Kenya). Kenya Demographic and Health Survey 2008-2009. Calverton, United States: ICF Macro.	GHDx
Kenya	0	45	351	Multiple Indicator Cluster Survey (MICS)	2009	Kenya National Bureau of Statistics, United Nations Children's Fund (UNICEF). Kenya - Coast Multiple Indicator Cluster Survey 2009. New York, United States: United Nations Children's Fund (UNICEF), 2014.	GHDx
Kenya	292	0	3757	Multiple Indicator Cluster Survey (MICS)	2011	Kenya National Bureau of Statistics, United Nations Children's Fund (UNICEF). Kenya - Nyanza Province Multiple Indicator Cluster Survey 2011. Nairobi, Kenya: Kenya National Bureau of Statistics.	GHDx

Country	GPS-located clusters	Areally-located clusters	Number of children included	Series	Year(s)	Citation	Link
Kenya	50	0	663	Multiple Indicator Cluster Survey (MICS)	2013-2014	Kenya National Bureau of Statistics, Population Studies and Research Institute, University of Nairobi (Kenya), United Nations Children's Fund (UNICEF). Kenya - Bungoma County Multiple Indicator Survey 2013-2014. New York, United States: United Nations Children's Fund (UNICEF), 2015.	GHDx
Kenya	48	0	615	Multiple Indicator Cluster Survey (MICS)	2013-2014	Kenya National Bureau of Statistics, Population Studies and Research Institute, University of Nairobi (Kenya), United Nations Children's Fund (UNICEF). Kenya - Kakamega County Multiple Indicator Survey 2013-2014. New York, United States: United Nations Children's Fund (UNICEF), 2015.	GHDx
Kenya	57	0	794	Multiple Indicator Cluster Survey (MICS)	2013-2014	Kenya National Bureau of Statistics, Population Studies and Research Institute, University of Nairobi (Kenya), United Nations Children's Fund (UNICEF). Kenya - Turkana County Multiple Indicator Survey 2013-2014. New York, United States: United Nations Children's Fund (UNICEF), 2015.	GHDx
Kenya	1582	0	16025	Demographic and Health Survey (DHS)	2014	ICF International, Kenya Medical Research Institute (KEMRI), Kenya National Bureau of Statistics, Ministry of Health (Kenya), National AIDS Control Council (Kenya), National Council for Population and Development (Kenya). Kenya Demographic and Health Survey 2014. Fairfax, United States: ICF International, 2015.	GHDx
Lesotho	377	0	2409	Demographic and Health Survey (DHS)	2004-2005	Bureau of Statistics (Lesotho), Macro International, Inc, Ministry of Health and Social Welfare (Lesotho). Lesotho Demographic and Health Survey 2004-2005. Calverton, United States: Macro International, Inc.	GHDx
Lesotho	394	0	2721	Demographic and Health Survey (DHS)	2009-2010	ICF Macro, Ministry of Health and Social Welfare (Lesotho). Lesotho Demographic and Health Survey 2009-2010. Calverton, United States: ICF Macro.	GHDx
Lesotho	395	0	2205	Demographic and Health Survey (DHS)	2014	ICF International, Ministry of Health and Social Welfare (Lesotho). Lesotho Demographic and Health Survey 2014. Fairfax, United States: ICF International, 2016.	GHDx
Liberia	291	0	4007	Demographic and Health Survey (DHS)	2006-2007	Liberia Institute for Statistics and Geo-information Services (LISGIS), Macro International, Inc. Liberia Demographic and Health Survey 2006-2007. Calverton, United States: Macro International, Inc.	GHDx
Liberia	322	0	5430	Demographic and Health Survey (DHS)	2013	ICF International, Liberia Institute for Statistics and Geo-information Services (LISGIS), National AIDS and STI Control Program (NACP), Ministry of Health and Social Welfare (Liberia). Liberia Demographic and Health Survey 2013.	GHDx

Country	GPS-located clusters	Areally-located clusters	Number of children included	Series	Year(s)	Citation	Link
Madagascar	0	270	1670	Multiple Indicator Cluster Survey (MICS)	2000	National Institute of Statistics (Madagascar), United Nations Children's Fund (UNICEF). Madagascar Multiple Indicator Cluster Survey 2000. New York, United States: United Nations Children's Fund (UNICEF).	GHDx
Madagascar	0	300	3982	Demographic and Health Survey (DHS)	2003-2004	Macro International, Inc, National Institute of Statistics (Madagascar). Madagascar Demographic and Health Survey 2003-2004. Calverton, United States: Macro International, Inc.	GHDx
Madagascar	585	0	8992	Demographic and Health Survey (DHS)	2008-2009	ICF Macro, National Institute of Statistics (Madagascar). Madagascar Demographic and Health Survey 2008-2009. Calverton, United States: ICF Macro, 2010.	GHDx
Madagascar	127	0	2001	Multiple Indicator Cluster Survey (MICS)	2012	National Institute of Statistics (Madagascar), United Nations Children's Fund (UNICEF). Madagascar - South Multiple Indicator Cluster Survey 2012. New York, United States: United Nations Children's Fund (UNICEF), 2015.	GHDx
Malawi	549	0	2186	Demographic and Health Survey (DHS)	2000	Macro International, Inc, National Statistical Office of Malawi. Malawi Demographic and Health Survey 2000. Calverton, United States: Macro International, Inc.	GHDx
Malawi	520	0	7477	Demographic and Health Survey (DHS)	2004-2005	Macro International, Inc, National Statistical Office of Malawi. Malawi Demographic and Health Survey 2004-2005. Calverton, United States: Macro International, Inc.	GHDx
Malawi	0	1040	17586	Multiple Indicator Cluster Survey (MICS)	2006	United Nations Children's Fund (UNICEF), National Statistics Office (Malawi). Malawi Multiple Indicator Cluster Survey 2006. New York, United States: United Nations Children's Fund (UNICEF).	GHDx
Malawi	0	273	1329	Global Fund Household Health Coverage Survey	2007-2008	Ministry of Economic Planning and Development (Malawi), National Statistical Office of Malawi. Malawi Global Fund Household Health Coverage Survey 2007-2008.	GHDx
Malawi	827	0	14087	Demographic and Health Survey (DHS)	2010	ICF Macro, National Statistical Office of Malawi. Malawi Demographic and Health Survey 2010. Calverton, United States: ICF Macro.	GHDx
Malawi	0	1138	7676	Multiple Indicator Cluster Survey (MICS)	2013-2014	National Statistical Office of Malawi, United Nations Children's Fund (UNICEF). Malawi Multiple Indicator Cluster Survey 2013-2014. New York, United States: United Nations Children's Fund (UNICEF), 2015.	GHDx
Malawi	849	0	6403	Demographic and Health Survey (DHS)	2015-2016	ICF International, Ministry of Health (Malawi), National Statistical Office of Malawi. Malawi Demographic and Health Survey 2015-2016. Fairfax, United States: ICF International, 2017.	GHDx
Mali	405	0	9430	Demographic and Health Survey (DHS)	2006	Macro International, Inc, Ministry of Health (Mali), National Directorate of Statistics and Informatics (DNSI) (Mali). Mali Demographic and Health Survey 2006. Calverton, United States: Macro International, Inc.	GHDx

Country	GPS-located clusters	Areally-located clusters	Number of children included	Series	Year(s)	Citation	Link
Mali	0	419	11910	Multiple Indicator Cluster Survey (MICS)	2009-2010	Ministry of Health (Mali), National Institute of Statistics (INSTAT) (Mali), United Nations Children's Fund (UNICEF). Mali Multiple Indicator Cluster Survey 2009-2010. New York, United States: United Nations Children's Fund (UNICEF), 2017.	GHDx
Mali	413	0	7309	Demographic and Health Survey (DHS)	2012-2013	ICF International, INFO-STAT (Mali), Ministry of Health (Mali), National Institute of Statistics (INSTAT) (Mali), Planning and Statistics Unit, Ministry of Health (Mali). Mali Demographic and Health Survey 2012-2013. Fairfax, United States: ICF International, 2014.	GHDx
Mali	0	594	5850	Multiple Indicator Cluster Survey (MICS)	2015	Ministry of Health (Mali), Ministry of Planning (Mali), National Institute of Statistics (INSTAT) (Mali), United Nations Children's Fund (UNICEF). Mali Multiple Indicator Cluster Survey 2015. New York, United States: United Nations Children's Fund (UNICEF), 2017.	GHDx
Mauritania	0	248	860	Demographic and Health Survey (DHS)	2000-2001	Macro International, Inc, National Office of Statistics (Mauritania). Mauritania Demographic and Health Survey 2000-2001. Calverton, United States: Macro International, Inc.	GHDx
Mauritania	0	457	5051	Multiple Indicator Cluster Survey (MICS)	2007	National Office of Statistics (Mauritania), United Nations Children's Fund (UNICEF). Mauritania Multiple Indicator Cluster Survey 2007. New York, United States: United Nations Children's Fund (UNICEF).	GHDx
Mauritania	0	513	6393	Multiple Indicator Cluster Survey (MICS)	2011	National Office of Statistics (Mauritania), United Nations Children's Fund (UNICEF). Mauritania Multiple Indicator Cluster Survey 2011. New York, United States: United Nations Children's Fund (UNICEF), 2015.	GHDx
Morocco	480	0	4737	Demographic and Health Survey (DHS)	2003-2004	League of Arab States, Macro International, Inc, Ministry of Health (Morocco). Morocco Demographic and Health Survey 2003-2004. Calverton, United States: Macro International, Inc.	GHDx
Morocco	0	603	3025	Pan Arab Project for Family Health (PAPFAM)	2010-2011	Ministry of Health (Morocco), Pan Arab Project for Family Health (PAPFAM), United Nations Children's Fund (UNICEF), United Nations Population Fund (UNFPA), World Health Organization (WHO). Morocco National Survey on Population and Family Health 2010-2011.	GHDx
Mozambique	0	603	6988	Demographic and Health Survey (DHS)	2003-2004	Macro International, Inc, National Institute of Statistics (INE) (Mozambique). Mozambique Demographic and Health Survey 2003-2004. Calverton, United States: Macro International, Inc.	GHDx
Mozambique	0	6158	8134	Multiple Indicator Cluster Survey (MICS)	2008-2009	United Nations Children's Fund (UNICEF), National Statistics Institute (Mozambique). Mozambique Multiple Indicator Cluster Survey 2008-2009. New York, United States: United Nations Children's Fund (UNICEF)	GHDx

Country	GPS-located clusters	Areally-located clusters	Number of children included	Series	Year(s)	Citation	Link
Mozambique	609	0	7744	Demographic and Health Survey (DHS)	2011	ICF Macro, Manhica Health Research Center (CISM), Ministry of Health (Mozambique), National Institute of Statistics (INE) (Mozambique). Mozambique Demographic and Health Survey 2011. Calverton, United States: ICF Macro, 2013.	GHDx
Namibia	236	0	787	Demographic and Health Survey (DHS)	2000	Macro International, Inc, Ministry of Health and Social Services (Namibia), National Planning Commission (Namibia). Namibia Demographic and Health Survey 2000. Calverton, United States: Macro International, Inc.	GHDx
Namibia	485	0	3545	Demographic and Health Survey (DHS)	2006-2007	Macro International, Inc, Ministry of Health and Social Services (Namibia). Namibia Demographic and Health Survey 2006-2007. Calverton, United States: Macro International, Inc.	GHDx
Namibia	533	0	3611	Demographic and Health Survey (DHS)	2013	ICF International, Ministry of Health and Social Services (Namibia), Namibia Institute of Pathology, Namibia Statistics Agency. Namibia Demographic and Health Survey 2013. Fairfax, United States: ICF International, 2015.	GHDx
Niger	0	197	950	Multiple Indicator Cluster Survey (MICS)	2000	Government of Niger, Macro International, Inc, United Nations Children's Fund (UNICEF). Niger Multiple Indicator Cluster Survey 2000. New York, United States: United Nations Children's Fund (UNICEF).	GHDx
Niger	0	342	6263	Demographic and Health Survey (DHS)	2006	Department of Statistics and National Accounts (Niger), Macro International, Inc. Niger Demographic and Health Survey 2006. Calverton, United States: Macro International, Inc.	GHDx
Niger	0	476	8906	Demographic and Health Survey (DHS)	2012	ICF International, Ministry of Public Health (Niger), National Institute of Statistics (Niger). Niger Demographic and Health Survey 2012. Fairfax, United States: ICF International, 2014.	GHDx
Nigeria	357	0	3839	Demographic and Health Survey (DHS)	2003	National Population Commission of Nigeria, ORC Macro, UK Department for International Development (DFID), United Nations Children's Fund (UNICEF), United Nations Population Fund (UNFPA). Nigeria Demographic and Health Survey 2003. Calverton, United States: ORC Macro.	GHDx
Nigeria	0	1100	10516	Multiple Indicator Cluster Survey (MICS)	2007	United Nations Children's Fund (UNICEF), National Bureau of Statistics (Nigeria). Nigeria Multiple Indicator Cluster Survey 2007. New York, United States: United Nations Children's Fund (UNICEF).	GHDx
Nigeria	0	31	187	Nigeria General Household Survey	2007	Central Bank of Nigeria, National Bureau of Statistics (Nigeria), Nigerian Communications Commission (NCC). Nigeria General Household Survey 2007. Abuja, Nigeria: National Bureau of Statistics (Nigeria).	GHDx

Country	GPS-located clusters	Areally-located clusters	Number of children included	Series	Year(s)	Citation	Link
Nigeria	886	0	19358	Demographic and Health Survey (DHS)	2008	Macro International, Inc, National Population Commission of Nigeria. Nigeria Demographic and Health Survey 2008. Calverton, United States: Macro International, Inc, 2009.	GHDx
Nigeria	212	0	380	Living Standards Measurement Study (LSMS)	2010-2011	National Bureau of Statistics (Nigeria). Nigeria General Household Survey 2010-2011. Abuja, Nigeria: National Bureau of Statistics (Nigeria).	GHDx
Nigeria	0	1474	14780	Multiple Indicator Cluster Survey (MICS)	2011	National Bureau of Statistics (Nigeria), United Nations Children's Fund (UNICEF). Nigeria Multiple Indicator Cluster Survey 2011. New York, United States: United Nations Children's Fund (UNICEF), 2013.	GHDx
Nigeria	0	237	252	Living Standards Measurement Study (LSMS)	2012-2013	National Bureau of Statistics (Nigeria). Nigeria General Household Survey 2012-2013. Washington DC, United States: World Bank.	GHDx
Nigeria	889	0	22027	Demographic and Health Survey (DHS)	2013	ICF International, National Population Commission of Nigeria. Nigeria Demographic and Health Survey 2013. Fairfax, United States: ICF International, 2014.	GHDx
Nigeria	0	2154	9523	Multiple Indicator Cluster Survey (MICS)	2016-2017	National Agency for the Control of AIDS (Nigeria), National Bureau of Statistics (Nigeria), National Primary Health Care Development Agency (NPHCDA) (Nigeria), United Nations Children's Fund (UNICEF). Nigeria Multiple Indicator Cluster Survey with National Immunization Coverage Survey Supplement 2016-2017. New York, United States: United Nations Children's Fund (UNICEF), 2018.	GHDx
Rwanda	0	396	1292	Demographic and Health Survey (DHS)	2000	Macro International, Inc, National Office of Population (Rwanda). Rwanda Demographic and Health Survey 2000. Calverton, United States: Macro International, Inc.	GHDx
Rwanda	0	267	613	Multiple Indicator Cluster Survey (MICS)	2000	Department of Statistics (Rwanda), United Nations Children's Fund (UNICEF). Rwanda Multiple Indicator Cluster Survey 2000. New York, United States: United Nations Children's Fund (UNICEF).	GHDx
Rwanda	456	0	5889	Demographic and Health Survey (DHS)	2005	Macro International, Inc, National Institute of Statistics of Rwanda. Rwanda Demographic and Health Survey 2005. Calverton, United States: Macro International, Inc.	GHDx
Rwanda	246	0	3979	DHS Interim Demographic and Health Survey	2007-2008	Macro International, Inc, Ministry of Health (Rwanda), National Institute of Statistics of Rwanda. Rwanda Interim Demographic and Health Survey 2007-2008. Calverton, United States: Macro International, Inc.	GHDx
Rwanda	492	0	6888	Demographic and Health Survey (DHS)	2010-2011	ICF Macro, Ministry of Health (Rwanda), National Institute of Statistics of Rwanda. Rwanda Demographic and Health Survey 2010-2011. Calverton, United States: ICF Macro.	GHDx
Rwanda	492	0	5928	Demographic and Health Survey (DHS)	2014-2015	ICF International, Ministry of Health (Rwanda), National Institute of Statistics of Rwanda. Rwanda Demographic and Health Survey 2014-2015. Fairfax, United States: ICF International, 2016.	GHDx

Country	GPS-located clusters	Areally-located clusters	Number of children included	Series	Year(s)	Citation	Link
Sao Tome and Principe	0	106	418	Multiple Indicator Cluster Survey (MICS)	2000	National Institute of Statistics (Sao Tome and Principe), United Nations Children's Fund (UNICEF). Sao Tome and Principe Multiple Indicator Cluster Survey 2000. New York, United States: United Nations Children's Fund (UNICEF).	GHDx
Sao Tome and Principe	0	104	1437	Demographic and Health Survey (DHS)	2008-2009	ICF Macro, Ministry of Health (Sao Tome and Principe), National Institute of Statistics (Sao Tome and Principe). Sao Tome and Principe Demographic and Health Survey 2008-2009. Calverton, United States: ICF Macro.	GHDx
Sao Tome and Principe	0	131	790	Multiple Indicator Cluster Survey (MICS)	2014	Global Fund to Fight Aids Tuberculosis and Malaria (GFATM), ICF International, National Center for Endemic Diseases (CNE) (Sao Tome and Principe), National Institute of Statistics (Sao Tome and Principe), United Nations Children's Fund (UNICEF), United Nations Development Programme (UNDP). Sao Tome and Principe Multiple Indicator Cluster Survey 2014. New York, United States: United Nations Children's Fund (UNICEF), 2016.	GHDx
Senegal	366	0	7436	Demographic and Health Survey (DHS)	2005	Ministry of Health and Prevention (Senegal), Research Center for Human Development (Senegal). Senegal Demographic and Health Survey 2005. Calverton, United States: Macro International, Inc.	GHDx
Senegal	385	0	8867	Demographic and Health Survey (DHS)	2010-2011	Center for Research in Human Development (CRDH), Cheikh Anta Diop University, Hospital Aristide Le Dantec, ICF Macro, National Agency of Statistics and Demography (Senegal). Senegal Demographic and Health Survey 2010-2011. Calverton, United States: ICF Macro.	GHDx
Senegal	200	0	5157	Demographic and Health Survey (DHS)	2012-2013	ICF International, Ministry of Health and Social Action (Senegal), National Agency of Statistics and Demography (Senegal). Senegal Continuous Demographic and Health Survey 2012-2013. Fairfax, United States: ICF International, 2014.	GHDx
Senegal	0	199	5238	Demographic and Health Survey (DHS)	2014	Cheikh Anta Diop University, ICF International, National Agency of Statistics and Demography (Senegal). Senegal Continuous Demographic and Health Survey 2014. Fairfax, United States: ICF International, 2015.	GHDx
Senegal	214	0	5202	Demographic and Health Survey (DHS)	2015	Cheikh Anta Diop University, ICF International, National Agency of Statistics and Demography (Senegal). Senegal Continuous Demographic and Health Survey 2015. Fairfax, United States: ICF International, 2016.	GHDx
Senegal	214	0	5099	Demographic and Health Survey (DHS)	2016	ICF International, Ministry of Health and Social Action (Senegal), National Agency of Statistics and Demography (Senegal). Senegal Continuous Demographic and Health Survey 2016. Fairfax, United States: ICF International, 2017.	GHDx

Country	GPS-located clusters	Areally-located clusters	Number of children included	Series	Year(s)	Citation	Link
Sierra Leone	0	180	533	Multiple Indicator Cluster Survey (MICS)	2000	Central Statistics Office (Sierra Leone), United Nations Children's Fund (UNICEF). Sierra Leone Multiple Indicator Cluster Survey 2000. New York, United States: United Nations Children's Fund (UNICEF).	GHDx
Sierra Leone	0	330	3652	Multiple Indicator Cluster Survey (MICS)	2005	United Nations Children's Fund (UNICEF), Statistics Sierra Leone. Sierra Leone Multiple Indicator Cluster Survey 2005. New York, United States: United Nations Children's Fund (UNICEF).	GHDx
Sierra Leone	349	0	3621	Demographic and Health Survey (DHS)	2008	Macro International, Inc, Statistics Sierra Leone. Sierra Leone Demographic and Health Survey 2008. Calverton, United States: Macro International, Inc.	GHDx
Sierra Leone	0	480	6232	Multiple Indicator Cluster Survey (MICS)	2010	Statistics Sierra Leone, United Nations Children's Fund (UNICEF). Sierra Leone Multiple Indicator Cluster Survey 2010. New York, United States: United Nations Children's Fund (UNICEF).	GHDx
Sierra Leone	435	0	8038	Demographic and Health Survey (DHS)	2013	ICF International, Ministry of Health and Sanitation (Sierra Leone), Statistics Sierra Leone. Sierra Leone Demographic and Health Survey 2013. Fairfax, United States: ICF International, 2014.	GHDx
Somalia	0	248	3057	Multiple Indicator Cluster Survey (MICS)	2006	Pan Arab Project for Family Health (PAPFAM), United Nations Children's Fund (UNICEF). Somalia Multiple Indicator Cluster Survey 2006. New York, United States: United Nations Children's Fund (UNICEF).	GHDx
Somalia	259	16	2808	Multiple Indicator Cluster Survey (MICS)	2011	Ministry of National Planning and Development (Somaliland), United Nations Children's Fund (UNICEF). Somalia - Somaliland Multiple Indicator Cluster Survey 2011. New York, United States: United Nations Children's Fund (UNICEF), 2015.	GHDx
Somalia	276	0	3266	Multiple Indicator Cluster Survey (MICS)	2011	Puntland Ministry of Planning and International Cooperation (Somalia), United Nations Children's Fund (UNICEF). Somalia - Northeast Zone Multiple Indicator Cluster Survey 2011. New York, United States: United Nations Children's Fund (UNICEF), 2015.	GHDx
South Sudan	0	358	1166	Multiple Indicator Cluster Survey (MICS)	2010	Central Bureau of Statistics (Sudan), Federal Ministry of Health (Sudan), Government of Sudan, Ministry of Health (South Sudan), Southern Sudan Centre for Census, Statistics and Evaluation. Sudan - South Multiple Indicator Cluster Survey 2010. New York, United States: United Nations Children's Fund (UNICEF), 2015.	GHDx
Sudan	0	678	2988	Multiple Indicator Cluster Survey (MICS)	2000	Central Bureau of Statistics (Sudan), Federal Ministry of Health (Sudan), United Nations Children's Fund (UNICEF). Sudan Multiple Indicator Cluster Survey 2000. New York, United States: United Nations Children's Fund (UNICEF).	GHDx

Country	GPS-located clusters	Areally-located clusters	Number of children included	Series	Year(s)	Citation	Link
Sudan	0	578	2295	Multiple Indicator Cluster Survey (MICS)	2010	Central Bureau of Statistics (Sudan), Ministry of Health (South Sudan). Sudan - North Multiple Indicator Cluster Survey 2010. New York, United States: United Nations Children's Fund (UNICEF), 2015.	GHDx
Sudan	0	715	4816	Multiple Indicator Cluster Survey (MICS)	2014	Central Bureau of Statistics (Sudan), Federal Ministry of Health (Sudan), United Nations Children's Fund (UNICEF). Sudan Multiple Indicator Cluster Survey 2014. New York, United States: United Nations Children's Fund (UNICEF), 2016.	GHDx
Swaziland	0	251	697	Multiple Indicator Cluster Survey (MICS)	2000	Central Statistical Office (Swaziland), United Nations Children's Fund (UNICEF). Swaziland Multiple Indicator Cluster Survey 2000. New York, United States: United Nations Children's Fund (UNICEF).	GHDx
Swaziland	266	0	1934	Demographic and Health Survey (DHS)	2006-2007	Central Statistical Office (Swaziland), Macro International, Inc. Swaziland Demographic and Health Survey 2006-2007. Calverton, United States: Macro International, Inc.	GHDx
Swaziland	0	345	2046	Multiple Indicator Cluster Survey (MICS)	2010	Central Statistical Office (Swaziland), United Nations Children's Fund (UNICEF). Swaziland Multiple Indicator Cluster Survey 2010. New York, United States: United Nations Children's Fund (UNICEF).	GHDx
Swaziland	0	317	1087	Multiple Indicator Cluster Survey (MICS)	2014	Central Statistical Office (Swaziland), United Nations Children's Fund (UNICEF), United Nations Educational, Scientific and Cultural Organization (UNESCO), United Nations Population Fund (UNFPA). Swaziland Multiple Indicator Cluster Survey 2014. New York, United States: United Nations Children's Fund (UNICEF), 2016.	GHDx
Tanzania	0	475	6127	Demographic and Health Survey (DHS)	2004-2005	Macro International, Inc, National Bureau of Statistics (Tanzania). Tanzania Demographic and Health Survey 2004-2005. Calverton, United States: Macro International, Inc.	GHDx
Tanzania	0	837	6605	Core Welfare Indicators Questionnaire Survey (CWIQ)	2006-2007	Economic Development Initiatives (EDI), World Bank (WB). Tanzania Core Welfare Indicators Questionnaire Survey 2006-2007. Bukoba, Tanzania: Economic Development Initiatives (EDI).	GHDx
Tanzania	0	399	1927	Living Standards Measurement Study - Integrated Surveys on Agriculture (LSMS-ISA)	2008-2009	National Bureau of Statistics (Tanzania). Tanzania Living Standards Measurement Study - Integrated Survey on Agriculture 2008-2009. Dar es Salaam, Tanzania: National Bureau of Statistics (Tanzania).	GHDx
Tanzania	458	0	5719	Demographic and Health Survey (DHS)	2009-2010	ICF Macro, National Bureau of Statistics (Tanzania). Tanzania Demographic and Health Survey 2009-2010. Calverton, United States: ICF Macro.	GHDx

Country	GPS-located clusters	Areally-located clusters	Number of children included	Series	Year(s)	Citation	Link
Tanzania	604	0	4012	Demographic and Health Survey (DHS)	2015-2016	ICF International, Ministry of Health (Zanzibar), Ministry of Health, Community Development, Gender, Elderly and Children (MoHCDEC) (Tanzania), National Bureau of Statistics (Tanzania), Office of Chief Government Statistician (OCGS-Zanzibar). Tanzania Demographic and Health Survey 2015-2016. Fairfax, United States: ICF International, 2016.	GHDx
The Gambia	0	215	825	Multiple Indicator Cluster Survey (MICS)	2000	Central Statistics Department (Gambia), United Nations Children's Fund (UNICEF). Gambia Multiple Indicator Cluster Survey 2000. New York, United States: United Nations Children's Fund (UNICEF).	GHDx
The Gambia	0	329	4630	Multiple Indicator Cluster Survey (MICS)	2005-2006	Gambia Bureau of Statistics (GBOS), United Nations Children's Fund (UNICEF). Gambia Multiple Indicator Cluster Survey 2005-2006. New York, United States: United Nations Children's Fund (UNICEF).	GHDx
The Gambia	0	281	5791	Demographic and Health Survey (DHS)	2013	Gambia Bureau of Statistics (GBOS), ICF International, Ministry of Health and Social Welfare (Gambia). Gambia Demographic and Health Survey 2013. Fairfax, United States: ICF International, 2015.	GHDx
Togo	0	300	2606	Multiple Indicator Cluster Survey (MICS)	2006	Directorate General of Statistics and National Accounting (Togo), United Nations Children's Fund (UNICEF). Togo Multiple Indicator Cluster Survey 2006. New York, United States: United Nations Children's Fund (UNICEF).	GHDx
Togo	0	446	3346	Multiple Indicator Cluster Survey (MICS)	2010	Directorate General of Statistics and National Accounting (Togo), United Nations Children's Fund (UNICEF). Togo Multiple Indicator Cluster Survey 2010. New York, United States: United Nations Children's Fund (UNICEF).	GHDx
Togo	330	0	5176	Demographic and Health Survey (DHS)	2013-2014	Directorate General of Statistics and National Accounts (Togo), ICF International, Ministry of Health (Togo). Togo Demographic and Health Survey 2013-2014. Fairfax, United States: ICF International, 2015.	GHDx
Tunisia	0	466	2262	Multiple Indicator Cluster Survey (MICS)	2011-2012	Ministry of Regional Development and Planning (Tunisia), National Institute of Statistics (Tunisia), United Nations Children's Fund (UNICEF). Tunisia Multiple Indicator Cluster Survey 2011-2012. New York, United States: United Nations Children's Fund (UNICEF), 2014.	GHDx
Uganda	259	26	1389	Demographic and Health Survey (DHS)	2000-2001	Macro International, Inc, Uganda Bureau of Statistics. Uganda Demographic and Health Survey 2000-2001. Calverton, United States: Macro International, Inc.	GHDx
Uganda	336	0	5442	Demographic and Health Survey (DHS)	2006	Macro International, Inc, Uganda Bureau of Statistics. Uganda Demographic and Health Survey 2006. Calverton, United States: Macro International, Inc.	GHDx
Uganda	400	0	5670	Demographic and Health Survey (DHS)	2011	ICF Macro, Uganda Bureau of Statistics. Uganda Demographic and Health Survey 2011. Calverton, United States: ICF Macro.	GHDx

Country	GPS-located clusters	Areally-located clusters	Number of children included	Series	Year(s)	Citation	Link
Zambia	0	318	2446	Demographic and Health Survey (DHS)	2001-2002	Central Board of Health (Zambia), Central Statistical Office (Zambia), Macro International, Inc. Zambia Demographic and Health Survey 2001-2002. Calverton, United States: Macro International, Inc.	GHDx
Zambia	0	520	4678	Zambia Living Conditions Monitoring Survey	2002-2003	Central Statistical Office (Zambia). Zambia Living Conditions Monitoring Survey 2002-2003. Lusaka, Zambia: Central Statistical Office (Zambia).	GHDx
Zambia	319	0	4576	Demographic and Health Survey (DHS)	2007	Central Statistical Office (Zambia), Macro International, Inc. Zambia Demographic and Health Survey 2007. Calverton, United States: Macro International, Inc.	GHDx
Zambia	0	186	1817	Global Fund Household Health Coverage Survey	2008	Central Statistical Office (Zambia). Zambia Global Fund Household Health Coverage Survey 2008. Lusaka, Zambia: Central Statistical Office (Zambia).	GHDx
Zambia	719	0	9882	Demographic and Health Survey (DHS)	2013-2014	Central Statistical Office (Zambia), ICF International, Ministry of Health (Zambia), Tropical Diseases Research Centre, University Teaching Hospital (Zambia), University of Zambia. Zambia Demographic and Health Survey 2013-2014. Fairfax, United States: ICF International, 2015.	GHDx
Zimbabwe	396	0	3743	Demographic and Health Survey (DHS)	2005-2006	Central Statistical Office (Zimbabwe), Macro International, Inc. Zimbabwe Demographic and Health Survey 2005-2006. Calverton, United States: Macro International, Inc.	GHDx
Zimbabwe	0	500	5663	Multiple Indicator Cluster Survey (MICS)	2009	Central Statistical Office (Zimbabwe). Zimbabwe Multiple Indicator Monitoring Survey 2009. New York, United States: United Nations Children's Fund (UNICEF).	GHDx
Zimbabwe	392	0	3131	Demographic and Health Survey (DHS)	2010-2011	ICF Macro, Zimbabwe National Statistics Agency. Zimbabwe Demographic and Health Survey 2010-2011. Calverton, United States: ICF Macro, 2012.	GHDx
Zimbabwe	0	682	7805	Multiple Indicator Cluster Survey (MICS)	2014	United Nations Children's Fund (UNICEF), Zimbabwe National Statistics Agency. Zimbabwe Multiple Indicator Cluster Survey 2014. New York, United States: United Nations Children's Fund (UNICEF), 2015.	GHDx
Zimbabwe	398	0	2311	Demographic and Health Survey (DHS)	2015	ICF International, National Microbiology Reference Laboratory, Harare Central Hospital (NMRL) (Zimbabwe), Zimbabwe National Statistics Agency. Zimbabwe Demographic and Health Survey 2015. Fairfax, United States: ICF International, 2016.	GHDx

Supplementary Table 2: Data excluded from both the geostatistical model and GBD estimates

Country	Series	Year(s)	Citation	Rationale for exclusion
Angola	Core Welfare Indicators Questionnaire Survey (CWIQ)	2011	Ministry of Planning and Territorial Development (Angola), National Institute of Statistics (Angola). Angola Core Welfare Indicators Questionnaire Survey 2011. Luanda, Angola: National Institute of Statistics (Angola).	Estimates considered implausible. Survey estimates are systematically high compared to admin estimates and estimates from other established survey series (2015-2016 DHS and the 2008-2009 Angola Integrated Inquiry into People's Well-Being)
Congo	Demographic and Health Surveys (DHS)	2005	Macro International, Inc, National Center for Statistics and Economic Studies (Congo, Rep.). Congo Demographic and Health Survey 2005. Calverton, United States: Macro International, Inc.	Estimates considered implausible. Survey estimates do not follow a reasonable age structure. Older children have significantly higher coverage than reported in either administrative data or other survey series.
Ghana	Living Standards Measurement Survey (LSMS)	2012-2013	Ghana Statistical Service, World Bank. Ghana Living Standards Measurement Survey 2012-2013. Accra, Ghana: Ghana Statistical Service	Estimates considered implausible. Survey estimates are systematically lower for DPT3 coverage than reported in other survey series (2011 MICS, 2008 DHS, 2014 DHS).
Nigeria	Core Welfare Indicators Questionnaire Survey (CWIQ)	2006	National Bureau of Statistics (Nigeria). Nigeria Core Welfare Indicators Questionnaire Survey 2006. Abuja, Nigeria: National Bureau of Statistics (Nigeria).	Survey estimates are systematically high compared to admin estimates and estimates from other established survey series(2008 DHS, 2007 MICS)
Nigeria	Living Standards Measurement Survey (LSMS)	2008-2010	National Bureau of Statistics (Nigeria). Nigeria Living Standards Survey 2008-2010. Abuja, Nigeria: National Bureau of Statistics (Nigeria).	Survey estimates are systematically high compared to admin estimates and estimates from other established survey series (2008 DHS, 2007 MICS)
Uganda	National Service Delivery Survey	2004	Ministry of Public Service (Uganda), Uganda Bureau of Statistics. Uganda National Service Delivery Survey 2004. OpenMicroData.	Estimates considered implausible. Survey estimates are inconsistent with both admin estimates and estimates from other established survey series (2006 DHS)
Uganda	National Service Delivery Survey	2008	Ministry of Public Service (Uganda), Uganda Bureau of Statistics. Uganda National Service Delivery Survey 2008.	Estimates considered implausible. Survey estimates are inconsistent with both admin estimates and estimates from other established survey series (2006 DHS)

Supplementary Table 3: Data excluded from GBD estimates but included in geostatistical model

Country	Series	Year(s)	Citation	Rationale for exclusion from GBD model
Madagascar	Multiple Indicator Cluster Survey (MICS)	2012	National Institute of Statistics (Madagascar), United Nations Children's Fund (UNICEF). Madagascar - South Multiple Indicator Cluster Survey 2012. New York, United States: United Nations Children's Fund (UNICEF), 2015.	Not nationally representative. Only sampled the south of Madagascar.
Kenya	Multiple Indicator Cluster Survey (MICS)	2007	Kenya National Bureau of Statistics, United Nations Children's Fund (UNICEF). Kenya - North Eastern Province Multiple Indicator Cluster Survey 2007. Nairobi, Kenya: Kenya National Bureau of Statistics.	Not nationally representative. Only sampled North Eastern province.

3.1 Data sources excluded from model

In order to evaluate for the possibility of systematic survey bias, national-level time series of survey estimates of DPT coverage were reviewed for each country along with national-level coverage estimates from WHO, GBD, and the geospatial models. When a survey's estimates appeared implausible in comparison with other existing survey-based data sources, the survey methodology was inspected to evaluate for any apparent differences in case definitions, data collection, or other methodological explanations for the observed discrepancy. Data from surveys with implausible results were also mapped spatially and reviewed in order to identify surveys that were non-nationally representative (and therefore excluded from GBD models) but could provide useful spatial information for the geospatial models.

Supplementary Table 2 shows data sources that were identified during the data search process and met general inclusion criteria but were specifically excluded from the model due to reported vaccine deemed implausible based on review of corroborating sources or with known methodologic flaws. For each survey, reasons for exclusion are specified in the table.

3.2 Data preparation

Both vaccine card and maternal recall data were extracted from all sources; vaccine card data was preferentially used and maternal recall only if no vaccine card data were available. Within each source, data with missing spatial identifiers, age information, or survey weights were excluded, as were any areal units that consisted of a single individual (as survey-adjusted means could not be calculated for such units).

Supplementary Figure 1 shows the survey data identification and extraction process as well as data loss during data cleaning steps.

Individual ages were determined using age in months at the time of survey when available and age in years otherwise. Individuals were then assigned to birth cohorts based on age at the time of the survey observation (12-23 months, 24-35 months, 36-47 months, and 48-59 months). The data corresponding to each birth cohort were then included in the geospatial modelling process in the year during which the birth cohort was 12-23 months of age. In other words, for a survey conducted in year *Y*, data from 12-23 month olds were included in year *Y*, data from 24-35 month olds in year *Y-1*, and data from other cohorts included analogously. Including data from children older than 23 months of age assumes negligible catch-up vaccination and does not capture effects of migration or differential mortality by vaccination, but broadens the geographical representativeness of the model by including of data from locations where no children 12-23 months of age were sampled, but data from older children was available.

Given the limitations of this approach, we considered whether an adjustment could be made to data from children ≥ 23 months to account for some or all of these effects. Supplementary Figure 2 shows a line plot of age-cohort-specific DPT3 coverage, by survey, in comparison to the observed coverage in the baseline age cohort. While there is substantial variation in the age-cohort-specific coverage pattern by survey, there was no consistent evidence of either a positive or a negative trend in coverage across age cohorts. Given these results, we elected not to apply a bias correction to vaccine coverage data from age cohorts > 24 months. Further methods development will be required to better incorporate age-specific coverage effects into a temporally-varying model-based geostatistical framework.

Supplementary Figure 1: Flow diagram of survey identification and data loss during data cleaning.

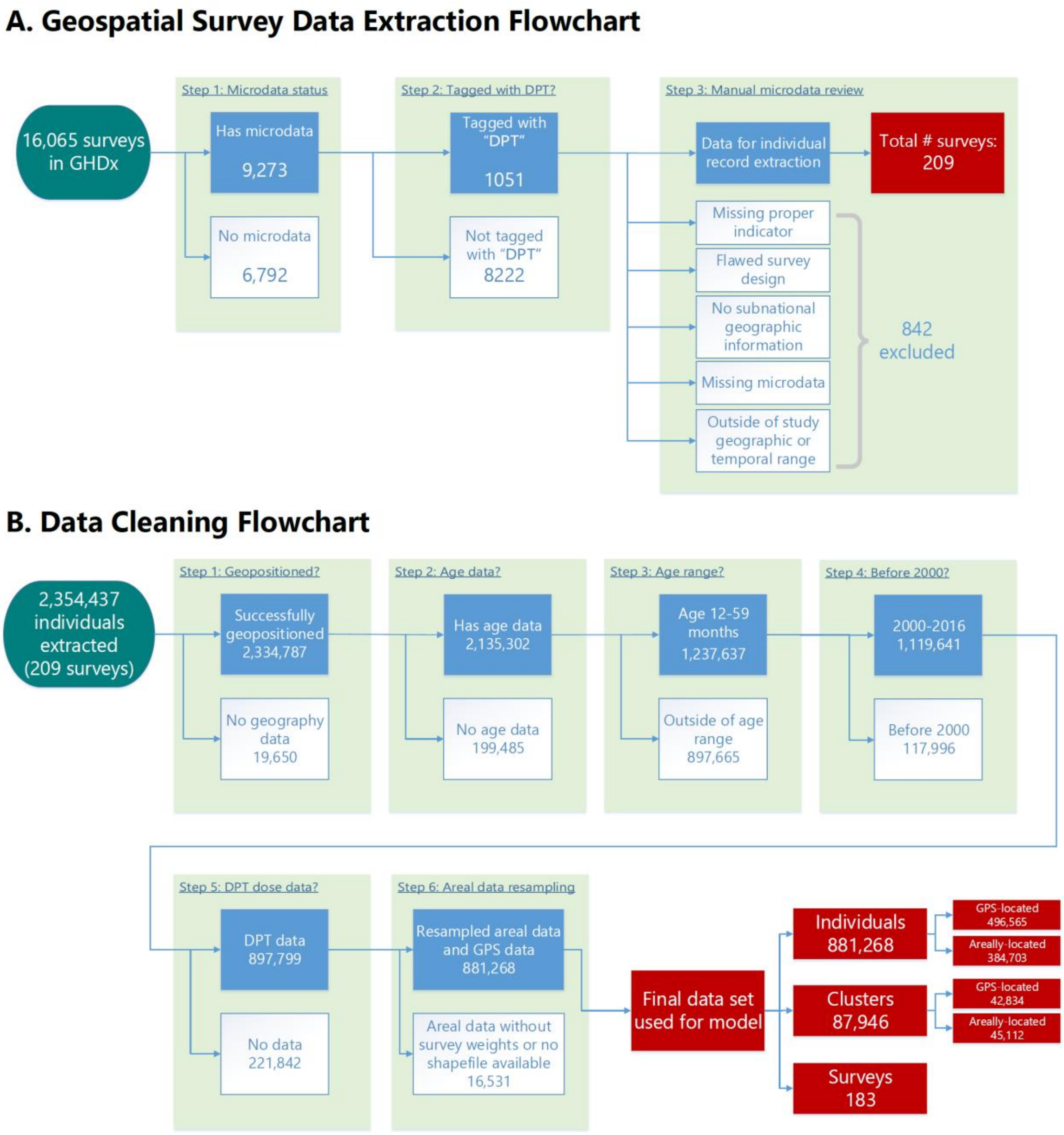

Supplementary Figure 2: Weighted DPT3 coverage, by age cohort and survey.
Each line represents weighted age-cohort-specific DPT3 coverage for one survey. The lines trace the difference in DPT3 coverage (y-axis) between a given age cohort (x-axis) and the baseline age cohort (12-23 month olds). The line of constant coverage across age cohorts is represented as a black dotted line, and the red line represents the sample-size weighted average of coverage differences across all surveys.

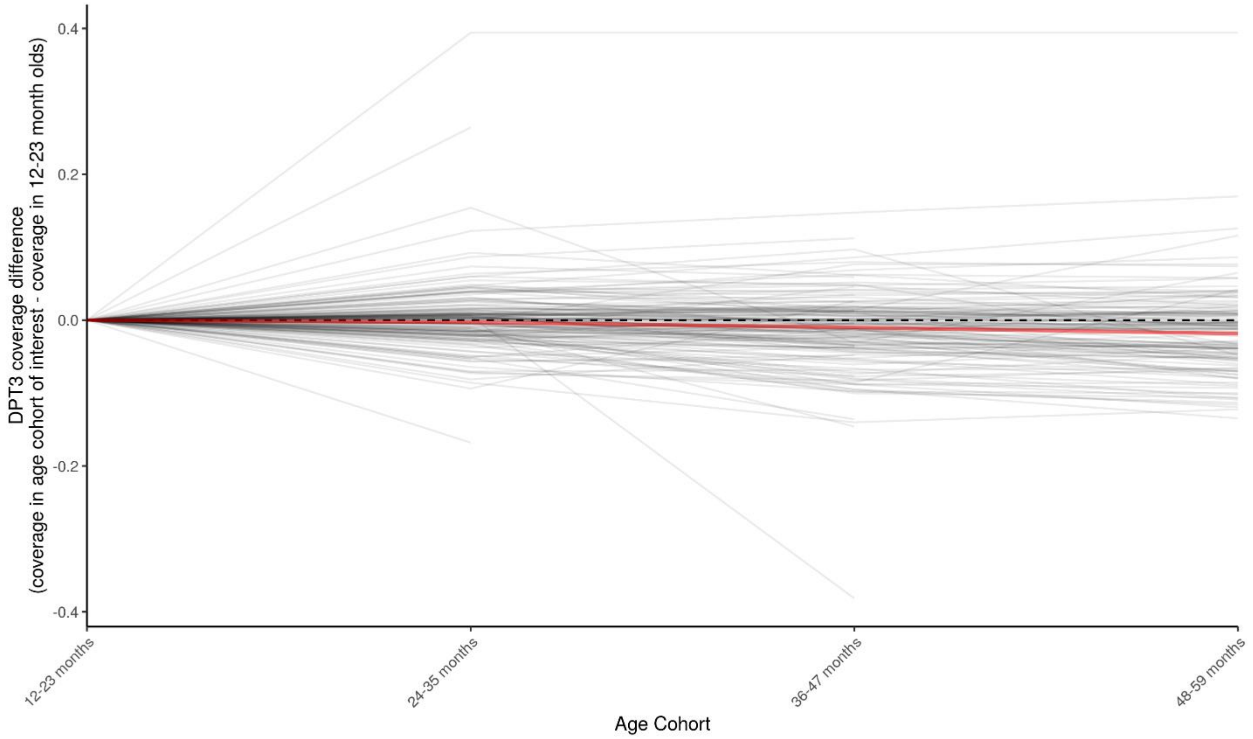

3.3 Geographic positioning of clusters and spatial integration of areal data

Individual-level data were processed as described above, then summarized to smallest geographic resolution available. Each individual survey record was matched by primary sampling unit to the smallest geographic area available. When geographic coordinates were not available, the names of the smallest available representative administrative units were extracted. If these units were smaller than 5x5km, each unit was assigned a latitude and longitude from the centroid of the representative area. Otherwise, these units were then matched to administrative shapefiles that were either obtained from the survey itself or taken from the Global Administrative Unit Layers database[3] or the Database of Global Administrative Areas.[4]

In the model-based geostatistical framework used in this study, all input data must be located at the latitude/longitude level. For areal data with no precise latitude and longitude available but that could be spatially matched to an administrative unit or other subnational geography, a previously-described spatial integration approach[5–7] was used to create weighted candidate points from each set of areal data while accounting for survey weights.

Briefly, for each areal unit within a survey for which precise geographic coordinates were not available, an estimate of mean vaccine coverage was calculated from the individual-level data using survey weights and design effects using the *survey* package in R.[8] This mean estimate was matched with a spatial polygon representation of the areal unit from which the data were obtained, and 10,000 points were randomly sampled from within that polygon with the probability of selection proportional to the total population at that location in space and time according to the WorldPop total population raster.[9,10] From these randomly sampled points in space, k-means clustering was used to generate a set of integration points (1 per 1,000 5x5 km pixels within the polygon) that were used as input data for

the geospatial model. Each integration point was assigned the mean vaccine coverage estimate for the areal unit (generated using survey weights as above) and assigned a weight proportional to the number of randomly sampled points that were included in the k-means cluster such that all weights sum to 1.

The net effect of this process is to produce a set of candidate observations, where more populated locations within the areal unit are more likely to be selected, and points selected in higher-population-density areas are more heavily weighted. The total weighted sample size of this set of candidate points is identical to the sample size of the original individual-level data used in the resampling process. As an example of this process,

Supplementary Figure 3 shows an example of the survey-weighted mean estimates generated for each area, the population raster, the 10,000 points randomly sampled proportional to population, and the final k-means-clustered, weighted integration points that represent candidate observations to be used as model input data.

Supplementary Figure 3: Spatial resampling of areal data

Survey-weighted mean estimates of DPT3 coverage for each areal unit represented in a given country, survey, and year (A); population raster from WorldPop used for the spatial resampling process (B); 10,000 points sampled randomly proportionally to population (C); k-means clustered, weighed integration points to be used as candidate observations in the geospatial model (D).

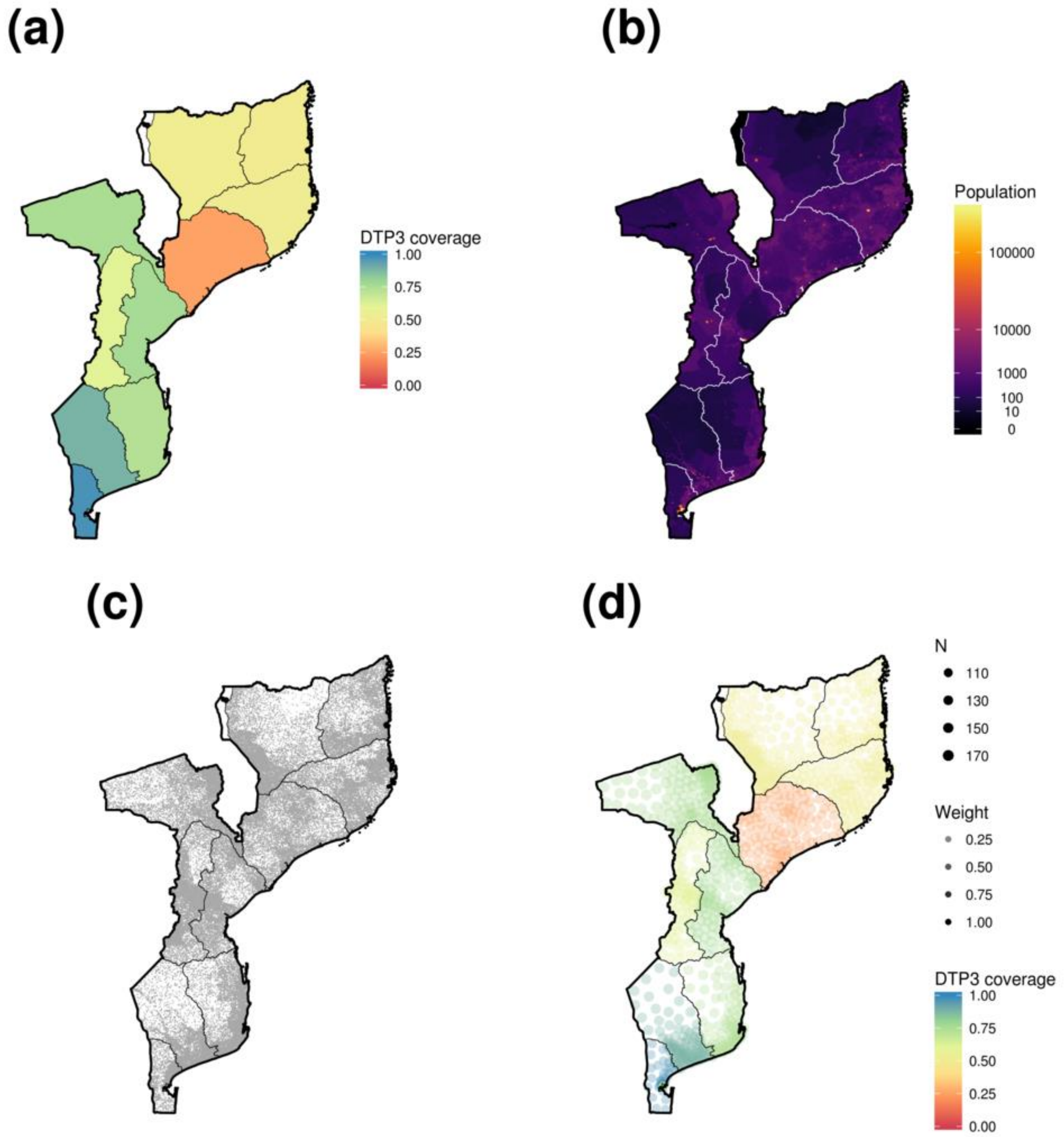

4.0 Covariates

A suite of socioeconomic and environmental covariates was assembled in order to enhance the ability of the model to predict vaccine coverage. Supplementary Table 4 details the spatial and temporal resolution of each included covariate and provides references. Maps of high-resolution spatial covariates can be found in Supplementary Figure 4.

Supplementary Table 4: Covariates used in mapping of vaccine coverage.

Covariates are listed as "GBD" obtained at the national level using estimates from the Global Burden of Disease Study 2016.[11]

Covariate	Spatial resolution	Temporal resolution	Source	Reference
Antenatal care coverage (4 visits)	National	Annual	GBD	GBD covariate: "Proportion of pregnant woman receiving 4 or more antenatal care visits including 1 or more from a skilled provider"
Average daily maximum temperature	5x5 km	Annual	CRUTS	Harris, I., Jones, P. D., Osborn, T. J. & Lister, D. H. Updated high-resolution grids of monthly climatic observations – the CRU TS3.10 dataset. *InT. J. Climatol*. **34**, 623–642 (2014).<br>University of East Anglia. Climatic Research Unit TS v. 3.24 dataset. Available at: https://crudata.uea.ac.uk/cru/data/hrg/cru_ts_3.24.01/. (Accessed: 24th July 2017).
Daytime LST	5x5 km	Annual	MODIS	USGS & NASA. Land surface temperature and emissivity 8-day L3 global 1km MOD11A2 dataset. Available at: https://lpdaac.usgs.gov/dataset_discovery/modis/modis_products_table/mod11a2. (Accessed: 24th July 2017)<br>Wan, Z. MODIS Land-Surface Temperature Algorithm Theoretical Basis Document (LST ATBD).<br>Weiss, D. J. et al. An effective approach for gap-filling continental scale remotely sensed time-series. *Isprs J. Photogramm. Remote Sens*. **98**, 106–118 (2014).
Distance to rivers and lakes ≥ 50 km	5x5 km	Static	Natural Earth Data (derived)	Natural Earth. Rivers and lake centerlines dataset. Available at: http://www.naturalearthdata.com/downloads/10m-physical-vectors/10m-rivers-lake-centerlines/. (Accessed: 24th July 2017)
Diurnal difference in LST	5x5 km	Annual	MODIS	USGS & NASA. Land surface temperature and emissivity 8-day L3 global 1km MOD11A2 dataset. Available at: https://lpdaac.usgs.gov/dataset_discovery/modis/modis_products_table/mod11a2. (Accessed: 24th July 2017)<br>Wan, Z. MODIS Land-Surface Temperature Algorithm Theoretical Basis Document (LST ATBD).<br>Weiss, D. J. et al. An effective approach for gap-filling continental scale remotely sensed time-series. *Isprs J. Photogramm. Remote Sens*. **98**, 106–118 (2014).
Diurnal temperature range	5x5 km	Annual	CRUTS	Harris, I., Jones, P. D., Osborn, T. J. & Lister, D. H. Updated high-resolution grids of monthly climatic observations – the CRU TS3.10 dataset. *InT. J. Climatol*. **34**, 623–642 (2014).<br>University of East Anglia. Climatic Research Unit TS v. 3.24 dataset. Available at: https://crudata.uea.ac.uk/cru/data/hrg/cru_ts_3.24.01/. (Accessed: 24th July 2017).
Elevation	5x5 km	Static	National Geophysical Data Center, NOAA	National Geophysical Data Center, National Oceanic and Atmosphere Administration. Global Land One-kilometre Base Elevation. Boulder, United States: National Geophysical Data Center, National Oceanic and Atmosphere Administration, 1999.
Enhanced Vegetation Index (EVI)	5x5 km	Annual	MODIS	Huete, A., Justice, C. & van Leeuwen, W. MODIS vegetation index (MOD 13) algorithm theoretical basis document. (1999).<br>USGS & NASA. Vegetation indices 16-Day L3 global 500m MOD13A1 dataset. Available at: https://lpdaac.usgs.gov/dataset_discovery/modis/modis_products_table/mod13a1. (Accessed: 25th July 2017)<br>Weiss, D. J. et al. An effective approach for gap-filling continental scale remotely sensed time-series. *Isprs J. Photogramm. Remote Sens*. **98**, 106–118 (2014).
Fertility	5x5 km	Annual	WorldPop (derived)	Lloyd, C. T., Sorichetta, A. & Tatem, A. J. High resolution global gridded data for use in population studies. *Sci. Data* **4**, sdata20171 (2017).<br>World Pop. Get data. Available at: http://www.worldpop.org.uk/data/get_data/. (Accessed: 25th July 2017)
Global Protected Areas	5x5 km	Annual	World Database of Protected Areas (WDPA)	UNEP-WCMC and IUCN. Protected Planet: The World Database on Protected Areas (WDPA) Cambridge, UK: UNEP-WCMC and IUCN, 2018.Available at: https://www.protectedplanet.net (Accessed: 23rd January 2018).
Growing season length	5x5 km	Static	FAO	FAO. GAEZ - Global Agro-Ecological Zones data portal. Available at: http://www.fao.org/nr/gaez/about-data-portal/en/. (Accessed: 25th July 2017)<br>FAO. GAEZ - Global Agro-Ecological Zones users guide. (2012).
Irrigation	5x5 km	Static	University of Frankfurt	Goethe-Universität. Generation of a digital global map of irrigation areas. Available at: https://www.uni-frankfurt.de/45218039/Global_Irrigation_Map. (Accessed: 25th July 2017)
Lag-distributed income per capita	National	Annual	GBD	GBD covariate: "Lag distributed income per capita (I$): gross domestic product per capita that has been smoothed over the preceding 10 years"

Covariate	Spatial resolution	Temporal resolution	Source	Reference
Malaria incidence	5x5 km	Annual	Malaria Atlas Project	Bhatt, S. et al. The effect of malaria control on *Plasmodium falciparum* in Africa between 2000 and 2015. *Nature* **526**, 207–211 (2015).
Maternal Education	5x5 km	Annual	Internally Modelled – IHME LBD	Graetz N, Friedman J, Osgood-Zimmerman A, et al. Mapping local variation in educational attainment across Africa. *Nature*. 2018;555(7694):48-53.
Mortality rate due to war and terrorism in the past 10 years	National	Annual	GBD	GBD covariate: "Mean mortality rate in the previous ten years due to war and terrorism, measured from year's end, current year included"
Nighttime lights	5x5 km	Annual	NOAA DMSP	Savory et al. Intercalibration and Gaussian Process Modelling of Nighttime Lights Imagery for Measuring Urbanization Trends in Africa 2000–2013. *Remote Sens*. **9**, (2017).
Normalized Difference Vegetation Index (NDVI)	5x5 km	Annual	AVHRR	NASA & NOAA. Advanced Very High Resolution Radiometer (AVHRR) Normalized Difference Vegetation Index (NDVI) dataset. Available at: https://nex.nasa.gov/nex/projects/1349/. (Accessed: 25th July 2017)
Population	5x5 km	Annual	WorldPop	Lloyd, C. T., Sorichetta, A. & Tatem, A. J. High resolution global gridded data for use in population studies. *Sci. Data* **4**, sdata20171 (2017).<br>World Pop. Get data. Available at: http://www.worldpop.org.uk/data/get_data/. (Accessed: 25th July 2017)
Potential Evapotranspiration (PET)	5x5 km	Annual	CRUTS	Harris, I., Jones, P. D., Osborn, T. J. & Lister, D. H. Updated high-resolution grids of monthly climatic observations – the CRU TS3.10 dataset. *InT. J. Climatol*. **34**, 623–642 (2014).<br>University of East Anglia. Climatic Research Unit TS v. 3.24 dataset. Available at: https://crudata.uea.ac.uk/cru/data/hrg/cru_ts_3.24.01/. (Accessed: 24th July 2017).
Precipitation	5x5 km	Annual	MSWEP	Beck, H.E., A.I.J.M. van Dijk, V. Levizzani, J. Schellekens, D.G. Miralles, B. Martens, A. de Roo: MSWEP: 3-hourly 0.25 global gridded precipitation (1979-2015) by merging gauge, satellite, and reanalysis data, Hydrology and Earth System Sciences, 21(1), 589-615, 2017.
Tassled cap brightness	5x5 km	Annual	MODIS	USGS & NASA. Nadir BRDF- Adjusted Reflectance Reflectance 16-Day L3 Global 1km dataset. Available at: https://lpdaac.usgs.gov/dataset_discovery/modis/modis_products_table/mcd43b4. (Accessed: 25th July 2017)<br>Strahler, A. H. & Muller, J.-P. MODIS BRDF/Albedo product: algorithm theoretical basis document version 5.0. (1999).<br>Weiss, D. J. et al. An effective approach for gap-filling continental scale remotely sensed time-series. *Isprs J. Photogramm. Remote Sens*. **98**, 106–118 (2014).
Tassled cap wetness	5x5 km	Annual	MODIS	USGS & NASA. Nadir BRDF- Adjusted Reflectance Reflectance 16-Day L3 Global 1km dataset. Available at: https://lpdaac.usgs.gov/dataset_discovery/modis/modis_products_table/mcd43b4. (Accessed: 25th July 2017)<br>Strahler, A. H. & Muller, J.-P. MODIS BRDF/Albedo product: algorithm theoretical basis document version 5.0. (1999).
Travel time to nearest settlement >50,000 inhabitants	5x5 km	Static	Malaria Atlas Project, Oxford Big Data Institute, Li Ka Shing Centre for Health Information and Discovery, University of Oxford	D.J. Weiss *et al*. A global map of travel time to cities to assess inequalities in accessibility in 2015. Nature doi:10.1038/nature25181 (preprint, 2018)
Urbanicity	5x5 km	Annual	European Commission/GHS	Pesaresi, M. et al. Operating procedure for the production of the Global Human Settlement Layer from Landsat data of the epochs 1975, 1990, 2000, and 2014. (Publications Office of the European Union, 2016).
Wet day frequency	5x5 km	Annual	CRUTS	Harris, I., Jones, P. D., Osborn, T. J. & Lister, D. H. Updated high-resolution grids of monthly climatic observations – the CRU TS3.10 dataset. *InT. J. Climatol*. **34**, 623–642 (2014).<br>University of East Anglia. Climatic Research Unit TS v. 3.24 dataset. Available at: https://crudata.uea.ac.uk/cru/data/hrg/cru_ts_3.24.01/. (Accessed: 24th July 2017).

Supplementary Figure 4: Maps of included high-resolution covariates.
Each time-varying covariate is represented by a map for the year 2016.

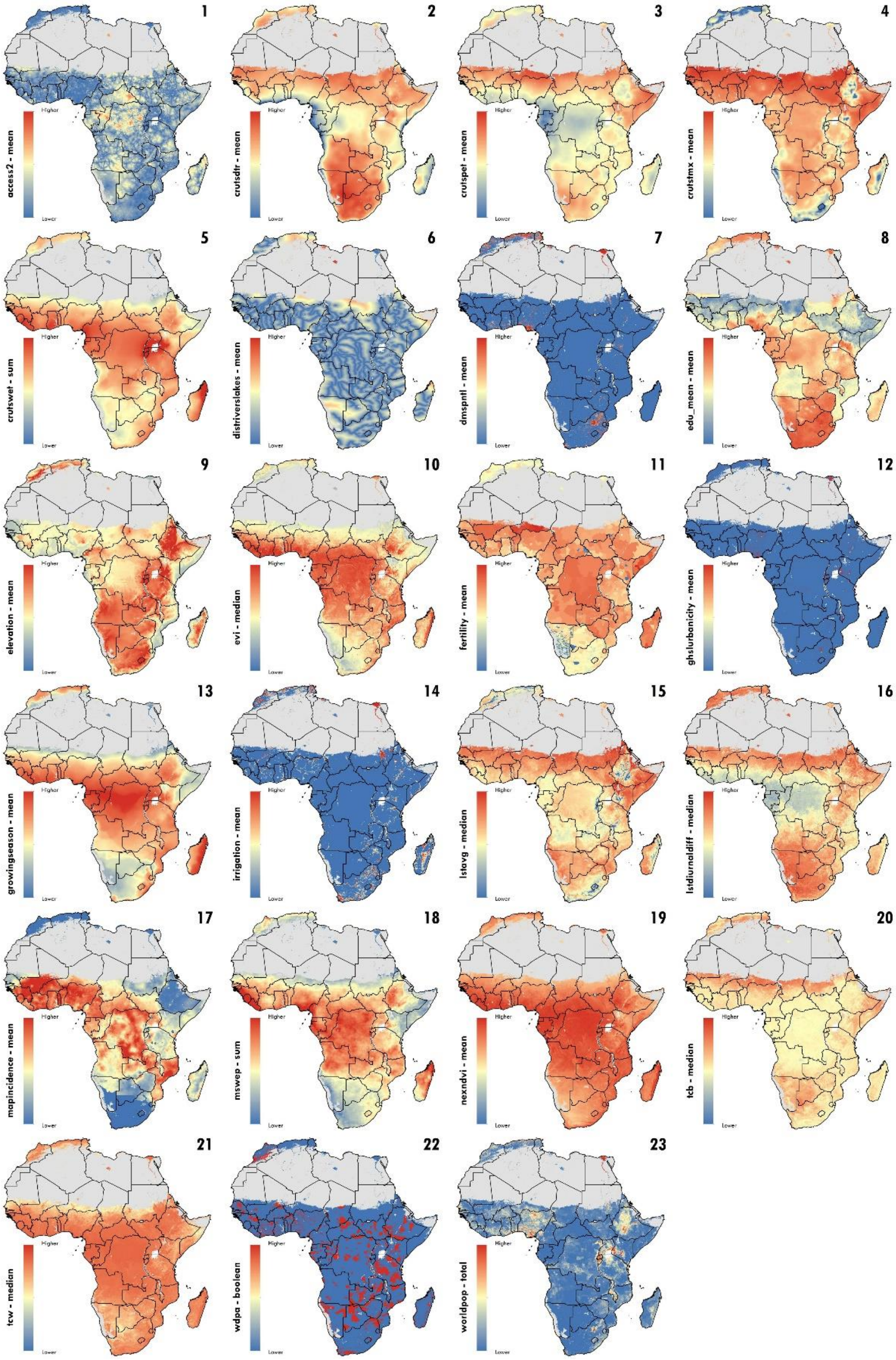

5.0 Geostatistical Model

5.1 Overview of modelling process

A schematic overview of the modelling process can be found in Supplementary Figure 5. Briefly, a point-level vaccine coverage dataset was created and a suite of spatial covariates assembled as described above. For each modelled quantity, five regional sub-models were run. For each regional sub-model, an ensemble covariate modelling method (stacked generalization[12]) was used to capture complex interactions and non-linear relationships between covariates and vaccine coverage. The outputs of this ensemble modelling approach are covariate-based predictions of vaccine coverage, which were then themselves used as covariates in the geospatial (model-based geostatistical) model. The geospatial model leverages covariates and spatiotemporal patterns in the data to produce 1,000 candidate maps (samples or draws) each of which represents a possible representation of vaccine coverage at all modelled spaces and times.

In order to estimate DPT1 coverage, DPT3 coverage, and dropout in an internally consistent manner, a conditional ordinal regression framework was employed. In this framework, the above process was repeated three times for different measures of conditional vaccine coverage, which were then combined arithmetically to produce estimates of the distribution of vaccine coverage at that draw level (see section 5.4.5 for details). (In other words, for each modelled location in space and time, this framework generates 1,000 possible distributions of the proportion of children receiving 0, 1, 2, or 3+ doses of DPT). From these estimates, DPT1 coverage, DPT3 coverage, and dropout were similarly calculated at the draw level.

These estimates were then calibrated to national-level estimates of DPT coverage generated using methodology from the Global Burden of Diseases, Injuries, and Risk Factors Study (GBD) 2017. These national-level estimates additionally leverage bias-corrected administrative data to better capture overall time trends in places where survey-based data is scant. Finally, these estimates were summarized as mean estimates of vaccine coverage with associated uncertainty at the 5x5 km and first and second administrative levels. The probability of meeting GVAP targets in the year 2016 was calculated for countries and second-order administrative units as the percentage of draws where the targets were met. To analyse changes in DPT coverage over time across Africa at the second administrative level, the proportion of second-level administrative units with increasing coverage from 2000 to 2016 was calculated at the draw level. This calculation results in a posterior distribution of the proportion of second-level administrative units experiencing an increase in coverage, which was then summarized as a mean and 95% uncertainty interval. The same calculations were repeated for decreases in coverage at the second administrative level.

Supplementary Figure 5: Overview of modelling process

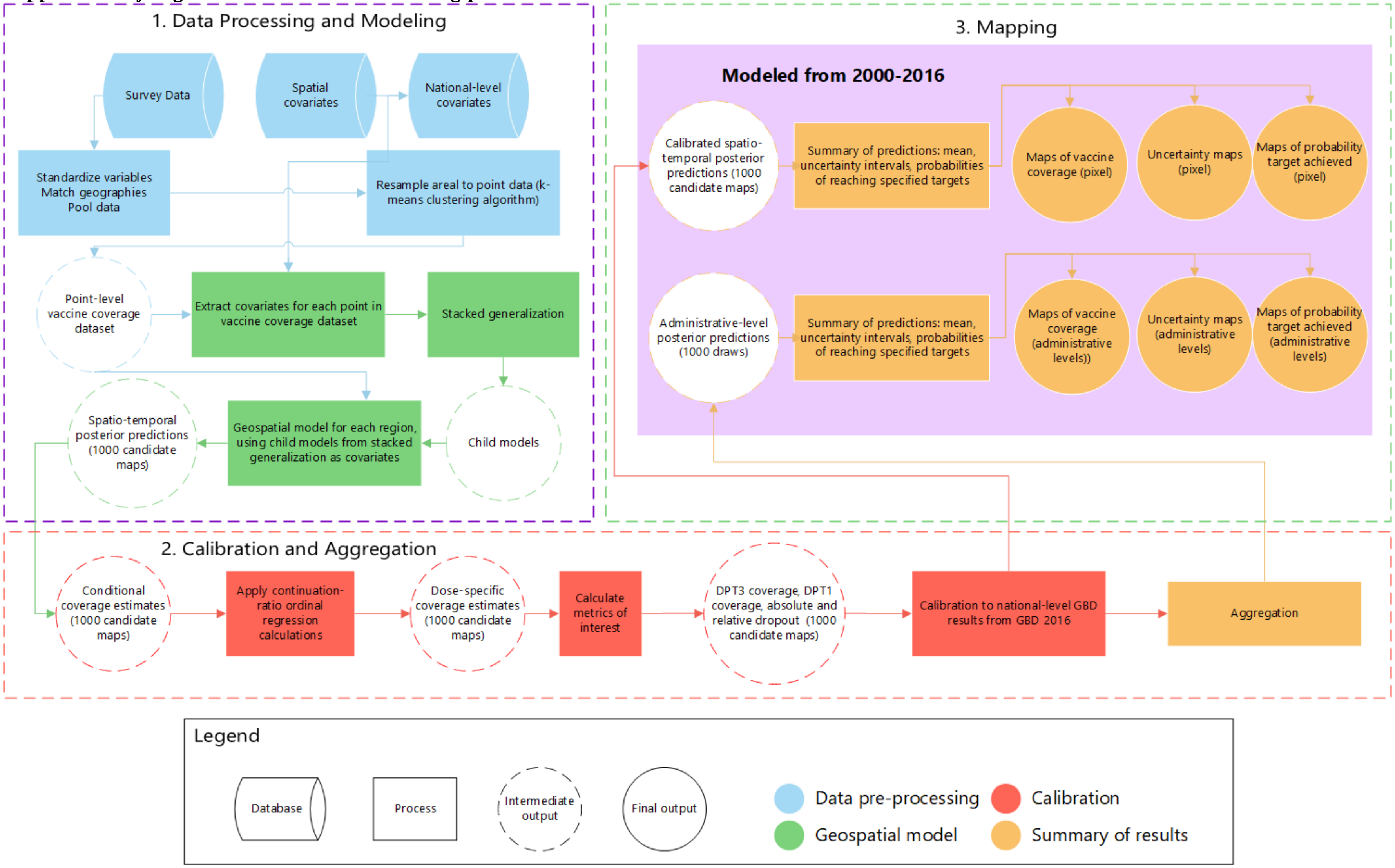

5.2 Regional model geographies

For each modelled vaccine coverage quantity, five regional geostatistical models were fit, then combined to create the final continental model of vaccine coverage. Models were run by region for two primary reasons. First, the continental scope of this analysis requires substantial computational resources, and fitting regional models improves computational feasibility when estimating with 1,000 samples from the posterior and including out-of-sample cross-validation. Second, fitting regional models allows additional subcontinental variation in the relationships between covariates and vaccine coverage and in the hyperparameters that govern the spatiotemporal patterns of vaccine coverage. Countries were assigned to regions within Africa that were chosen to align with the regions used in the Global Burden of Disease study, which group epidemiologically-similar countries into geographically proximate regions. These regions were then modified slightly to ensure geographic contiguity (eFigure 2). For each regional sub-model, data from the modelled region as well as data within a one-degree buffer of the region's boundary were included (in order to minimize regional edge effects).

5.3 Covariate ensemble modelling using stacked generalization

In order to capture potential complex interactions and non-linear relationships between covariates, an ensemble modelling approach (Gaussian Process stacked generalization) was employed, which has been previously shown to improve the predictive accuracy of geostatistical models.[12] This process is shown schematically in Supplementary Figure 6.

For each region and modelled vaccine coverage quantity, three sub-models were fit using the assembled set of spatial covariates as explanatory variables and the vaccine coverage quantity of interest as the outcome: generalized additive models, boosted regression trees, and lasso regression. For the boosted regression tree model, country-level fixed effects were also included in order to allow for differential relationships between covariates and vaccine coverage by country. Note that there are no explicit spatial or temporal effects included in these sub-models, although the underlying covariates are spatially and temporally correlated.

Sample weights were used in the data preparation process as applicable when calculating DPT coverage for areal units with no precise latitude/longitude available. For inclusion in the INLA geospatial model, each latitude/longitude-located cluster was then assigned a weight of 1, and each candidate observation generated by the spatial resampling process for areally-assigned data was assigned a sampling weight from the K-means clustering process as described above in section 3.3.

In order to avoid overfitting, each sub-model was fit using five-fold cross-validation, generating a set of out-of-sample predictions of vaccine coverage for each modelled location and year in the particular region. Each model was also fit with a full set of vaccine coverage outcome data, generating a corresponding set of in-sample predictions. The generated out-of-sample predictions for each of the three sub-models were included as explanatory covariates when fitting the geostatistical model described below, while the in-sample predictions from each of the three sub-models were used when generating predictions from the fitted geostatistical model. To illustrate the results of this process, the predicted in-sample vaccine coverage layers generated from the three sub-models (generalized additive models, boosted regression trees, and lasso regression) for 2016 (used to generate predictions from the geostatistical model) are shown in Supplementary Figure 7.

Supplementary Figure 6: Example of covariate ensemble modelling
Using covariates and country-level fixed effects (in the case of boosted regression trees only) as explanatory variables and vaccine coverage data as outcome data, three separate sub-models (generalized additive models, lasso regression, and boosted regression trees) are fit using cross-validation to generate in- and out-of-sample predictions. The maps to the right of the figure show an example of the out-of-sample results of this process for a single year for

the Democratic Republic of the Congo. These out-of-sample predicted surfaces are then used as explanatory covariates in the full geostatistical model.

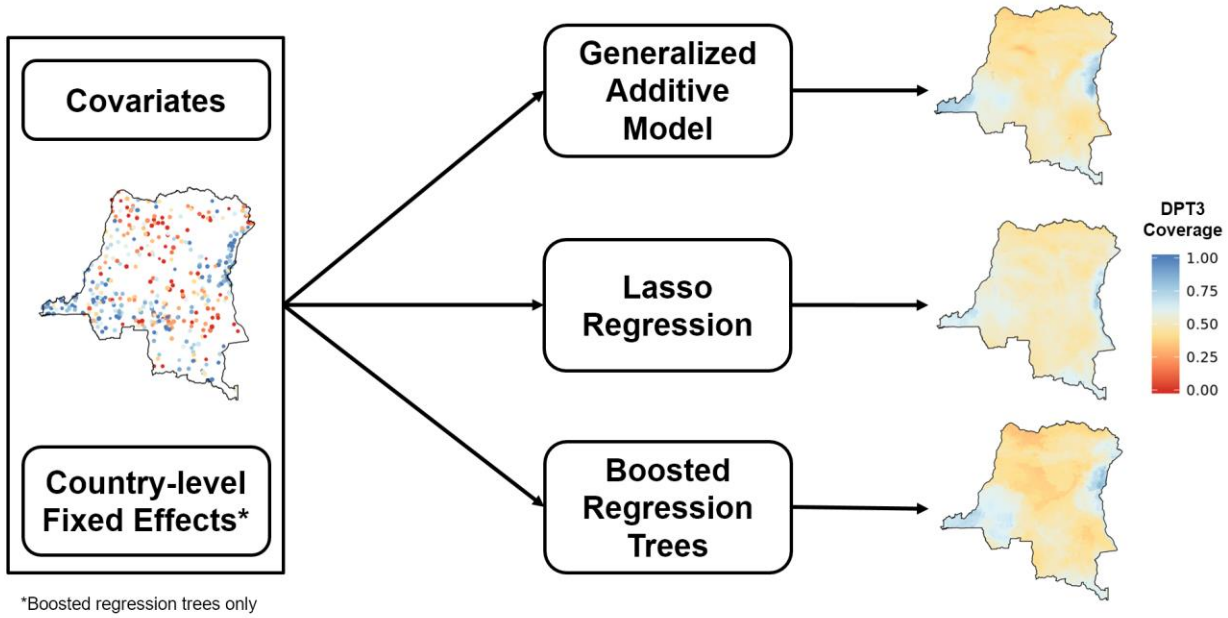

Supplementary Figure 7: Predicted in-sample DPT3 surfaces from the ensemble covariate modelling progress, 2016. Each map represents in-sample predicted DPT3 coverage generated from the three sub-models (GAM, generalized additive models; GBM, gradient boosted models [the particular implementation of boosted regression trees used in this analysis]; and lasso regression) for 2016. These surfaces were as covariates in the generation of predictions from the geostatistical model.

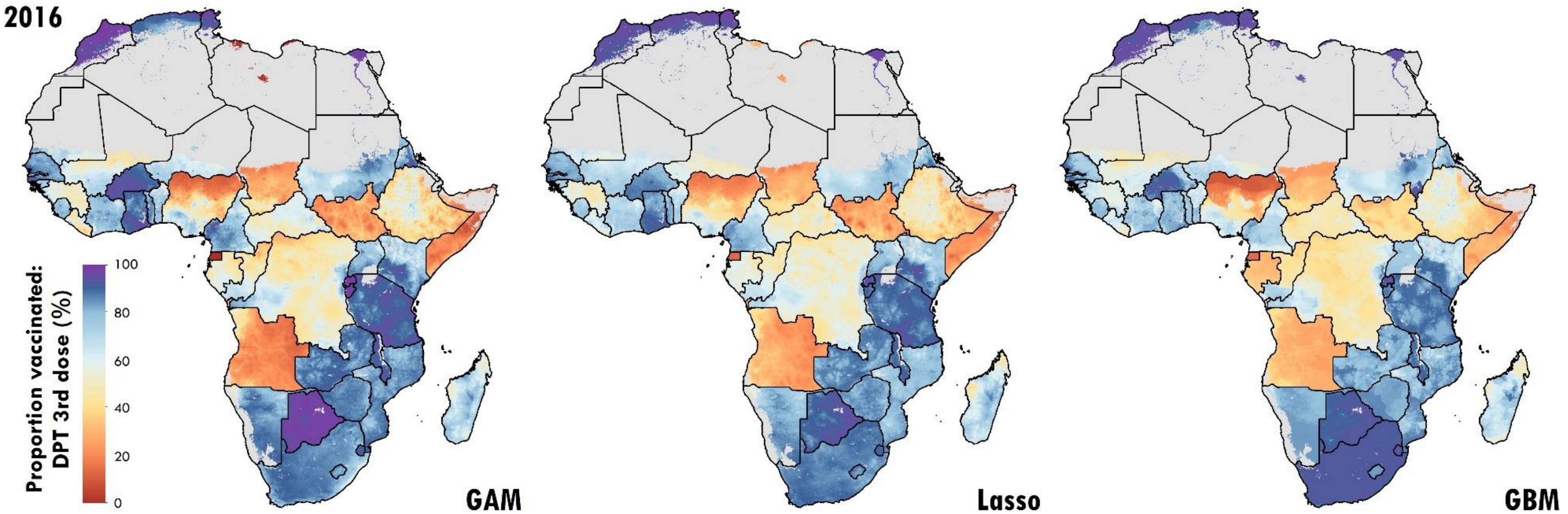

5.3.1 Covariate extrapolation

Both at the level of the covariate ensemble model and in the full geostatistical model, relationships between covariates and vaccine coverage are leveraged in order to generate predictions of vaccine coverage in places and times when no survey observations are available (the full geostatistical model additionally leverages spatiotemporal patterns in coverage). These predictions require the assumption that the relationship between covariates and outcome is similar between places and times where data is available and those where no observations have been recorded.

In addition, for some places and times, estimates may be generated in places where covariate values extend beyond the range of covariate values for which observed data is available. In these cases, the estimates generated represent extrapolations from observed covariate-vaccine coverage relationships in places where data are available. The extent to which these reported estimates represent extrapolations of covariate values (for the representative example of DPT3 coverage, as DPT1 coverage and dropout were estimated using the same set of data) is summarized in Supplementary Figure 8.

Supplementary Figure 8: Covariate extrapolation by model region
Each plot shows the distribution of pixel values across all years for DPT3 coverage estimated by each of the sub-models (Generalized Additive Models [GAM], Gradient Boosted Model [GBM, the specific implementation of boosted regression trees used in this analysis], and lasso regression) from the ensemble modelling approach explained above, which are used as the covariates in INLA. Two distributions are displayed: pixel values corresponding to points in space and time where data observations are observed, and those corresponding to points in space and time where no data was available. Pixels without observed data which fall outside of the distribution of observed data represent points to which covariate extrapolations were made.

A) Western Sub-Saharan Africa

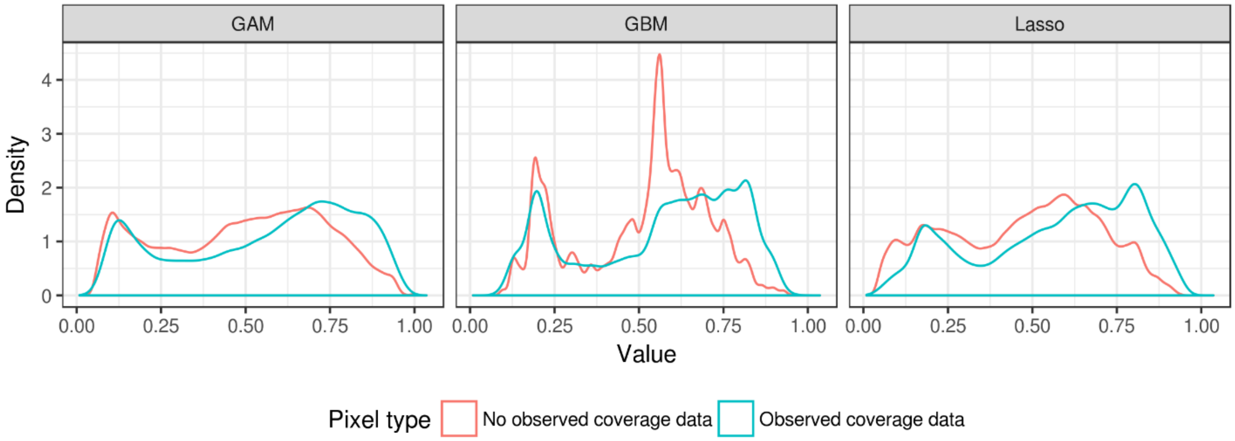

B) Central Sub-Saharan Africa

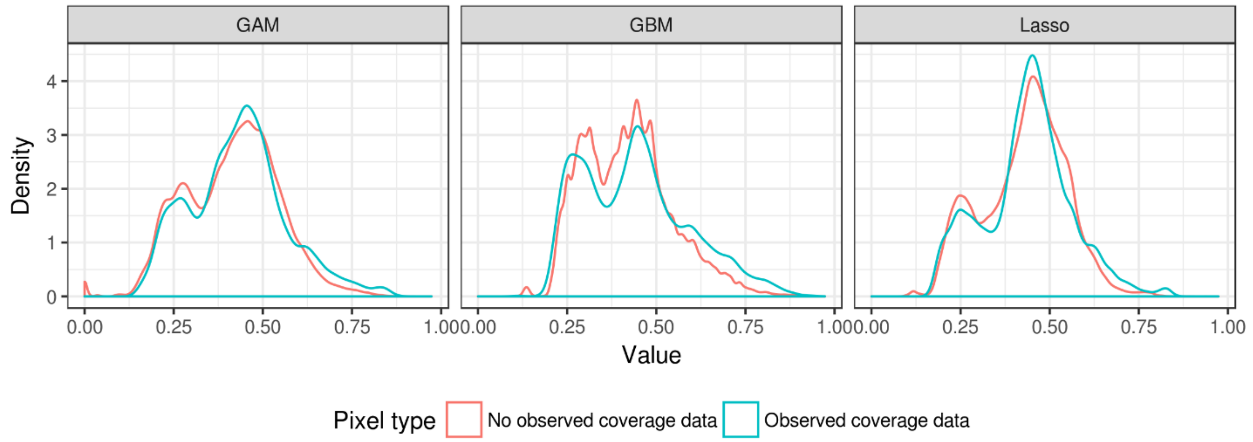

C) Eastern Sub-Saharan Africa

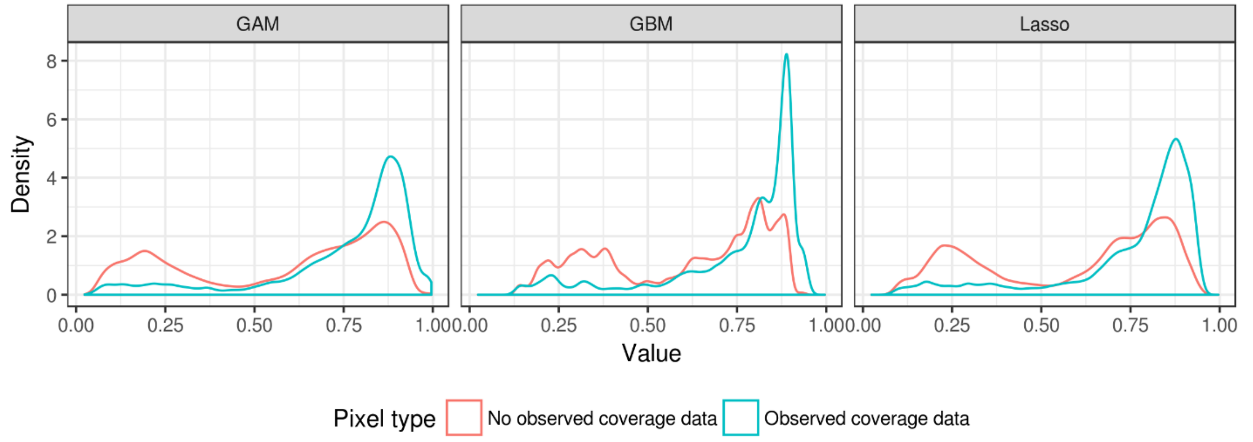

D) Southern Sub-Saharan Africa

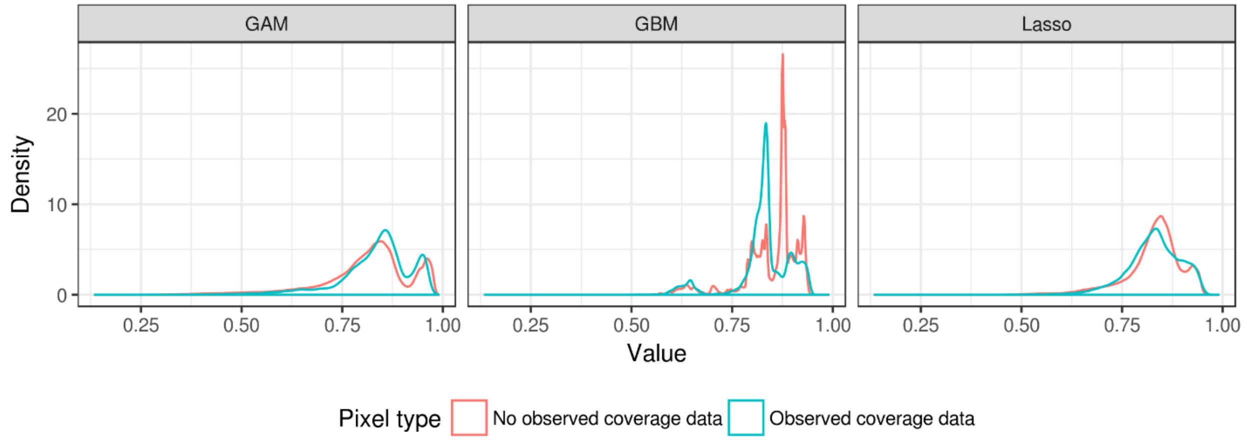

E) North Africa

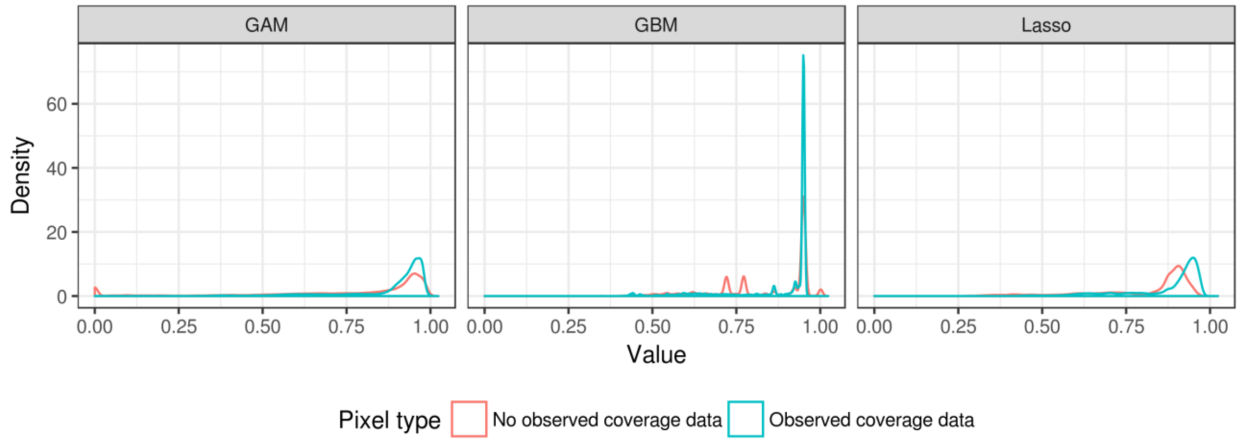

5.3.2 Relative covariate importance

The covariate ensemble modelling process used in this analysis emphasizes improvements in predictive validity, at the cost of inferential analysis. In the final geostatistical model, the predicted DPT surfaces from each of the sub-models (GAM, boosted regression trees, and lasso regression) are used as predictors of coverage, rather than the covariates themselves. This allows for complex and non-linear interactions between covariates.

This complexity, however, makes the analysis of covariate-coverage relationships difficult. In addition, some of the covariates (i.e. maternal education, malaria incidence) are themselves modelled, and the models used to produce these spatial estimates utilize some of the same covariates as are included in the DPT coverage models. Any analysis which attempts to infer which covariates drive the observed coverage patterns in this model, therefore, should be undertaken with caution. Further methods are under development to overcome these limitations, in order to allow simultaneous high predictive ability and inferential analysis.

With those caveats, the relative importance of each covariate within each component of the ensemble modelling process can give some insight into which covariates are most influential in the overall model. Supplementary Figure 9 shows relative covariate importance plots for DPT3 for each modelled region. For each of the three sub-models used in the ensemble modelling process, a relevant measure of covariate importance was calculated. For the GAM model, the negative logs of covariate p-values were used; for boosted regression trees, relative influence; and for the lasso regression, the Agresti method of generating standardized coefficients.[13] These relative importance metrics were then normalized to sum to one within each sub-model.

To summarize the overall relative importance of each covariate in the full DPT3 model, a weighted average of these sub-model-specific relative importance values was calculated for each covariate, using the beta coefficients for each sub-model in the final geostatistical model as weights. In general, antenatal care, fertility, maternal education, lag-distributed income per capita, and mortality rate due to war and terror in the last 10 years tended to have the highest relative importance scores across modelled regions, although the precise patterns varied by region.

Supplementary Figure 9: Relative covariate importance plots.
A separate plot is presented for each modelling region. In each plot, the top three rows each represent a sub-model in the covariate ensemble modelling process, and each column represents a covariate. The colour within a given cell represents the relative importance of the covariate within a given model. The “INLA combined” row at the bottom of each plot displays a weighted average of these sub-model relative importance values for each covariate, using the

beta coefficients from the final fitted geostatistical model as weights. This serves as a proxy for the overall influence of the covariate in the final model

A) CSSA modelling region

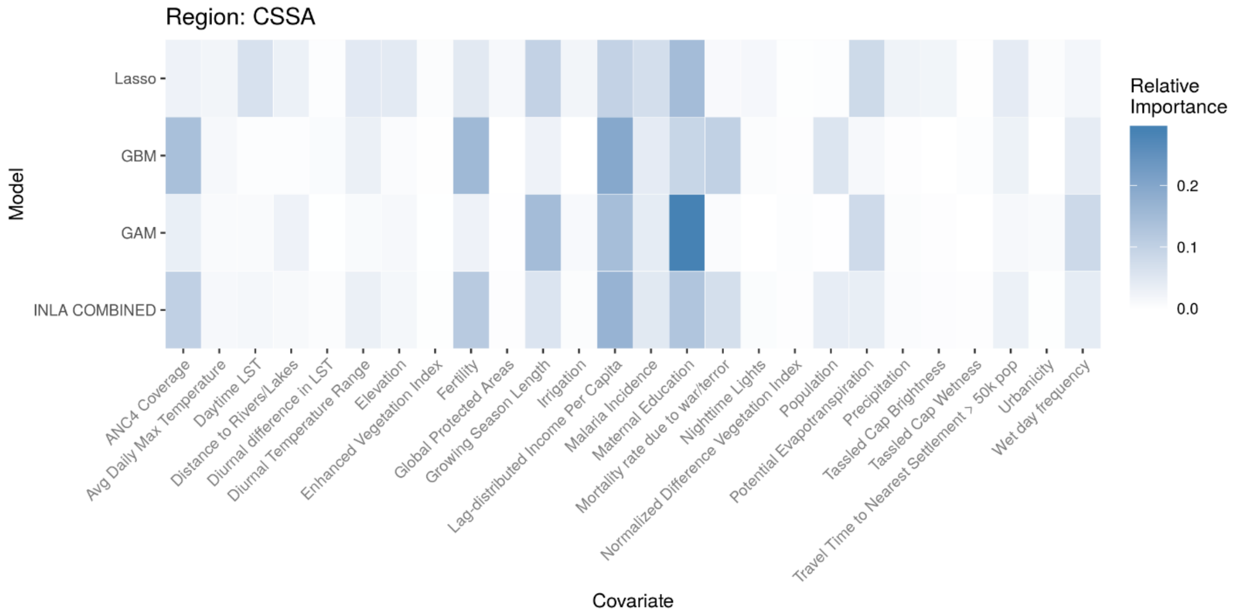

B) ESSA modelling region

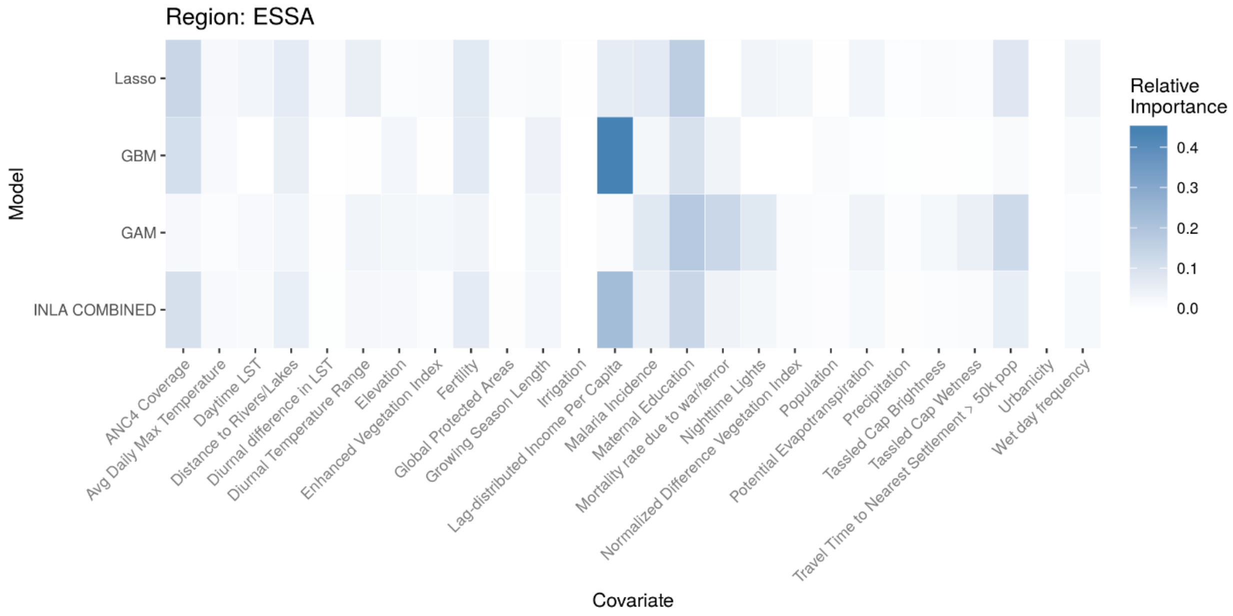

C) NAME modelling region

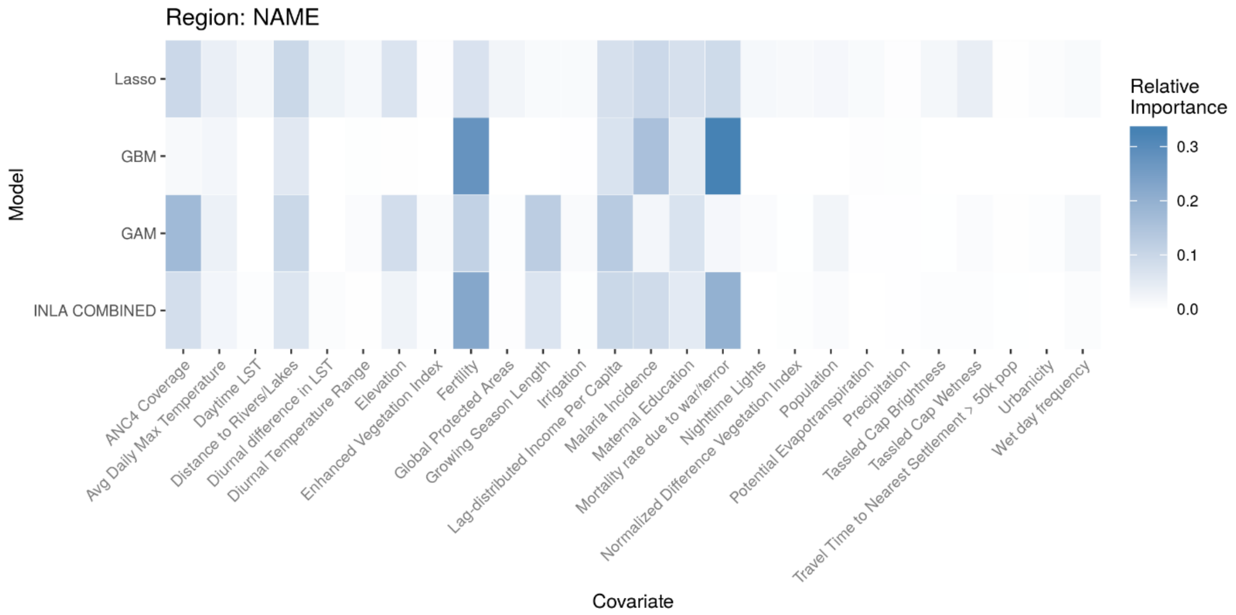

D) SSSA modelling region

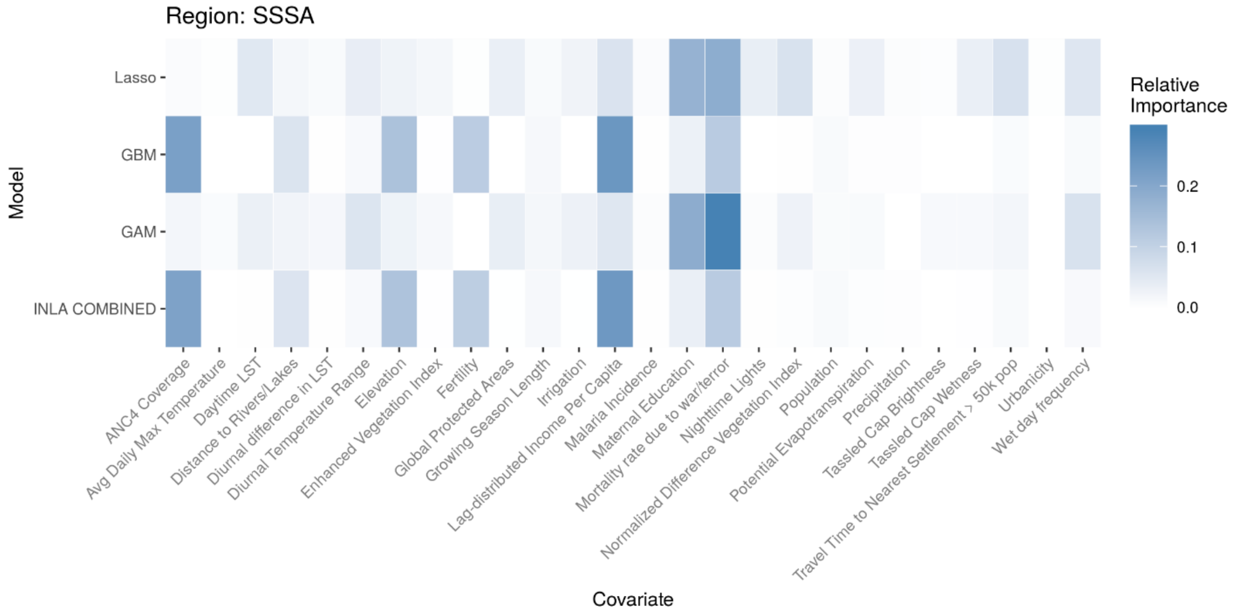

E) WSSA modelling region

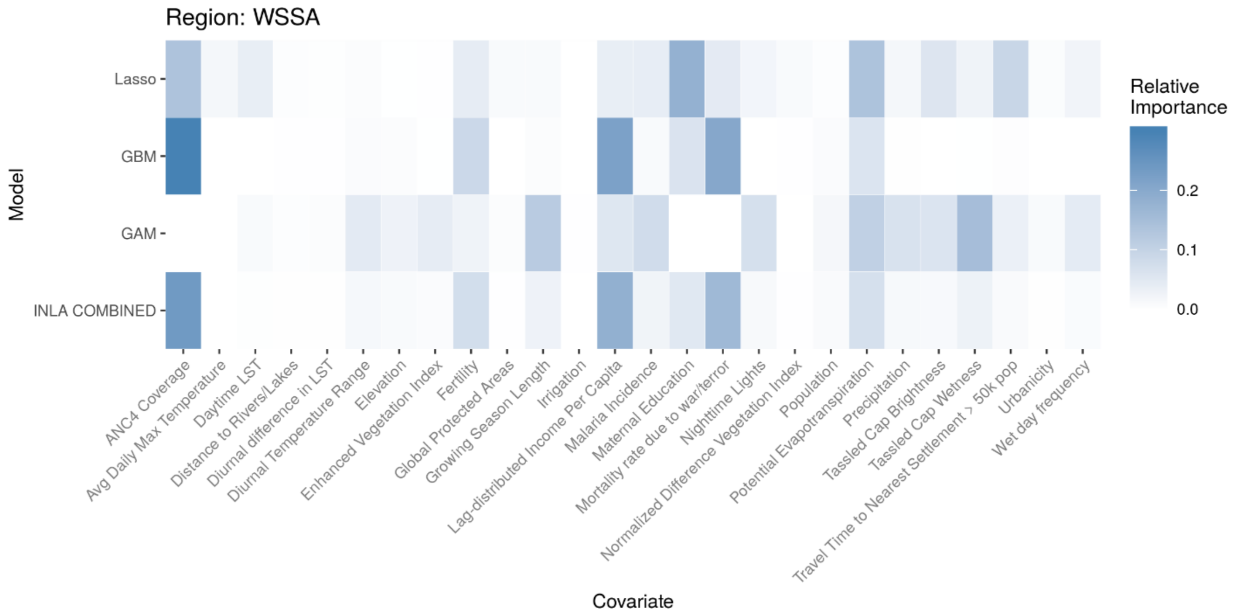

5.4 Geostatistical Model

5.4.1 Model description

Vaccine coverage was modelled as binomial count data with a logit link function using a Bayesian geostatistical modelling framework consisting of a hierarchical spatially- and temporally-explicit generalized linear regression model. For each modelled vaccine coverage quantity, the prevalence of vaccination was fit for each of the five modelling regions within Africa, defining the Bayesian model as follows:

$$C_i | p_i,\ N_i \sim \text{Binomial}(p_i,\ N_i)$$

$$\text{logit}(p_i) = \ \beta_0 + \boldsymbol{X_i \beta} + \epsilon_{GP_i} + \epsilon_{ctry_i} + \epsilon_i$$

$$\sum \boldsymbol{\beta} \ = 1$$

$$\epsilon_i \sim N\left(0, \sigma_{nug}^2\right)$$

$$\epsilon_{ctry} \sim N(0, \sigma_{ctry}^2)$$

$$\epsilon_{GP} | \Sigma_{\text{space}},\ \Sigma_{time} \sim GP(0,\ \Sigma_{\text{space}} \otimes\ \Sigma_{time})$$

$$\Sigma_{\text{space}} = \frac{2^{1-\nu}}{\tau \times \Gamma(\nu)} \times (\kappa \boldsymbol{D})^{\nu} \times \mathrm{K}_{\nu}(\kappa \boldsymbol{D})$$

$$\Sigma_{time\ j,k} = \rho^{|\ t_k - t_j|} \text{ for } time_j, time_k \in \{2000, 2005.33, 2010.66, 2016\}$$

This geostatistical model has been detailed in previous work[5–7]. Briefly, this framework models the number of children in cluster i with sample size N_i who have been vaccinated with a given number of doses of the vaccine of

interest as binomial count data, C_i. The probabilities p_i represent the annual prevalence of vaccination among children aged 12-23 months with the specified number of doses of vaccine at given location in space and time (vaccine coverage). For simplicity, notation has been suppressed, but counts C_i, probabilities p_i, predictions from the three submodels $\boldsymbol{X_i}$, and residual terms ϵ_* are all specific to a particular location in space and time across the modelled region and all years in the modelled time range.

Using a generalized linear model approach, the logit of annual prevalence, p_i, of vaccination was modelled as a linear combination of:

- $\boldsymbol{X_i}$, the logit of the out-of-sample predictions of vaccine coverage obtained from three sub-models (GAM, BRT, and lasso) generated from ensemble covariate modelling as described above in section 5.3 (Covariate ensemble modelling using stacked generalization),
- ϵ_{GP_i}, a correlated spatiotemporal error term,
- ϵ_{ctry_i}, a country-level random effect, and
- ϵ_i, an independent nugget effect.

The coefficients $\boldsymbol{\beta}$ on the sub-models in this generalized linear modelling framework represent their predictive weighting and were constrained to sum to 1. The nugget ϵ_i represents irreducible error for a given observation, which could arise from measurement error or true variation beyond what is captured by the model. The joint error term ϵ_{GP}, then, represents the residual spatial and temporal autocorrelation that remains after accounting for the predictive capacity of the ensemble-modelled covariates, country-specific variation in vaccine coverage, and observation-specific irreducible error.

These spatiotemporal residuals ϵ_{GP} were modelled as a three-dimensional spatiotemporal Gaussian process with a mean of zero and a covariance matrix constructed from the Kroenecker product of spatial and temporal covariance kernels. Temporal covariance Σ_{time} was modelled using an autoregressive order 1 (AR1) function taken over three equal-length periods spanning 2000-2016. Spatial covariance Σ_{space} was modelled using a stationary and isotropic Matérn function[13], which is a function of $\mathbf{D}$, a distance matrix in space-time. The model was fit using integrated nested Laplace approximations in R-INLA[14,15] with the stochastic partial differential equations (SPDE)..

5.4.2 Priors

Priors used for all geostatistical models were defined as follows:

- $\beta_0 \sim \text{N}(\mu = 0, \sigma^2 = 3^2)$,
- $\boldsymbol{\beta} \sim^{iid} \text{N}(\mu = 0, \sigma^2 = 3^2)$,
- $\log\left(\frac{1+\rho}{1-\rho}\right) \sim \text{N}(\mu = 0, \sigma^2 = 1/0.15)$,
- $\log\left(\frac{1}{\sigma^2_{nug}}\right) \sim \text{loggamma}(\alpha = 1, \gamma = 2)$,
- $\log\left(\frac{1}{\sigma^2_{ctry}}\right) \sim \text{loggamma}(\alpha = 1, \gamma = 2)$,
- $\theta_1 = \log(\tau) \sim N(\mu_{\theta_1}, \sigma^2_{\theta_1})$
- $\theta_2 = \log(\kappa) \sim N(\mu_2, \sigma^2_{\theta_2})$.

R-INLA automatically determines uncorrelated multivariate normal priors based on the finite elements mesh for the log-transformed hyperparameters κ and τ for the Matérn function used to model spatial covariance. The mean and variance for these hyperpriors are given in Supplementary Table 5.

Supplementary Table 5: Spatial hyperparameter priors selected by R-INLA, by region

Region	μ_{θ_1}	$\sigma^2_{\theta_1}$	μ_{θ_2}	$\sigma^2_{\theta_2}$
**Central sub-Saharan Africa**	-0.231	10	-1.035	10
**Eastern sub-Saharan Africa**	0.104	10	-1.370	10
**Northern Africa**	0.220	10	-1.486	10
**Southern sub-Saharan Africa**	-0.174	10	-1.092	10
**Western sub-Saharan Africa**	0.182	10	-1.448	10

5.4.3 Spatial mesh construction

In this modelling framework, the stochastic partial differentiation equation (SPDE) approximation to the Gaussian process residuals for fitting of the spatiotemporal correlated error term in the generalized linear modelling framework requires the construction of a finite elements mesh for each modelled region.[17] Finite elements meshes were constructed using edge-smoothed polygon boundaries for each modelled region with the inner mesh triangle maximum edge length set to 0.2 degrees and the buffer triangle maximum edge length set to be 5.0 degrees. Supplementary Figure 10 provides an example of a finite element mesh for a modelling region.

Supplementary Figure 10: Example finite elements mesh.
This figure shows the finite elements mesh used to fit the spatiotemporal correlated error term for Western sub-Saharan Africa. The larger triangles show the buffer region surrounding the modelling region (maximum triangle edge length of 5.0 degrees, while the finer inner mesh overlays the modelling region (maximum triangle edge length of 0.2 degrees). Country boundaries are shown in red, and the simplified polygon used to define the modelling region boundary is shown in blue.

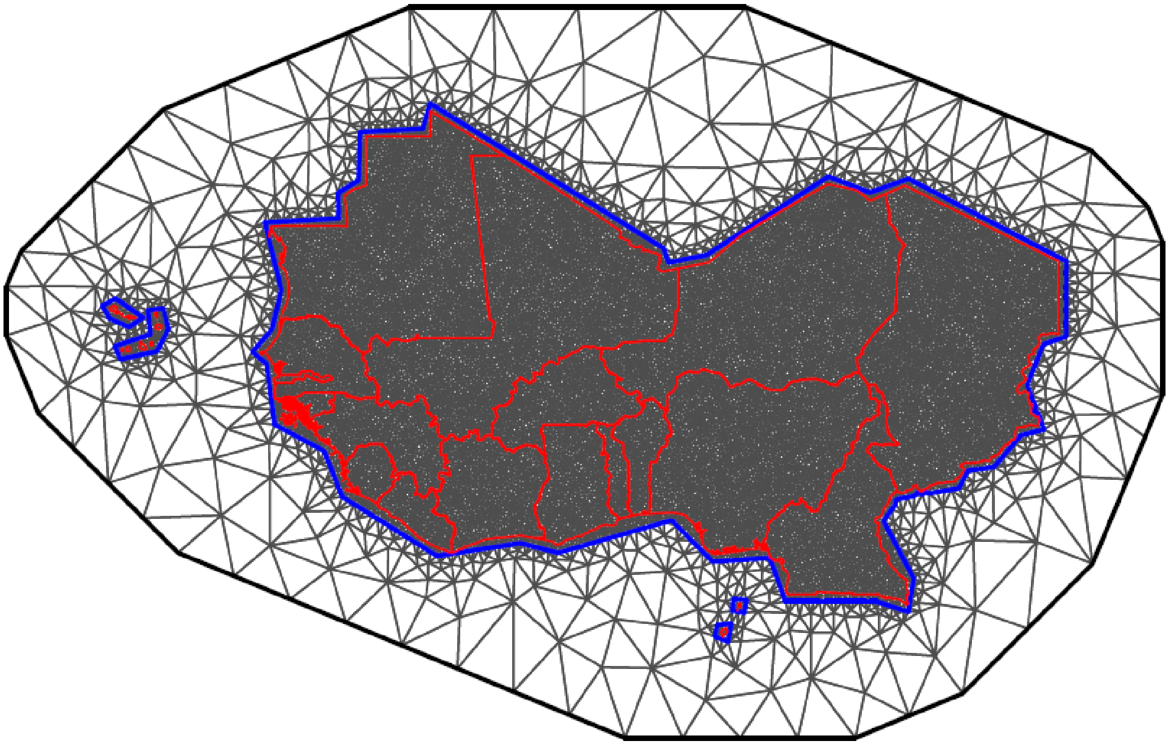

5.4.4 Model fitting and estimate generation

All models were fit in R-INLA using previously-published methods used for the estimation of under-5 mortality[7], education[6], and child growth failure[5]. All cluster-level observations for which a precise latitude and longitude could

be obtained were assigned a weight of 1. For candidate observations generated using spatial resampling (as described in section 3.3, Geographic positioning of clusters and spatial integration of areal data), weights from the K-means clustering process were used during the INLA model fitting process. This process ensures that areal observations and precisely-located observations contribute equally to the log-likelihood within the model.

Estimates of the fitted modelling parameters for each vaccine coverage geostatistical model, by region, can be found in Supplementary Table 6, Supplementary Table 7, and Supplementary Table 8. These parameters include fixed effects beta coefficients representing the relative contribution of each sub-model from the covariate ensemble modelling process as well as estimates of the fitted spatiotemporal field hyperparameters and the precision (inverse variance) for the nugget effect. For each hyperparameter, the median and associated 95% uncertainty intervals are provided. The spatial hyperparameters τ and κ have been transformed into nominal variance and range for ease of interpretation. Nominal variance represents the variance at a single point (calculated as $nominal\ variance\ = 4\pi\kappa^2\tau^2$). Nominal range approximates the distance that can be travelled from a point before spatial correlation decays by 90% (calculated as $range = \sqrt{8}/\kappa$)[18].

5.4.5 Conditional ordinal regression modelling

DPT coverage in a given location can be conceptualized as a distribution of coverage – for each location, there are some children who have received 0 doses, 1 dose, 2 dose, or 3+ doses, and the relative distribution of children who fall into each category varies from location to location. In order to model the full distribution of possible vaccine coverage states, and thereby ensure that estimates of DPT1 coverage, DPT3 coverage, and dropout are internally consistent for every location and time in our models, a conditional ordinal regression framework[19] was used. This method estimates the probabilities of vaccination with 0, 1, 2, or 3+ doses of DPT for each modelled 5x5 km location in each year, by estimating coverage with 3+ doses (DPT3 coverage) and two conditional coverage quantities, as detailed below. Separate geospatial models (including both the covariate ensemble modelling process and the fitting of the hierarchical Bayesian geostatistical model) as described above were fit for DPT3 coverage and two additional conditional vaccine coverage metrics (where *d* is the number of doses received for each child):

A) DPT3 coverage: $p(d \geq 3)$

B) 2-dose conditional coverage: $p(d = 2 \mid d \leq 2)$

C) 1-dose conditional coverage: $p(d = 1 \mid d \leq 1)$

The one- and two-dose conditional coverage geostatistical models therefore estimate conditional probability of having received exactly *d* doses given the receipt of *d* or fewer doses. Conditional coverage for *d* doses was calculated using a subset of the source data used to model DTP3 coverage, but limiting to clusters containing only individuals who had received *d* or fewer doses.

For each of these three geospatial models, identical covariates, priors, and model configuration parameters were used to generate estimates of vaccine coverage by region as detailed above. From the posterior-estimated distributions of all modelled parameters, 1,000 draws (samples) were obtained, where each draw represents a potential set of vaccine coverage values for each 5x5 km location for each year in the modelled time range.

From these three quantities (DPT3 coverage and 2- and 1-dose conditional coverage), and using the relationship that $p(d = 0) + p(d = 1) + p(d = 2) + p(d \geq 3) = 1$, coverage with any number of doses can be calculated arithmetically. The outputs of these three related models were therefore used to arithmetically calculate separate geospatial estimates of the probability of receipt of 0, 1, 2, and ≥ 3 doses of DPT vaccine for each of the 1,000 draws.

Supplementary Figure 11 visualizes these probabilities for a single 5x5 km area and year using a ternary plot. The overall result of this process is to generate 1,000 possible *distributions* of coverage for each 5x5 km area and year in the modelled region.

From these dose-specific coverage estimates, then, the vaccine coverage metrics of interest were then calculated at the draw level: DPT1 coverage, or the probability of receipt of ≥ 1 dose of DPT vaccine; DPT3 coverage, or the probability of receipt of ≥ 3 doses of DPT vaccine, and relative and absolute DPT1-3 dropout.

This continuation ratio ordinal regression modelling framework ensures internal consistency of all estimates at the draw level – that is, for each draw and space-time location, the probabilities of receipt of 0, 1, 2, or ≥ 3 doses of DPT vaccine all sum to one. Other ordinal regression approaches were considered, but the continuation ratio model was chosen as it directly estimates DPT3 coverage, which is the primary metric of interest in this study due to its longstanding role as a key metric of vaccine coverage used to guide policy decisions. Compared to other options for ordinal regression, such as a proportional odds model, a continuation ratio model allows for flexibility in the relationships between covariates and the odds of vaccination with 0, 1, 2, or ≥ 3 doses of DPT. As Supplemental Tables 6-8 and Supplementary Figure 16 demonstrate, the covariate-coverage relationships vary between the geospatial models, further supporting the choice of the continuation ratio method.

The resultant distributions of the metrics of interest (DPT1, DPT3, and dropout) were summarized as means and 95% uncertainty intervals. As the range of mean vaccine coverage varies broadly across Africa, comparisons of uncertainty are challenging. The Coffey-Feingold-Bromberg metric, which provides a normalized measure of uncertainty for a set of proportions[20] was therefore used to assess draw-level uncertainty (Supplementary Figures 12-15).

Supplementary Figure 11: Ternary plot of estimates of the distribution of vaccination at a single 5x5 km pixel, by draw

Left: a guide to interpretation of the ternary plot. For the purposes of the ternary plot, incomplete vaccination (having received 1 or 2 doses) of vaccine are grouped together. Each triangle edge functions as an axis representing the proportion of children in the particular year and 5x5 km location who have received 0 doses (upper-left axis), 1 or 2 doses (upper-right axis), or 3 doses (bottom axis). The location of the point in ternary space can be interpreted by drawing lines to the appropriate axes following the triangular grid – in the example to the left, 20% of children received 0 doses of vaccine, 70% received 1 or 2 doses, and 10% received 3 (or more) doses. Right: a sample ternary plot from a randomly sampled pixel. Each point represents a single draw; the concentric lines in blue represent 50%, 90%, and 95% uncertainty intervals (from inner to outer).

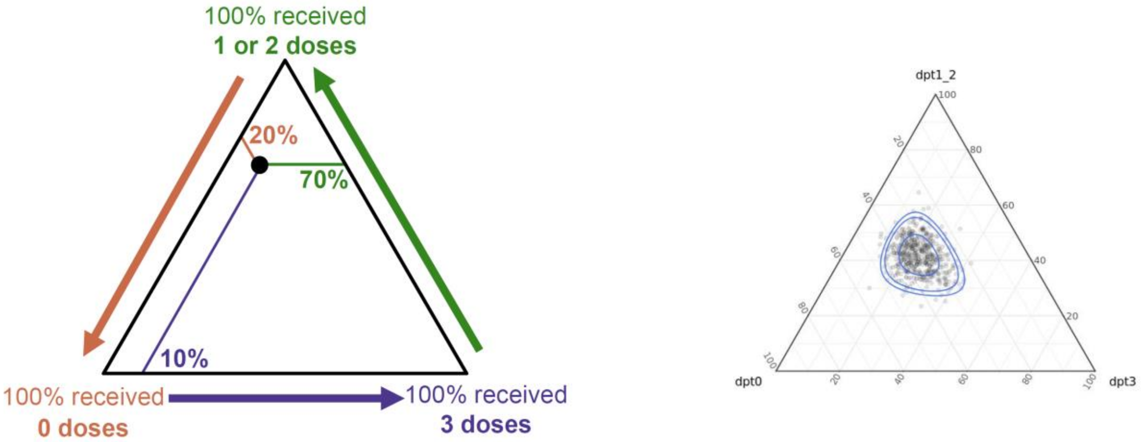

Supplementary Figure 12: **Model uncertainty for DPT3 coverage, 2000 – 2016**. Model uncertainty for DPT3 coverage at the 5x5 km level in 2000 (a), 2010 (b), and 2016 (c) and at the second administrative level for 2000 (d), 2010 (e), and 2016 (f). Model uncertainty is displayed using the Coffey-Feingold-Bromberg metric (CFB), a measure of uncertainty that is comparable regardless of mean coverage and scales from 0% (no uncertainty) to 100% (highest possible uncertainty for a given mean). Results are masked in grey where total population density was less than 10 individuals per 1 km pixel in 2015 per WorldPop[1] estimates, or where land cover was classified as "barren or sparsely vegetated" based on MODIS[2] satellite data in 2013.[1] estimates, or where land cover was classified as "barren or sparsely vegetated" based on MODIS satellite data in 2013.

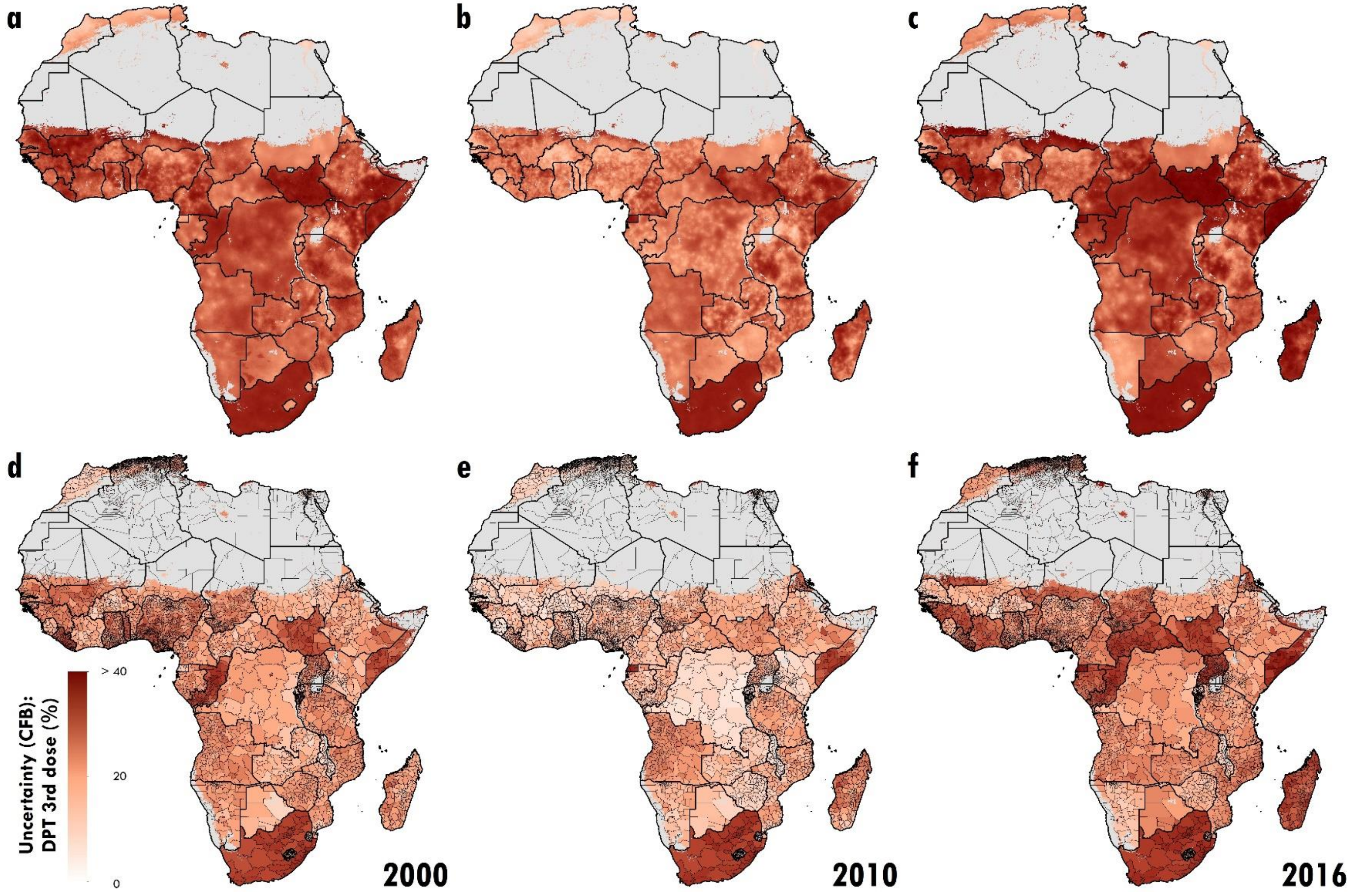

Supplementary Figure 13: Model uncertainty for DPT1 coverage, 2000-2016

Model uncertainty for DPT1 coverage at the 5x5 km level in 2000 (a), 2010 (b), and 2016 (c) and at the second administrative level for 2000 (d), 2010 (e), and 2016 (f). Model uncertainty is displayed using the Coffey-Feingold-Bromberg metric (CFB), a measure of uncertainty that is comparable regardless of mean coverage and scales from 0% (no uncertainty) to 100% (highest possible uncertainty for a given mean). Results are masked in grey where total population density was less than 10 individuals per 1 km pixel in 2015 per WorldPop[1] estimates, or where land cover was classified as "barren or sparsely vegetated" based on MODIS[2] satellite data in 2013.[1] estimates, or where land cover was classified as "barren or sparsely vegetated" based on MODIS satellite data in 2013.

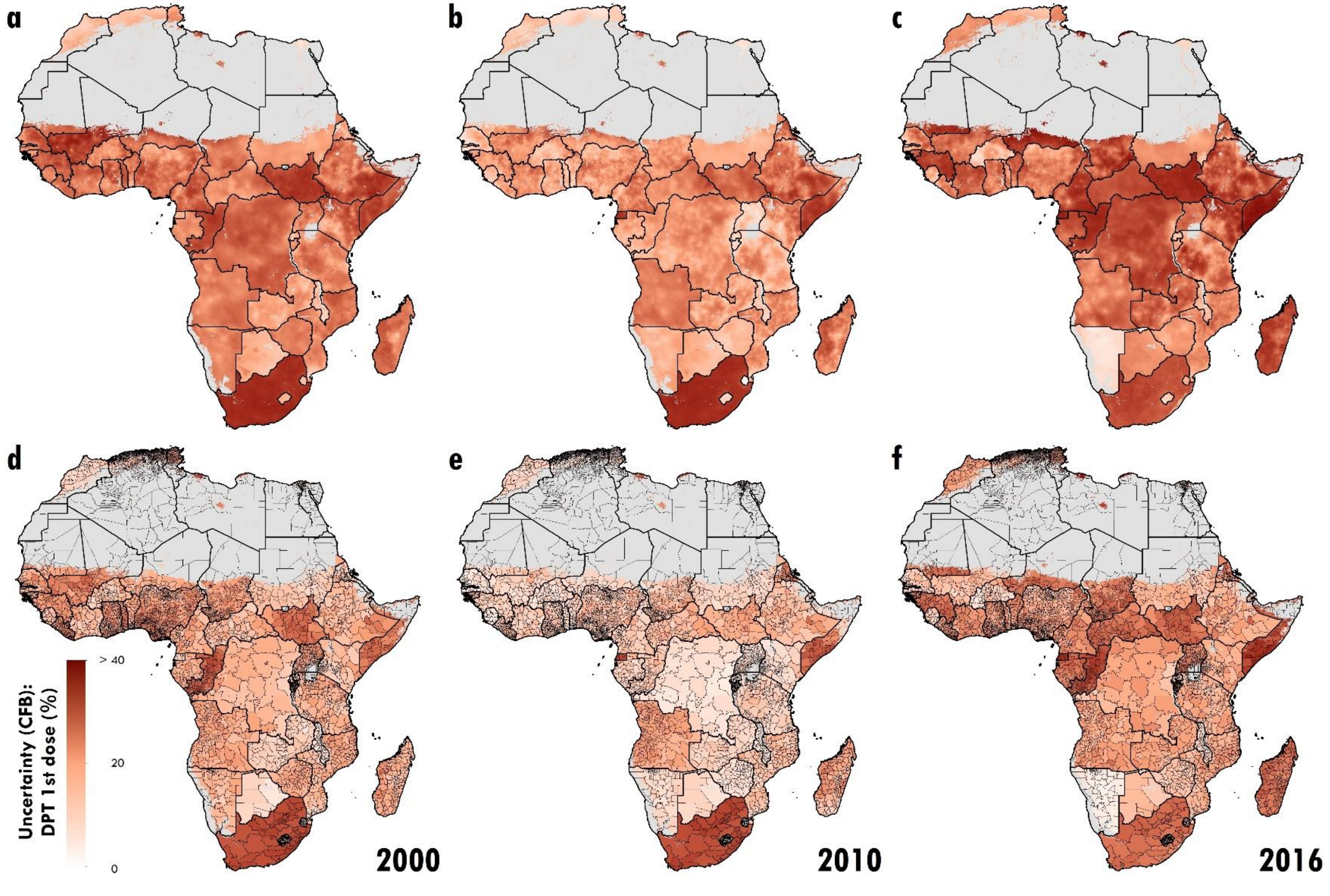

Supplementary Figure 14: Model uncertainty for DPT1-3 absolute dropout, 2000-2016

Model uncertainty for DPT1-3 absolute dropout at the 5x5 km level in 2000 (a), 2010 (b), and 2016 (c) and at the second administrative level for 2000 (d), 2010 (e), and 2016 (f). Model uncertainty is displayed using the Coffey-Feingold-Bromberg metric (CFB), a measure of uncertainty that is comparable regardless of mean coverage and scales from 0% (no uncertainty) to 100% (highest possible uncertainty for a given mean). Results are masked in grey where total population density was less than 10 individuals per 1 km pixel in 2015 per WorldPop[1] estimates, or where land cover was classified as “barren or sparsely vegetated” based on MODIS[2] satellite data in 2013.[1] estimates, or where land cover was classified as “barren or sparsely vegetated” based on MODIS satellite data in 2013.

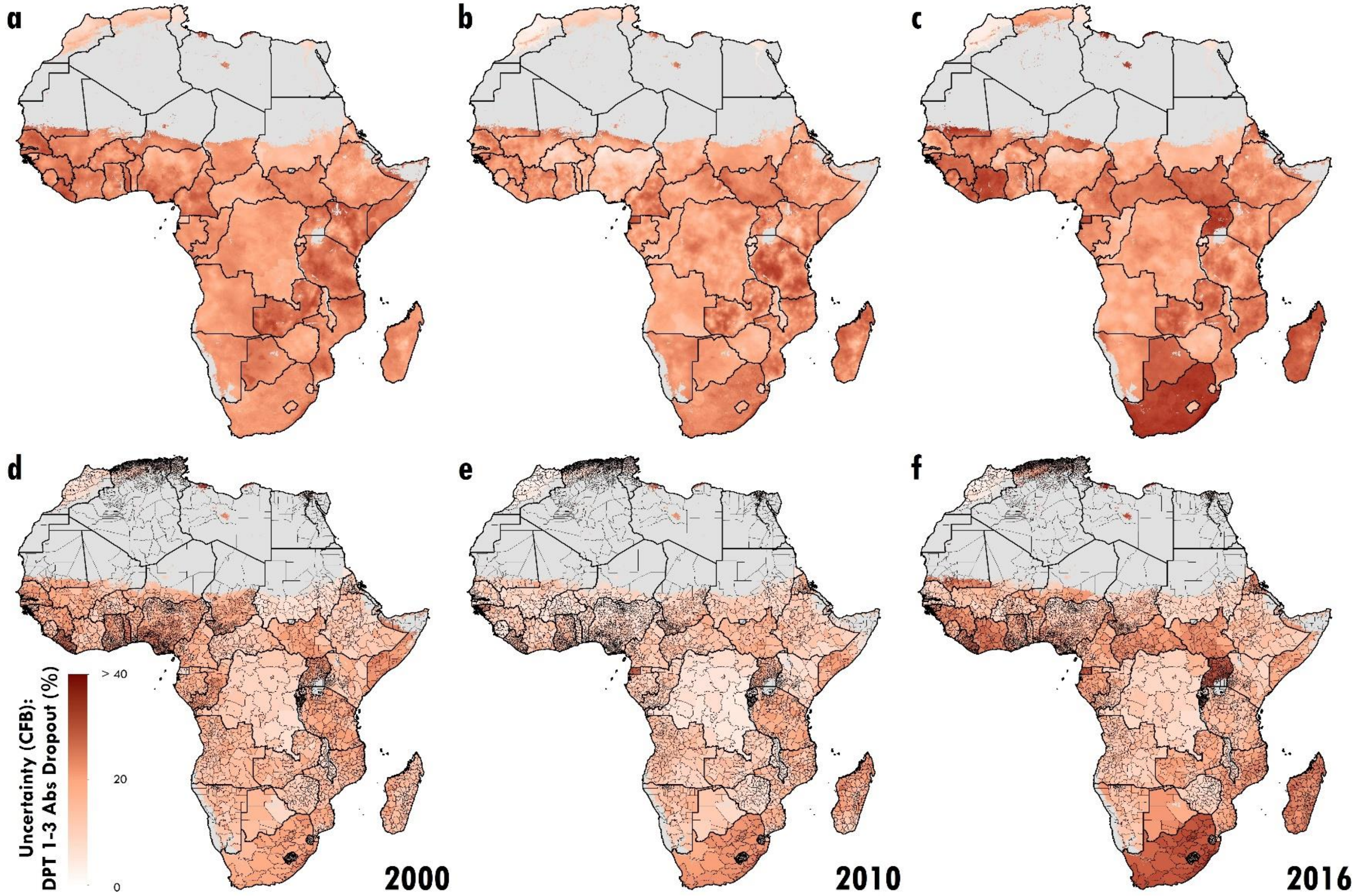

Supplementary Figure 15: Comparison of uncertainty and coverage, 2016.
Uncertainty for DPT3 coverage (a), DPT1 coverage (b), DPT1-DPT3 relative dropout (c), and DPT1-DPT3 absolute dropout (d) among 12-23 month old children at the 5x5 kilometre resolution in 2016. Each map includes a bivariate colour scale with the relative magnitude of the relevant indicator on the vertical axis (grey to red) and the relative magnitude of uncertainty as measured by the Coffey-Feingold-Bromberg metric. Results are masked in grey where total population density was less than 10 individuals per 1 km pixel in 2015 per WorldPop[1] estimates, or where land cover was classified as “barren or sparsely vegetated” based on MODIS[2] satellite data in 2013.

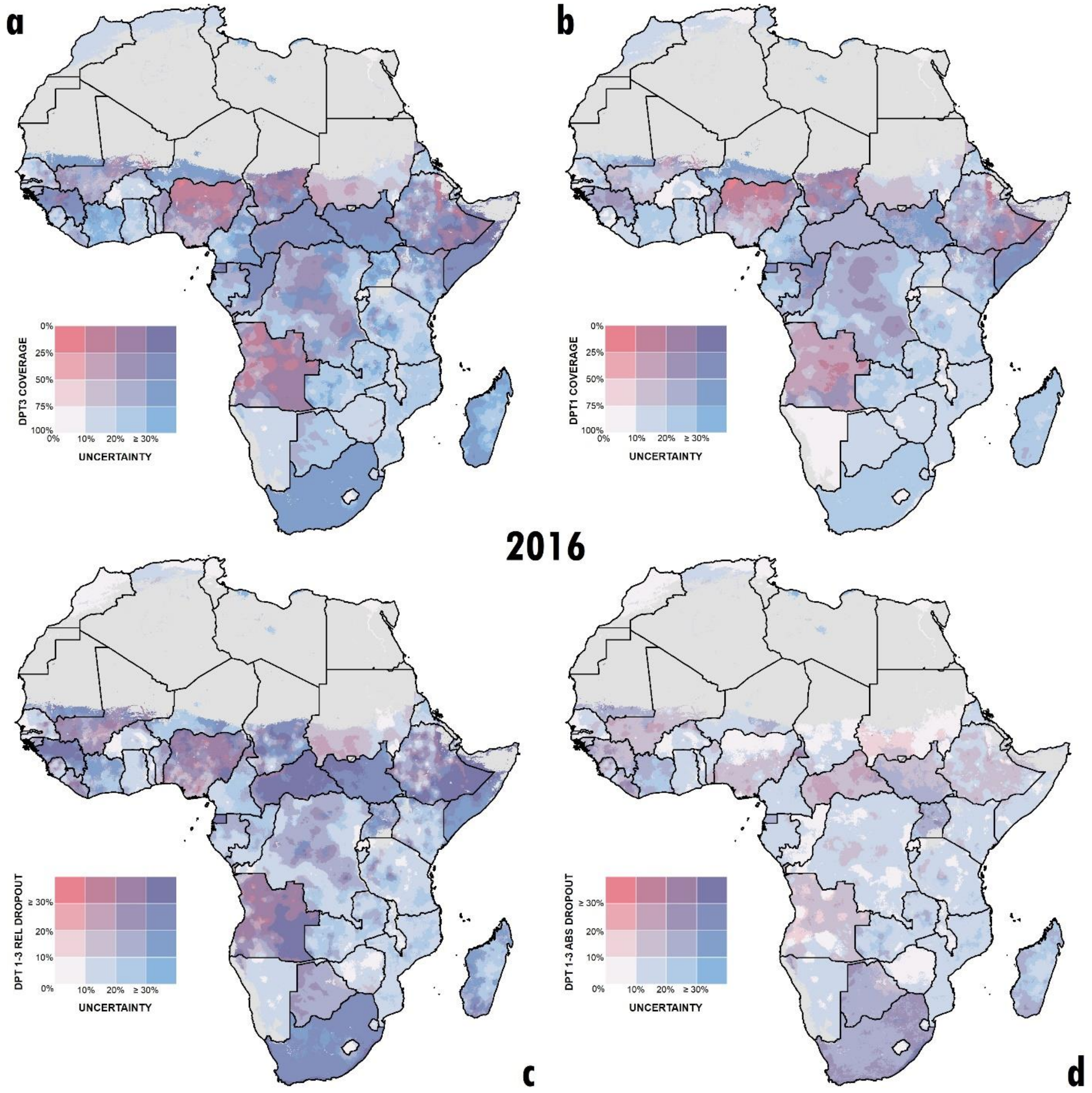

Supplementary Table 6: **Fitted geostatistical model parameters for DTP3 coverage**

GAM: Generalized additive model; GBM: gradient boosted model (the specific implementation of boosted regression trees used here); AR1: autoregressive order 1 function.

	Central sub-Saharan Africa quantiles			Eastern sub-Saharan Africa quantiles			Northern Africa quantiles			Southern sub-Saharan Africa quantiles			Western sub-Saharan Africa quantiles		
	**0.025**	**0.500**	**0.975**	**0.025**	**0.500**	**0.975**	**0.025**	**0.500**	**0.975**	**0.025**	**0.500**	**0.975**	**0.025**	**0.500**	**0.975**
**GAM β**	0.00	0.12	0.25	0.05	0.15	0.25	0.42	0.56	0.71	-0.20	-0.01	0.17	0.10	0.17	0.23
**GBM β**	0.57	0.64	0.72	0.41	0.49	0.56	0.63	0.75	0.88	0.84	0.96	1.08	0.76	0.82	0.87
**Lasso β**	0.10	0.23	0.37	0.24	0.36	0.48	-0.48	-0.32	-0.15	-0.16	0.05	0.26	-0.06	0.02	0.09
**Nominal Range**	1.87	2.19	2.50	1.20	1.30	1.41	2.71	3.75	5.04	1.44	1.89	2.49	1.08	1.17	1.28
**Nominal Variance**	0.52	0.62	0.73	0.84	0.91	0.97	0.25	0.37	0.52	0.26	0.34	0.46	0.80	0.86	0.92
**AR1 *ρ***	0.16	0.36	0.47	0.47	0.57	0.73	0.10	0.34	0.56	0.13	0.34	0.53	0.18	0.23	0.28
**Precision for nugget effect**	34.40	42.00	52.02	26.45	32.63	43.84	25.56	32.76	41.79	15.32	20.95	28.22	24.41	30.72	40.29

Supplementary Table 7: **Fitted geostatistical model parameters for the 2-dose conditional coverage model**
The modelled quantity represents the probability of having received exactly 2 doses among those who have received 2 or fewer doses. GAM: Generalized additive model; GBM: gradient boosted model (the specific implementation of boosted regression trees used here); AR1: autoregressive order 1 function.

	Central sub-Saharan Africa quantiles			Eastern sub-Saharan Africa quantiles			Northern Africa quantiles			Southern sub-Saharan Africa quantiles			Western sub-Saharan Africa quantiles		
	**0.025**	**0.500**	**0.975**	**0.025**	**0.500**	**0.975**	**0.025**	**0.500**	**0.975**	**0.025**	**0.500**	**0.975**	**0.025**	**0.500**	**0.975**
**GAM β**	-0.22	-0.09	0.03	-0.36	-0.20	-0.04	-0.48	-0.19	0.09	-0.23	0.05	0.32	-0.29	-0.16	-0.04
**GBM β**	1.16	1.23	1.31	1.13	1.23	1.33	1.05	1.25	1.46	0.75	0.95	1.14	1.14	1.21	1.29
**Lasso β**	-0.26	-0.14	-0.01	-0.19	-0.03	0.13	-0.32	-0.06	0.19	-0.25	0.01	0.26	-0.18	-0.05	0.08
**Nominal Range**	1.55	1.99	2.59	1.36	1.66	1.99	1.39	4.85	24.16	1.63	3.85	9.53	1.27	1.46	1.65
**Nominal Variance**	0.10	0.14	0.18	0.22	0.26	0.31	0.00	0.01	0.06	0.02	0.07	0.19	0.31	0.35	0.40
**AR1 ρ**	0.12	0.40	0.64	0.03	0.21	0.35	-0.84	-0.18	0.64	-0.76	-0.23	0.53	-0.06	0.05	0.15
**Precision for nugget effect**	28.52	35.76	45.60	23.37	31.04	39.54	16.23	21.92	29.68	10.96	15.72	22.32	42.68	51.41	63.17

Supplementary Table 8: **Fitted geostatistical model parameters for the 1-dose conditional coverage model**

The modelled quantity represents the probability of having received exactly 1 dose among those who have received 1 or 0 doses. GAM: Generalized additive model; GBM: gradient boosted model (the specific implementation of boosted regression trees used here); AR1: autoregressive order 1 function.

	Central sub-Saharan Africa quantiles			Eastern sub-Saharan Africa quantiles			Northern Africa quantiles			Southern sub-Saharan Africa quantiles			Western sub-Saharan Africa quantiles		
	**0.025**	**0.500**	**0.975**	**0.025**	**0.500**	**0.975**	**0.025**	**0.500**	**0.975**	**0.025**	**0.500**	**0.975**	**0.025**	**0.500**	**0.975**
**GAM β**	-0.10	0.00	0.11	-0.21	-0.05	0.07	-0.19	-0.01	0.18	-0.57	-0.27	0.03	-0.50	-0.36	-0.22
**GBM β**	1.07	1.14	1.22	1.10	1.19	1.28	1.12	1.27	1.42	1.00	1.18	1.35	1.46	1.54	1.61
**Lasso β**	-0.25	-0.15	-0.04	-0.27	-0.13	0.03	-0.41	-0.26	-0.12	-0.18	0.09	0.36	-0.32	-0.18	-0.04
**Nominal Range**	1.80	2.27	2.94	1.33	1.63	1.99	-1.39	0.38	46.51	0.46	2.94	23.97	1.24	1.48	1.68
**Nominal Variance**	0.14	0.18	0.25	0.27	0.33	0.40	0.00	0.00	0.00	0.00	0.01	0.09	0.34	0.40	0.46
**AR1 *ρ***	-0.23	-0.01	0.22	-0.15	0.01	0.16	-0.99	-0.06	0.99	-0.90	-0.26	0.76	-0.20	-0.09	0.02
**Precision for nugget effect**	25.51	32.44	41.78	18.30	23.86	33.18	12.68	17.68	24.92	8.04	11.89	18.22	32.62	40.30	50.59

Supplementary Figure 16: Beta coefficients for ensemble sub-models.
Estimates of the beta coefficients (median and 95% uncertainty interval) from the fitted geostatistical models (1-dose conditional DPT coverage, 2-dose conditional DPT coverage, and DPT3 coverage) for each ensemble sub-models: GAM (generalized additive model), GBM (gradient boosted model, the specific implementation of boosted regression trees used here), and lasso regression. Coefficients are constrained to sum to one. In general, GBM ensemble sub-models were the most influential across all modelled quantities and regions.

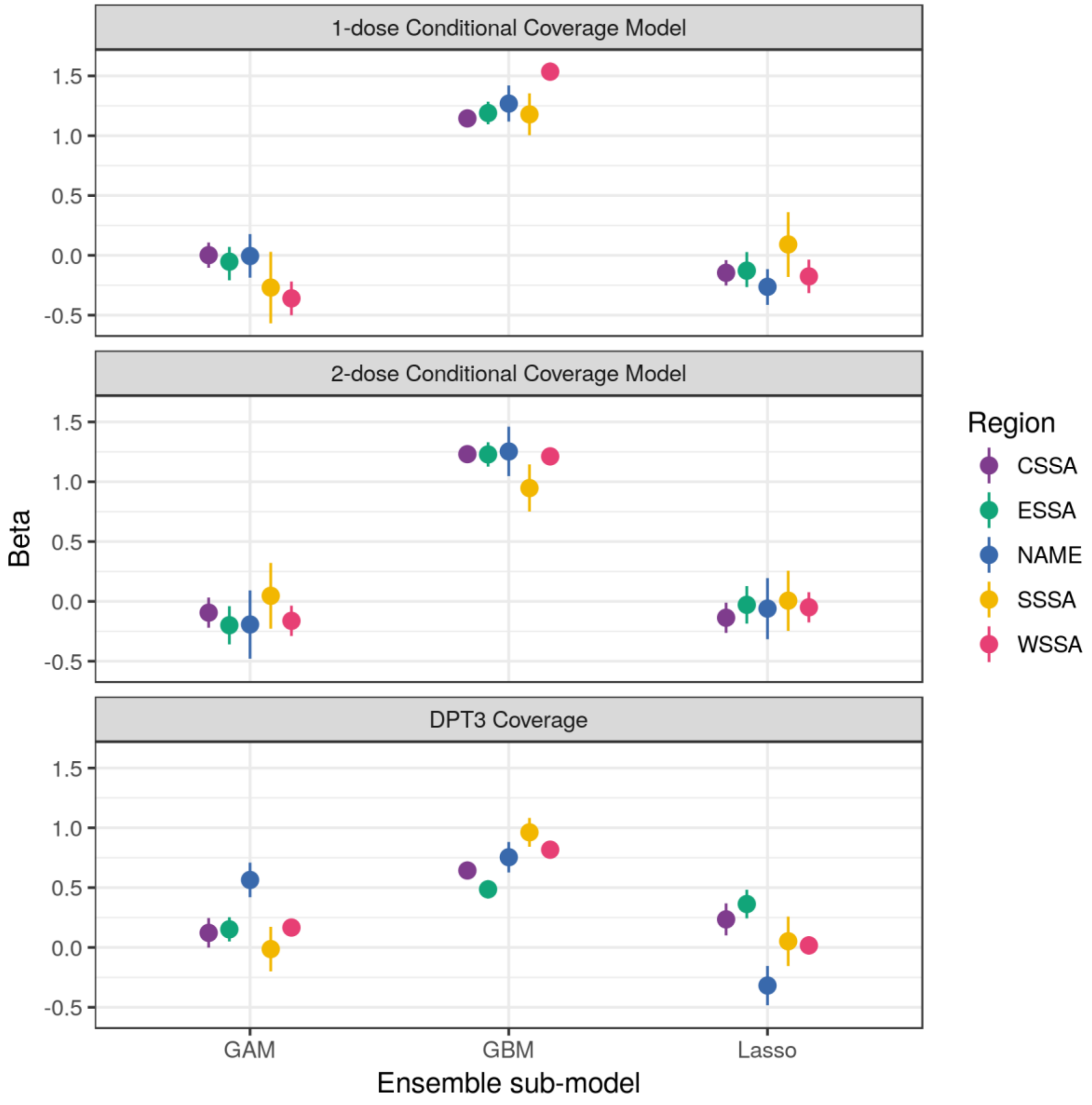

5.5 Model validation

5.5.1 Metrics of predictive validity

Spatially and temporally stratified five-fold out-of-sample cross-validation[21] was used to assess the predictive validity of estimated DPT3 coverage, DPT1 coverage, and DPT1-3 absolute dropout. Temporal folds were created by stratifying across years such that each fold contains approximately 1/5 of the data for each year. Spatial folds were created by allocating each second-level administrative unit in the modelling region to one of five folds (Supplementary Figure 17).

Each geostatistical model (DPT3 coverage and the one- and two-dose conditional coverage models) was run five times, holding out data from one of the spatiotemporal folds each time and generating a set of out-of-sample predictions from the held out data. These draw-level model outputs were then combined as described above in section 5.4.5 (Conditional ordinal regression modelling) to generate draw-level out-of-sample estimates of DPT3 and DPT1 coverage as well as relative and absolute dropout. The spatiotemporal folds were generated using the full data set, then applied uniformly to each geostatistical model in the continuation ratio ordinal regression framework. This ensured that the same data were held out for all steps in the calculation of draw-level estimates of DPT3 and DPT1 coverage and dropout.

Using these out-of-sample estimates of DPT3 coverage, DPT1 coverage, and DPT1-3 absolute dropout, multiple out-of-sample metrics of predictive validity were calculated, including mean error (ME, or bias), mean absolute error (MAE), root-mean-squared error (RMSE, which summarizes the total variance), and 95% coverage of predictive intervals (the proportion of out-of-sample observations that fall within the 95% predictive credible intervals). Each predictive metric was calculated by first simulating predictive draws using a binomial distribution. The predictive metric of interest was then calculated as a sample-size-weighted mean over national, first and second administrative levels, using boundary definitions from the FAO Global Administrative Unit Layers (GAUL)[3]. These out-of-sample results are summarized in Supplementary Figures 18-21. In addition, scatter plots of out-of-sample predictions of coverage at the second administrative level compared to observed data can be found in Supplementary Figures 22-24.

Finally, in order to explore spatial and temporal patterns of error in our results, maps of in-sample residual absolute error (predictions vs observed data) over space and time were produced for 2000, 2005, 2010, and 2016 (Supplementary Figures 25-27)

Supplementary Figure 17: Visualization of spatial holdouts at the second administrative level
Each point represents a single observation as entered into the INLA geospatial model for a single year in the West Africa region, coloured by fold number with size proportional to sample size. Transparency is used to show the weights of candidate observations generated by the areal data resampling procedure.

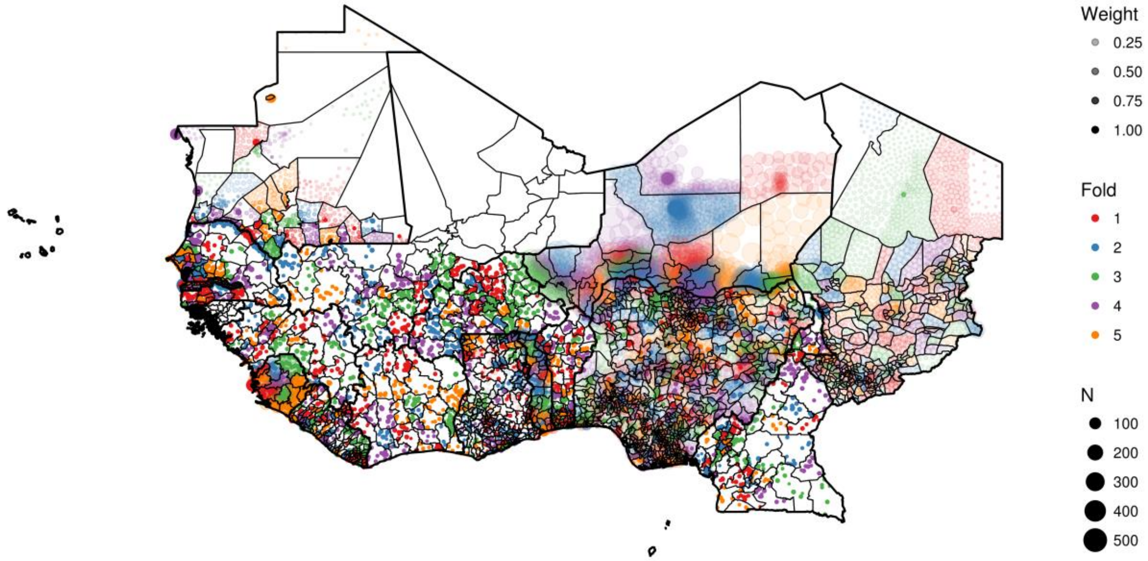

Supplementary Figure 18: Out-of-sample 95% coverage of predictive intervals.
Results are displayed for each modelled year as time series. Each panel represents level of aggregation (Country, first-level administrative unit, second-level administrative unit). Line colours represent the modelled indicator.

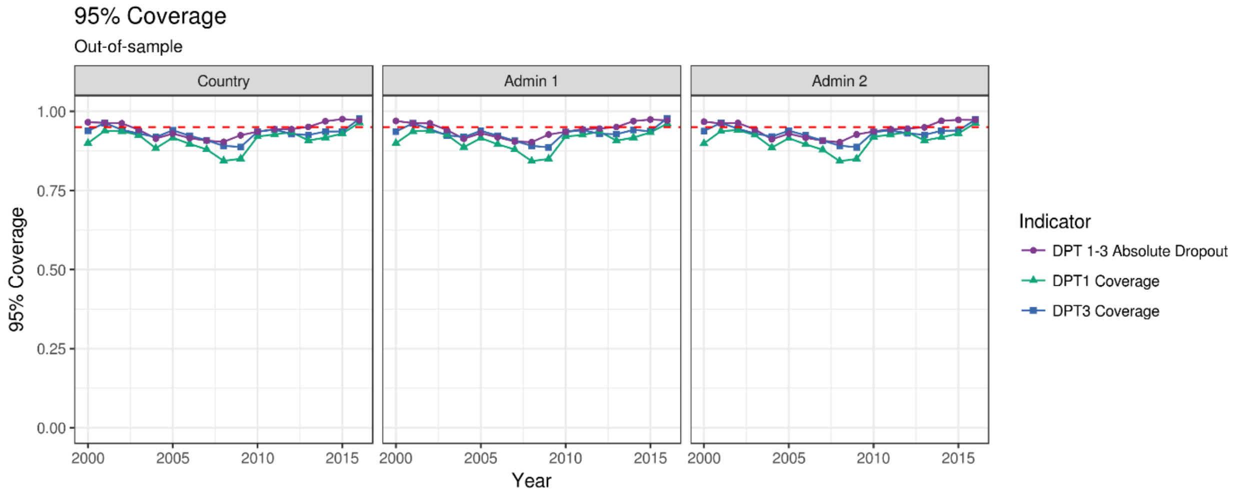

Supplementary Figure 19: Out-of-sample mean absolute error.
Results are displayed for each modelled year as time series. Each panel represents level of aggregation (Country, first-level administrative unit, second-level administrative unit). Line colours represent the modelled indicator.

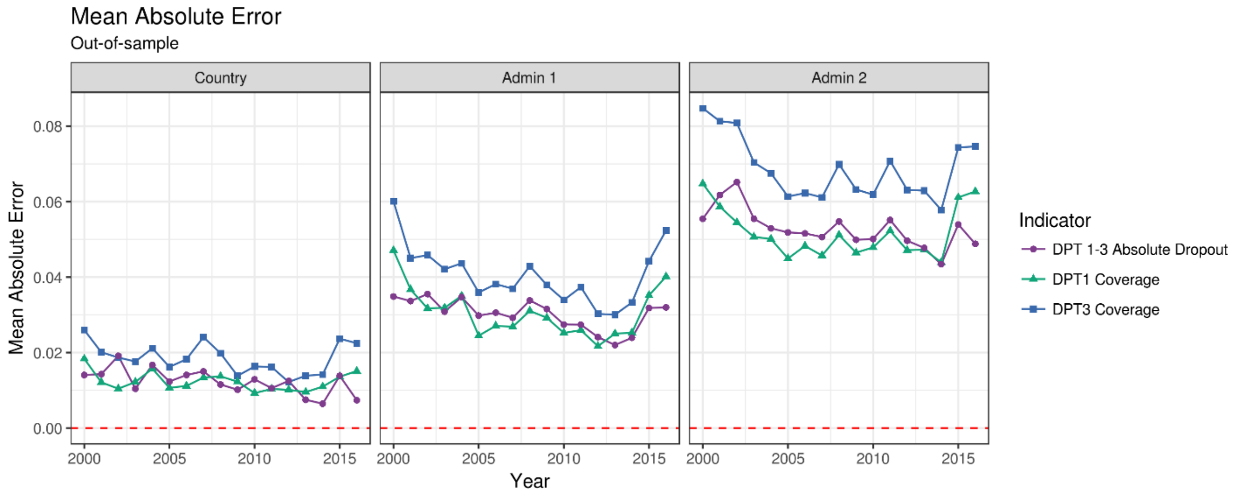

Supplementary Figure 20: Out-of-sample mean error (bias).
Results are displayed for each modelled year as time series. Each panel represents level of aggregation (Country, first-level administrative unit, second-level administrative unit). Line colours represent the modelled indicator.

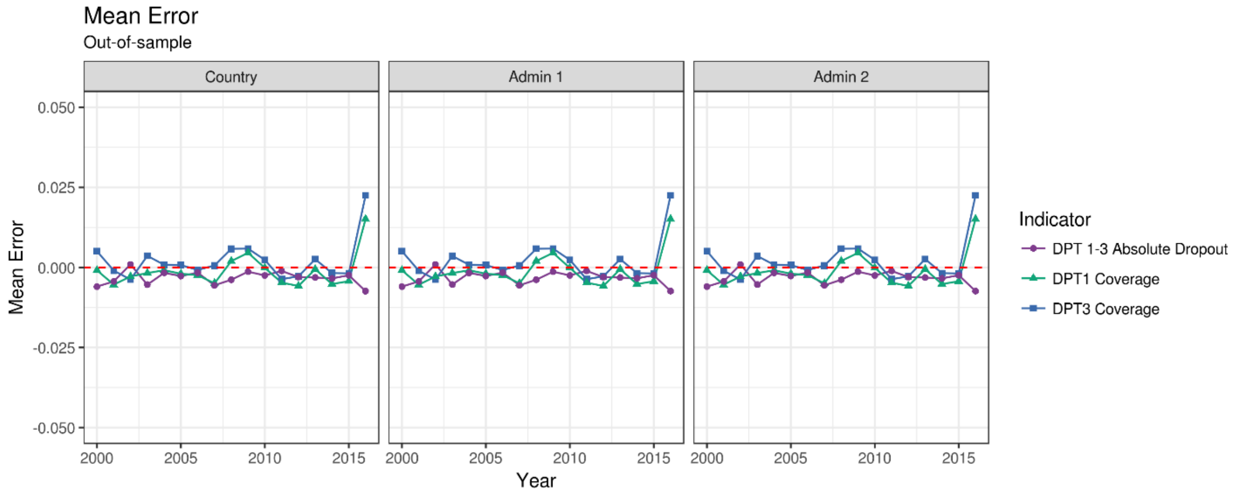

Supplementary Figure 21: Out-of-sample RMSE.
Results are displayed for each modelled year as time series. Each panel represents level of aggregation (Country, first-level administrative unit, second-level administrative unit). Line colours represent the modelled indicator.

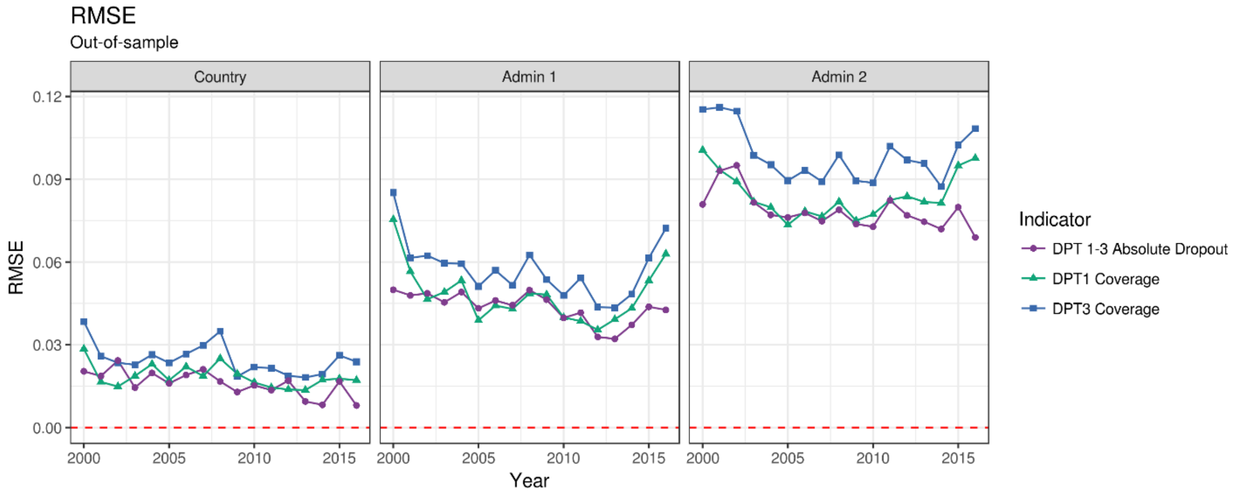

Supplementary Figure 22: Out-of-sample predictions of DPT3 coverage vs. observed data at the second administrative level

Comparison of out-of-sample predictions of DPT3 coverage, aggregated to the second administrative level with 95% predictive intervals, plotted against data observations from the same area aggregated to the second administrative level. Out of sample predictions were generated from second-administrative-level spatiotemporal holdouts.

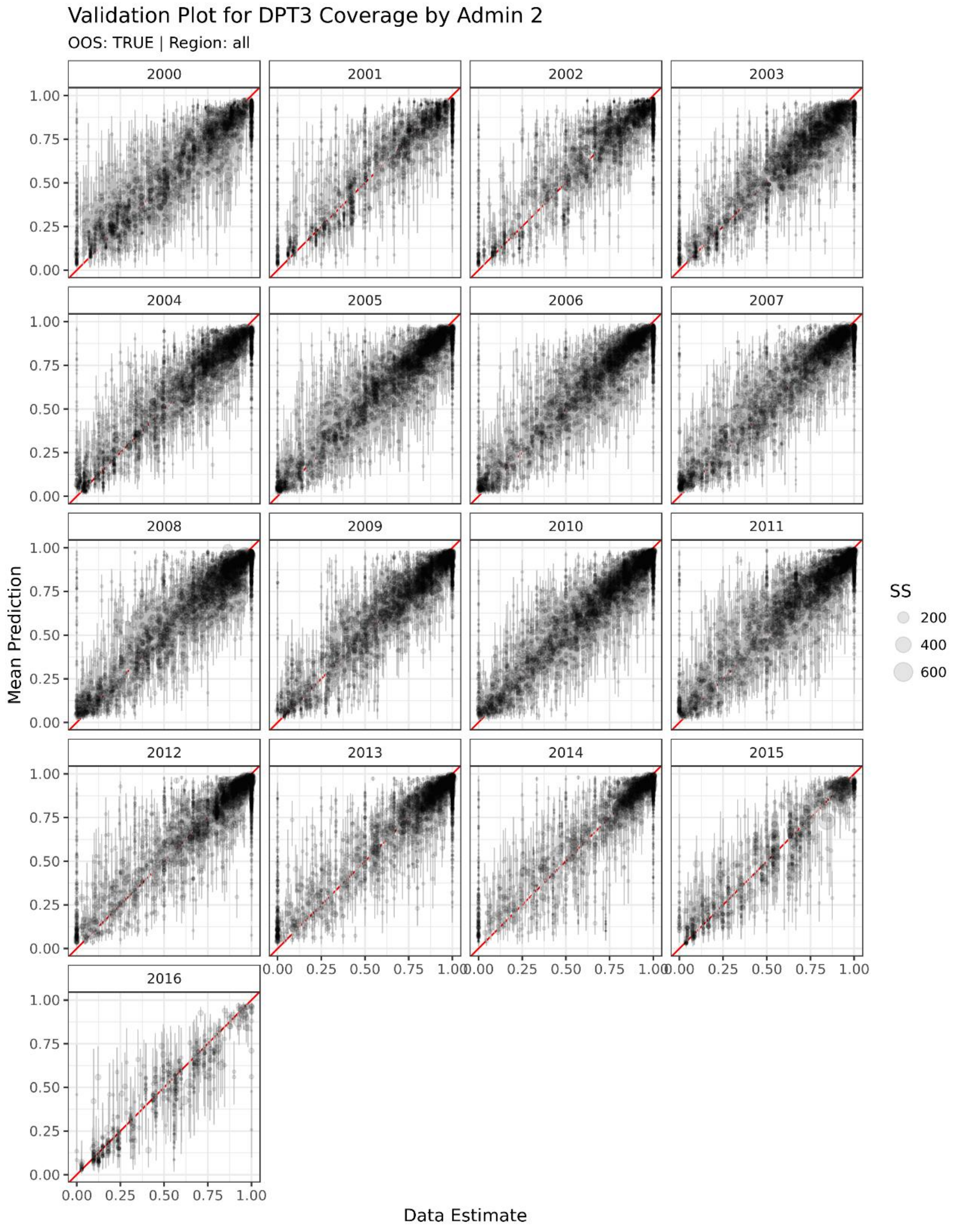

Supplementary Figure 23: Out-of-sample predictions of DPT1 coverage vs. observed data at the second administrative level

Comparison of out-of-sample predictions of DPT1 coverage, aggregated to the second administrative level with 95% predictive intervals, plotted against data observations from the same area aggregated to the second administrative level. Out of sample predictions were generated from second-administrative-level spatiotemporal holdouts.

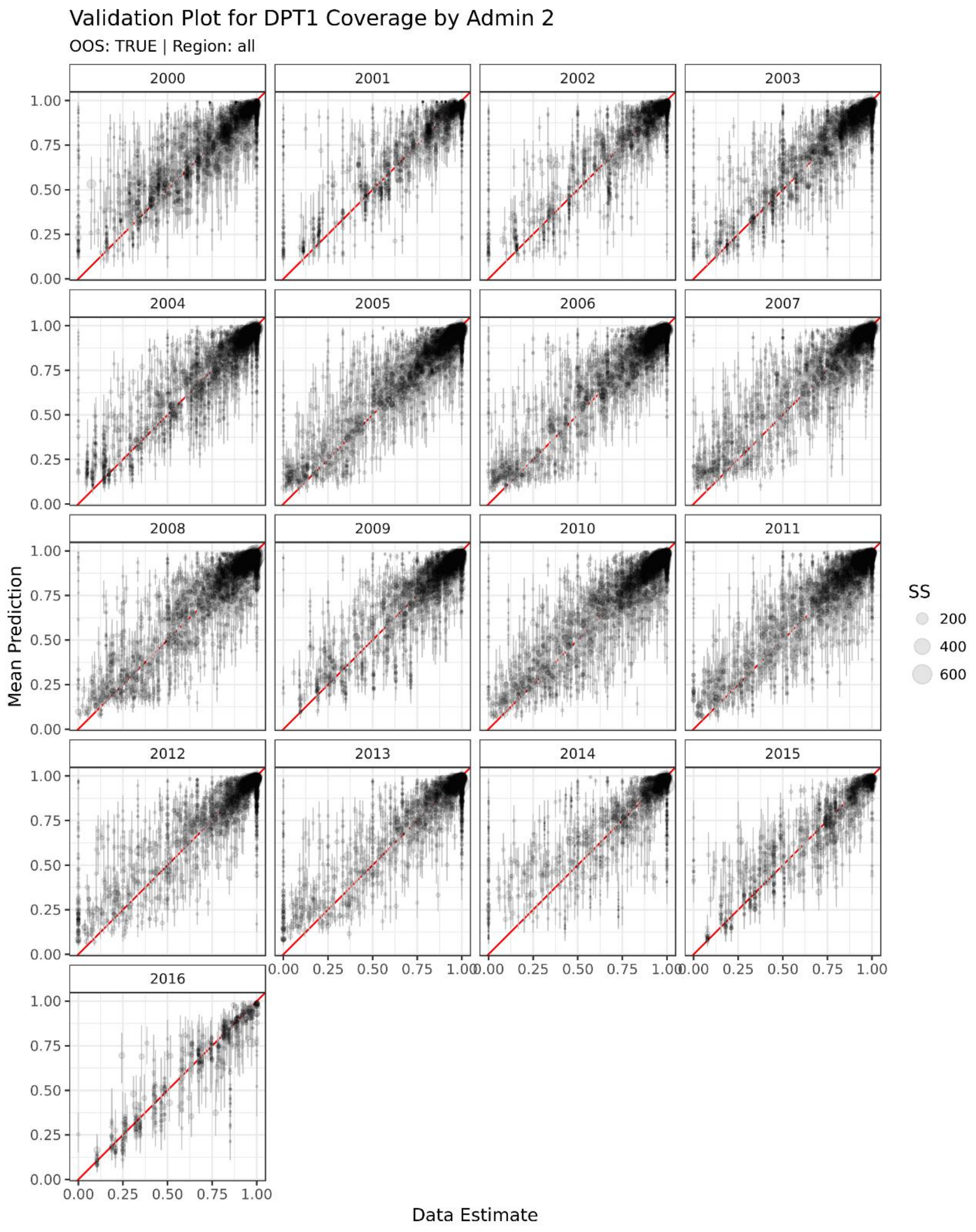

Supplementary Figure 24: Out-of-sample predictions of DPT1-3 absolute dropout vs. observed data at the second administrative level

Comparison of out-of-sample predictions of DPT1-3 absolute dropout, aggregated to the second administrative level with 95% predictive intervals, plotted against data observations from the same area aggregated to the second administrative level. Out of sample predictions were generated from second-administrative-level spatiotemporal holdouts.

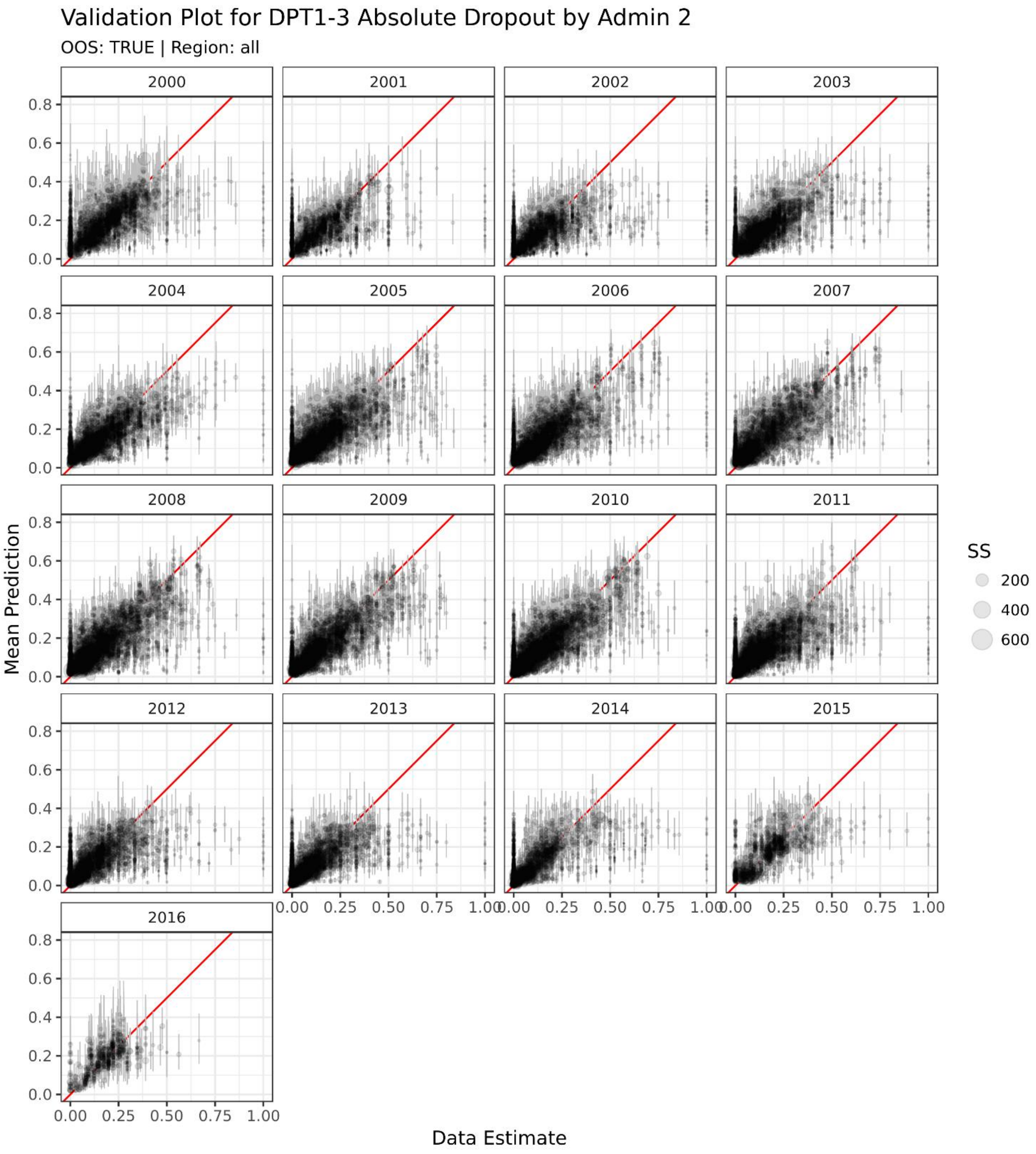

Supplementary Figure 25: Residual error maps for DPT3 coverage

Maps of residual error for DPT3 coverage for four time periods: 2000-2003, 2004-2007, 2008-2011, 2012-2016. Each point represents a single observed coverage data observation entered into the geospatial model (both precisely geopositioned observations and candidate observations generated using the resampling procedure for areal data). The size of each point represents the sample size for the observation, colour represents residual error, and transparency represents the weight of the observation. Residual error is calculated as (predicted value – observed value).

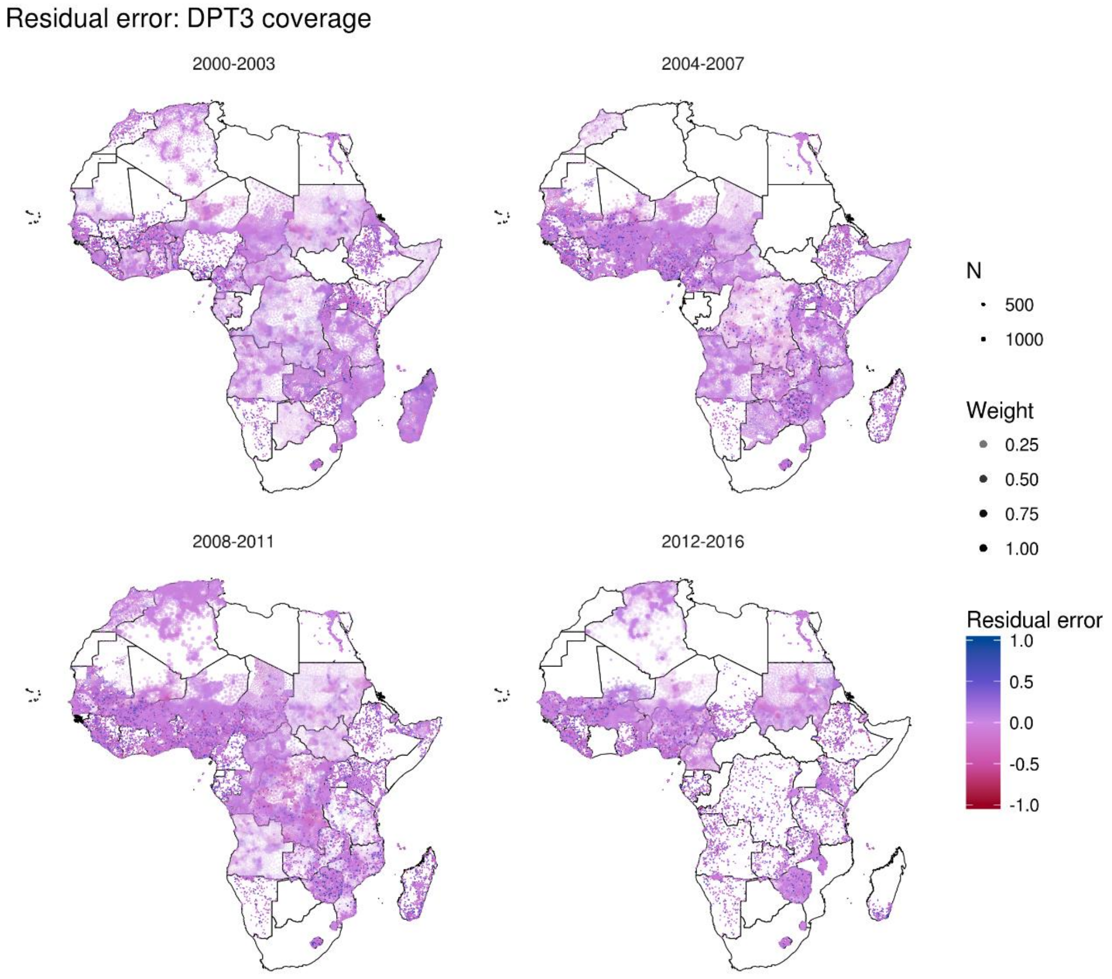

Supplementary Figure 26: Residual error maps for DPT1 coverage

Maps of residual error for DPT1 coverage for four time periods: 2000-2003, 2004-2007, 2008-2011, 2012-2016. Each point represents a single observed coverage data observation entered into the geospatial model (both precisely geopositioned observations and candidate observations generated using the resampling procedure for areal data). The size of each point represents the sample size for the observation, colour represents residual error, and transparency represents the weight of the observation. Residual error is calculated as (predicted value – observed value).

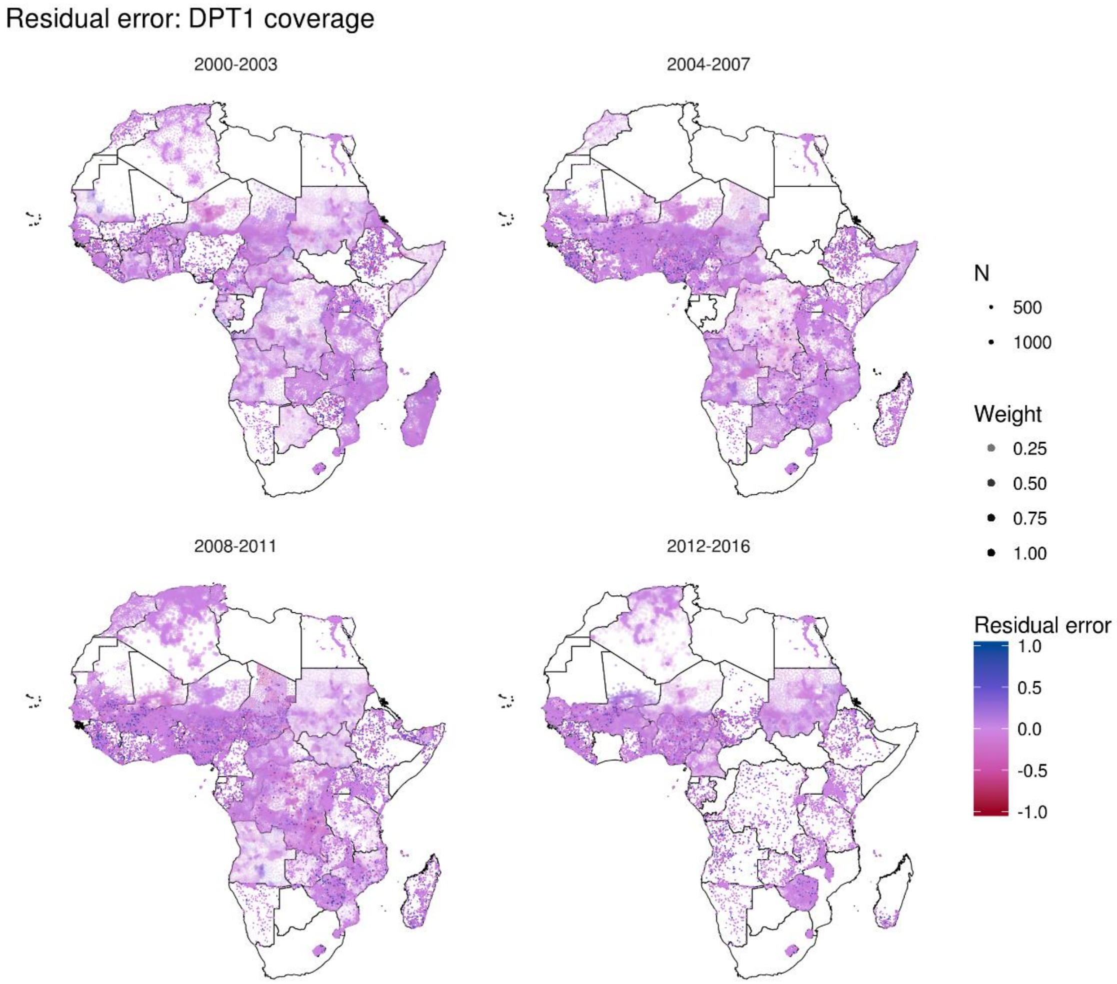

Supplementary Figure 27: Residual error maps for DPT 1-3 absolute dropout

Maps of residual error for DPT 1-3 absolute dropout for four time periods: 2000-2003, 2004-2007, 2008-2011, 2012-2016. Each point represents a single observed coverage data observation entered into the geospatial model (both precisely geopositioned observations and candidate observations generated using the resampling procedure for areal data). The size of each point represents the sample size for the observation, colour represents residual error, and transparency represents the weight of the observation. Residual error is calculated as (predicted value – observed value).

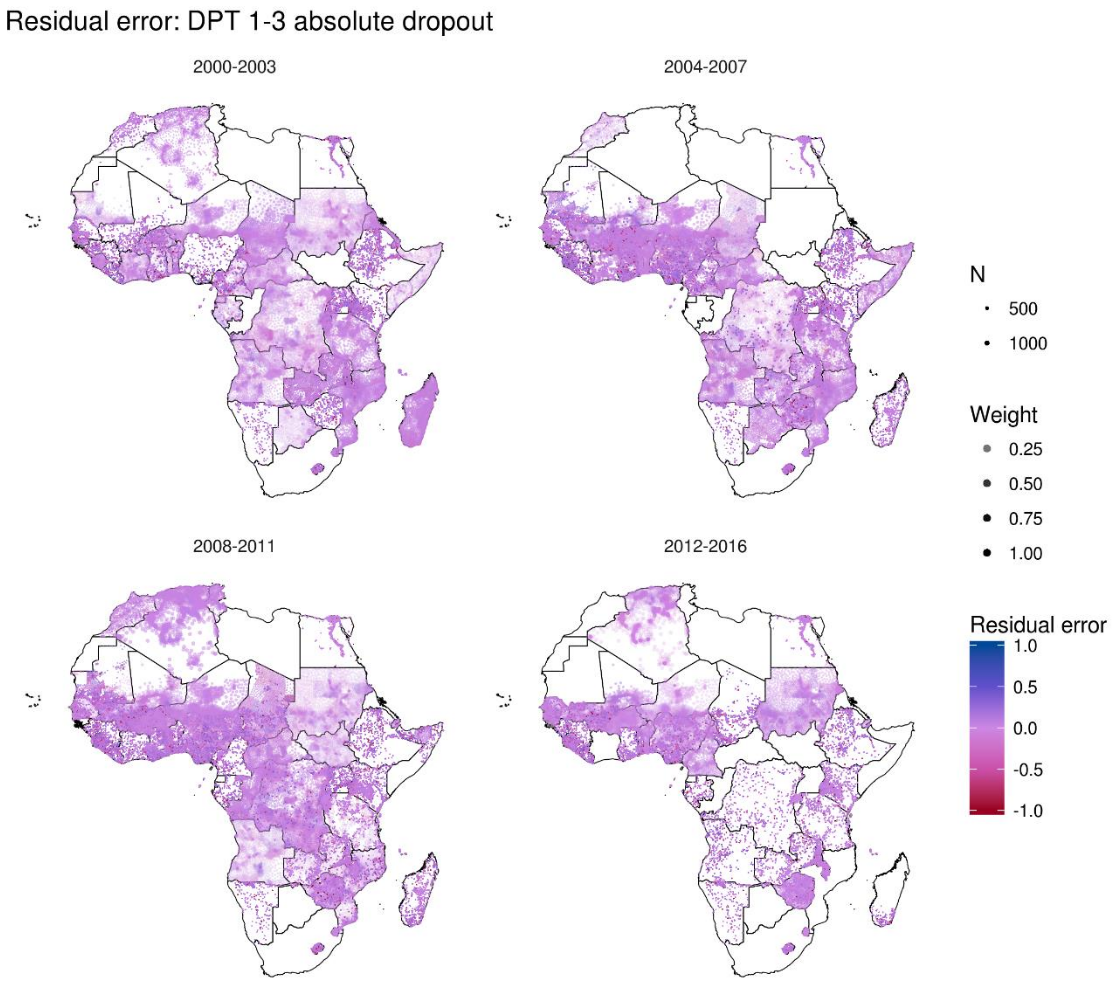

5.5.2 Model comparisons

To investigate the relative contributions of each component of the geostatistical modelling process, the above modelling process was repeated for five different combinations of covariates and spatiotemporal effects:

1) Raw covariates:
$$\text{logit}(p_i) = \beta_0 + X_i\beta_{raw} + \epsilon_{ctry_i} + \epsilon_i$$

2) Predictions from covariate ensemble modelling ("Stacking covariates"):
$$\text{logit}(p_i) = \beta_0 + X_i\beta_{stack} + \epsilon_{ctry_i} + \epsilon_i$$

3) Gaussian Process (GP):
$$\text{logit}(p_i) = \beta_0 + \epsilon_{GP_i} + \epsilon_{ctry_i} + \epsilon_i$$

4) Raw covariates + GP:
$$\text{logit}(p_i) = \beta_0 + X_i\beta_{raw} + \epsilon_{GP_i} + \epsilon_{ctry_i} + \epsilon_i$$

5) Stacking covariates + GP:
$$\text{logit}(p_i) = \beta_0 + X_i\beta_{stack} + \epsilon_{GP_i} + \epsilon_{ctry_i} + \epsilon_i$$

Models were compared using out-of-sample metrics of predictive accuracy using the five-fold cross-validation strategy described above, including RMSE, 95% coverage, and bias, aggregated to the second administrative level (as second-level administrative units were used to generate folds for cross validation). Supplementary Figures 28-30 show the results of this comparison. Across indicators, the combination of ensemble-modelled covariates ("stacking covariates") and the Gaussian process generally improves out-of-sample 95% coverage and produces equivalent or improved RMSE and bias compared to alternative models. Maps of estimated DPT3 coverage in 2016 for each of these comparison models can be found in Supplementary Figure 31.

Supplementary Figure 28: Out-of-sample model comparisons for DPT3 coverage

"Raw Covs" represents the INLA model fit with linear terms on all raw covariates, but without ensemble modelling or the space-time Gaussian process; "Stacked covs" corresponds to an INLA fit with the prediction surfaces obtained from covariate ensemble modelling but no space-time Gaussian process; "GP" is fit only with the space-time Gaussian process; "Raw + GP" is fit with linear terms on all raw covariates and the space-time Gaussian process; "Stacked + GP" is fit with the prediction surfaces from covariate ensemble modelling and the space-time Gaussian process and represents the model used for reporting of results in this work. Red lines and text indicate the region of the plot that indicates best performance (low bias, low RMSE, and 95% coverage equal to 95%).

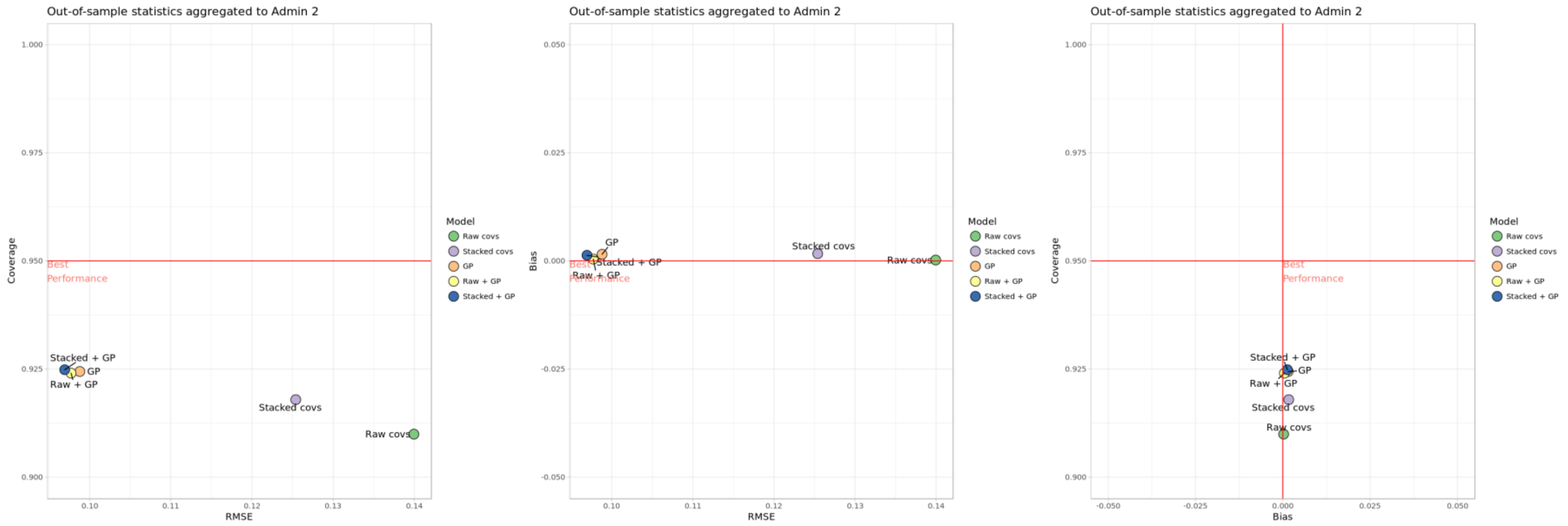

Supplementary Figure 29: Out-of-sample model comparisons for DPT1 coverage
“Raw Covs” represents the INLA model fit with linear terms on all raw covariates, but without ensemble modelling or the space-time Gaussian process; “Stacked covs” corresponds to an INLA fit with the prediction surfaces obtained from covariate ensemble modelling but no space-time Gaussian process; “GP” is fit only with the space-time Gaussian process; “Raw + GP” is fit with linear terms on all raw covariates and the space-time Gaussian process; “Stacked + GP” is fit with the prediction surfaces from covariate ensemble modelling and the space-time Gaussian process and represents the model used for reporting of results in this work. Red lines and text indicate the region of the plot that indicates best performance (low bias, low RMSE, and 95% coverage equal to 95%).

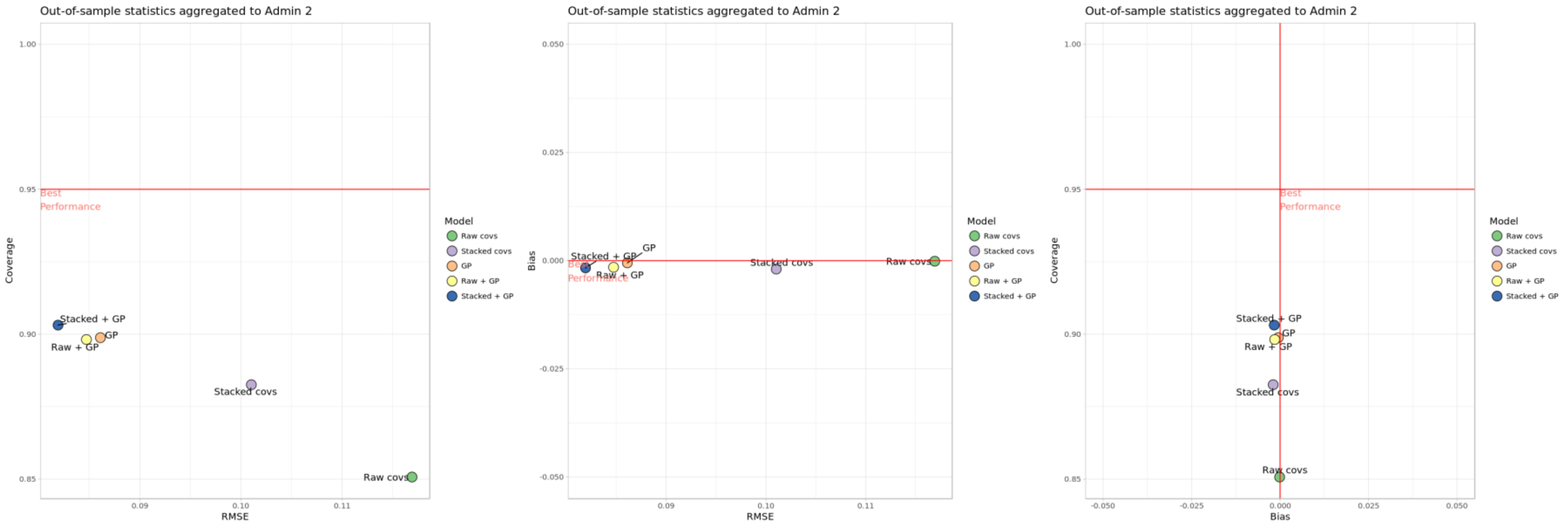

Supplementary Figure 30: Out-of-sample model comparisons for DPT1-3 absolute dropout comparisons
"Raw Covs" represents the INLA model fit with linear terms on all raw covariates, but without ensemble modelling or the space-time Gaussian process; "Stacked covs" corresponds to an INLA fit with the prediction surfaces obtained from covariate ensemble modelling but no space-time Gaussian process; "GP" is fit only with the space-time Gaussian process; "Raw + GP" is fit with linear terms on all raw covariates and the space-time Gaussian process; "Stacked + GP" is fit with the prediction surfaces from covariate ensemble modelling and the space-time Gaussian process and represents the model used for reporting of results in this work. Red lines and text indicate the region of the plot that indicates best performance (low bias, low RMSE, and 95% coverage equal to 95%).

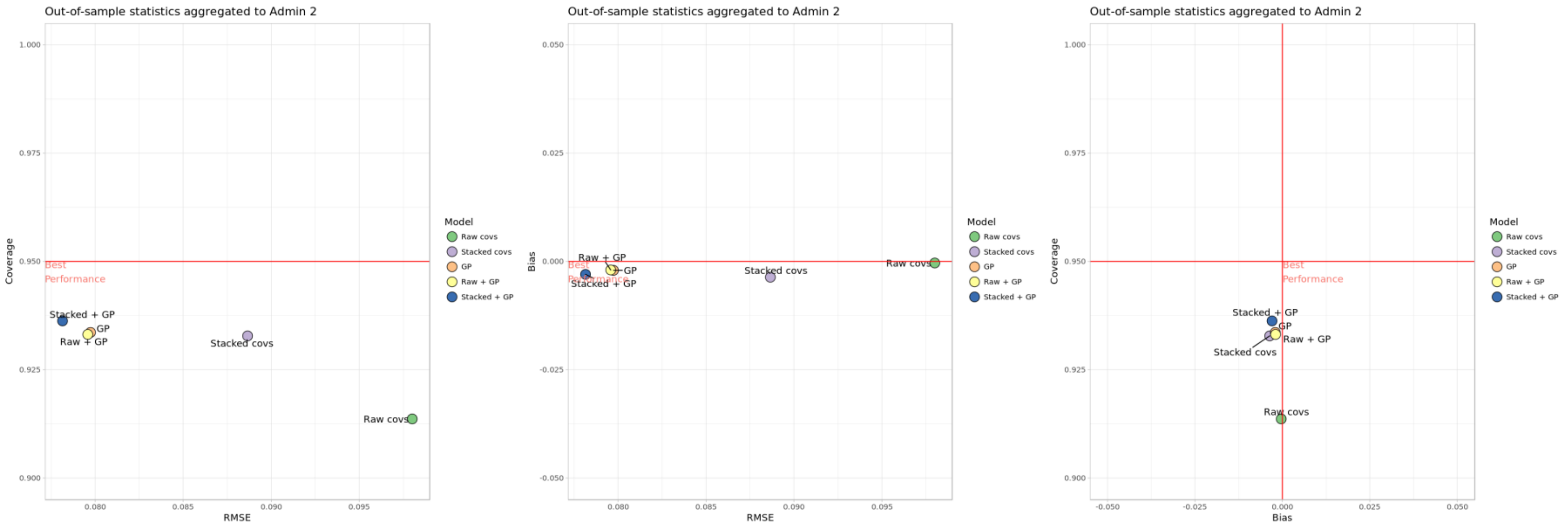

Supplementary Figure 31: Estimated DPT3 coverage for each comparison model, 2016.
Each map represents estimated DPT3 coverage (prior to calibration to national-level estimates for GBD) for 2016 at a 5x5 km spatial resolution for one of the five comparison models: a) Gaussian process alone; b) Raw covariates alone; c) Ensemble-modelled covariates alone; d) Raw covariates with Gaussian process; e) Ensemble-modelled covariates with Gaussian process (the model used for reporting estimates in this analysis).

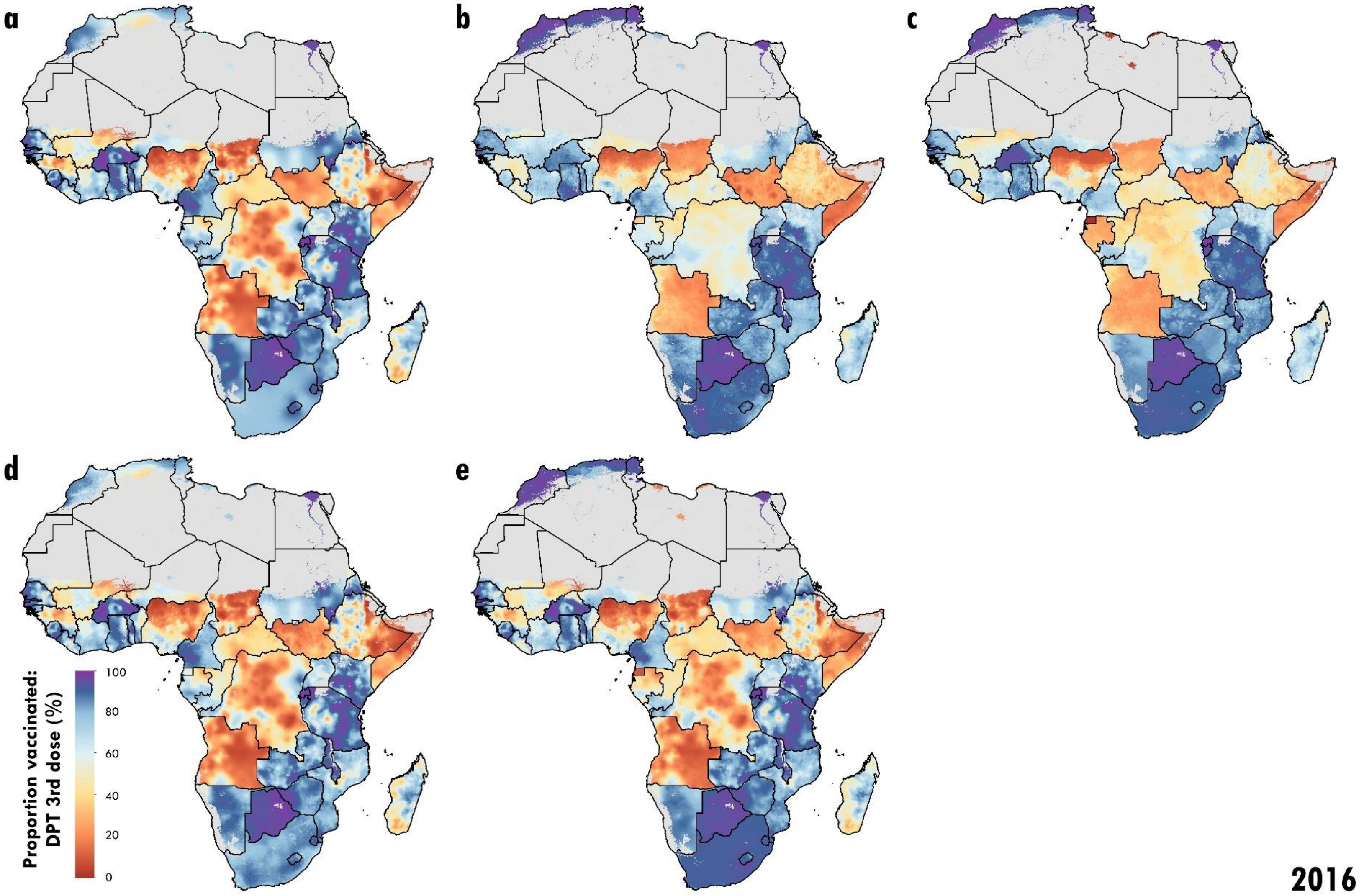

5.6 Post-estimation calibration to national estimates from the Global Burden of Disease Study

To ensure consistency between these geospatial estimates and national-level estimates of DPT coverage from the Global Burden of Disease (GBD) study, and to leverage additional national-level sources of data, a post-hoc calibration of estimates for each of the 1,000 candidate maps was performed. The effect of this calibration is to preserve relative spatial variation while ensuring that population-weighted averages of the 5x5 km maps at the national level are equivalent to GBD estimates.

5.6.1 Production of GBD national coverage estimates

The Global Burden of Disease (GBD) project produces estimates of national and subnational DTP3 immunization coverage, which are used as covariates in the estimation of multiple disease processes and burdens. For GBD 2017, estimates are produced for 195 countries and 695 subnational administrative units from 1980 through 2017. This indicator represents the proportion of surviving 12- to 23-month-olds in a given cohort who have received at least three doses of the diphtheria-tetanus-pertussis (DTP) vaccine in a given year. These methods were additionally adapted to analogously estimate DPT1 coverage, from which DPT1-3 dropout can be calculated.

Input Data

These estimates leverage data from household surveys as well as administrative reports of immunization coverage. Surveys which provide unit record data or summary reports on immunization coverage were included. Major multi-country survey programs used in the analysis include the Demographic and Health Surveys (DHS), Multiple Indicator Cluster Surveys (MICS), Reproductive Health Surveys (RHS), and Living Standards Measurement Study (LSMS) surveys. A comprehensive search of the Global Health Data Exchange (GHDx), as well as targeted internet searches and review of Ministry of Health websites, were also used to identify national surveys and other multi-country survey programs. Excluded subnationally-resolved surveys and differences between the data set used for geospatial estimation of DPT coverage in this study and subnationally-resolved surveys used in the GBD estimation process are summarized in Supplementary Table 2 and Supplementary Table 3.

Survey weights were applied based on survey sampling frames whenever they were available to generate weighted national estimates of vaccination coverage accompanied by estimates of standard error (SE). Estimates of SE, as well as sample sizes, were used to calculate uncertainty, as described below. Any point estimates with sample sizes less than 50 were reviewed to ensure that were not substantive outliers and would not otherwise have an undue influence on coverage estimates.

Inclusion of administrative data and administrative bias adjustment

Administrative estimates of immunization coverage were obtained from the Joint Reporting Form (JRF), through which the World Health Organization (WHO) and UNICEF collate annual immunization coverage reported by UN member states.[22] These estimates are released each July to include administrative coverage from the previous year.

The GBD process then implements a vaccine-specific bias adjustment process to account for bias in administrative reports of immunization coverage in the JRF. Given that the magnitude, direction, and cause of such biases,[23,24] a vaccine-specific, time-varying, all-location bias correction factor was used. To accomplish this correction, the ratio between survey coverage (where available) and matched administrative coverage was modelled in the spatiotemporal Gaussian process regression (ST-GPR) described below using the Socio-Demographic Index (SDI)[11] as a predictor. The inverses of these modelled ratios were then applied across the entire administrative data time series for all countries before inputting the data in the model. The adjustment often, but not always, suggested over-reporting of coverage in the JRF.

Coverage trend estimation

The GBD study uses a spatiotemporal Gaussian process regression (ST-GPR) to synthesize point estimates from multiple data sources and derive a complete time series for each vaccine. This method has been used extensively within the GBD and related studies, and accounts for uncertainty pertaining to each point estimate while borrowing strength across geographic space and time.[25–27]

Briefly, the Gaussian process is assumed to be defined by a mean function *m*(•) and covariance function *Cov*(•). The mean function $m_c(t)$ is estimated using a two-step approach:

$$m_c(t) = X\beta + h(r_{c,t}) \ ,$$

where $X\beta$ is a linear model and $h(r_{c,t})$ is a smoothing function for the residuals; and $r_{c,t}$ is derived from the linear model. The following linear model was used:

$$logit(P_{c,t}) = \beta_0 + \ \beta_1 \mathrm{LDI}_{\mathrm{c,t}} + \ \beta_2 \mathrm{war}_{\mathrm{c,t}} + \ \alpha_c + \ \gamma_{R[c]} + \ \omega_{\mathrm{SR[c]}} \ + \ \varepsilon_{c,t} \ ,$$

where $P_{c,t}$ is vaccination coverage for country c year t; $LDI_{c,t}$ is lagged distributed income level for country c and year t; $\mathrm{war}_{\mathrm{c,t}}$ is the mortality rate due to conflict, used as a proxy for vaccine access and stockouts for country c year t; α_c, $\gamma_{R[c]}$, and $\omega_{\mathrm{SR[c]}}$ are country, region, and super-region random intercepts, respectively. These estimates were then modelled through the space-time and Gaussian process regression steps.

Random draws of 1,000 samples were taken from model distributions for every country for a given vaccine. Ninety-five percent uncertainty intervals were calculated by taking the ordinal 25 and 975th draws from the sample distribution.

5.6.2 Calibration of geospatial estimates to GBD national coverage estimates

Using these national-level coverage estimates, the calibration method applied to the geospatial estimates generally followed previous methods used in geospatial estimation of under-5 mortality, education, and child growth failure.[5,7] While this previous work performed calibration in untransformed space, here calibration was performed in logit space in order to ensure that all predictions remained between 0% and 100%. For each country i and year j, mean national-level estimates of vaccine coverage were obtained from the Global Burden of Disease study (C_{GBD}) and calculated as population-weighted averages from 5x5 km model-based geostatistical estimates (C_{MBG}). A country-year specific calibration factor $k_{i,j}$ was then calculated such that

$$\mathrm{logit}(C_{GBD,ij}) = \mathrm{logit}(C_{MBG,ij}) + k_{i,j}$$

These calibration factors were then applied to each 5x5 km location and draw within the country year, yielding a calibrated set of candidate maps.

This calibration process was performed on draw-level estimates of two vaccine coverage quantities generated using the continuation ratio ordinal regression framework described above: DPT3 coverage ($p(d \geq 3)$, where d is equal to the number of doses received) and the conditional probability of incomplete vaccination given either incomplete or no vaccination ($p(1 \leq d \leq 2 \mid d \leq 2)$). These calibrated probabilities were then combined arithmetically to yield calibrated estimates of DPT1 coverage, DPT3 coverage, and DPT 1-3 dropout. This conditional calibration process ensured that this calibration process preserved the internal consistency of estimates produced by the continuation ratio ordinal regression model – that is, that all probabilities of vaccine status sum to 1 at the draw level. Supplementary Figure 32 shows an example of the results of this process for a single 5x5 km location and year, where each point represents a single draw.

Supplementary Figure 32: Ternary plot of example pixel-level calibration to GBD estimates

Left: pre-calibration estimates; right: post-calibration estimates. Each point represents a single draw's position in ternary space. In this example, estimates of DPT3 coverage were similar for both GBD and geospatial estimates, although GBD estimated relatively more children receiving 1 or 2 doses, while the geostatistical process estimated more children receiving 0 doses. Note that this process preserves the relative positions of each point in ternary space.

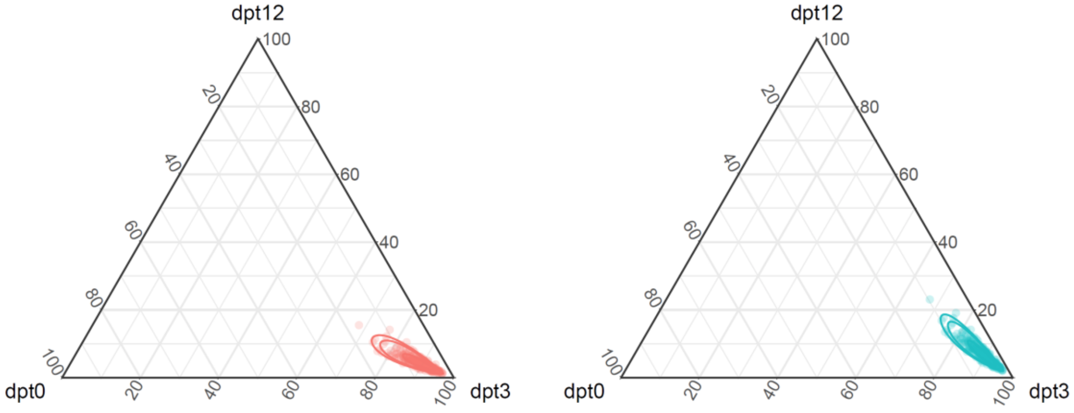

6.0 Comparison of results to existing estimates

6.1 Comparison of pre-calibration geospatial model to GBD estimates

In order to compare the pre-calibration estimates produced by the geospatial modelling process to GBD estimates produced using space-time Gaussian process regression, scatter plots are provided in Supplementary Figures 33-35. Each point represents an estimate of the given indicator for a single country and year, accompanied by 95% credible intervals. Points with a difference of > 25 percentage points between MBG and GBD estimates are labelled with country (ISO3 code) and year. The majority of these largest discrepancies occur in countries where no subnationally resolved data was available for inclusion in the geospatial model, indicating the degree to which the geospatial estimates in these countries are informed by the national-level GBD estimates (and highlighting the benefit of calibration to national-level estimates). Note that in addition to survey data GBD models leverage bias-corrected administrative data, and that the survey sources utilized by the MBG and GBD models differ (See Supplementary Table 3).

Supplementary Figure 33: Comparison of national-level estimates of DPT3 coverage from model-based geostatistics (MBG) and the Global Burden of Disease study (GBD).

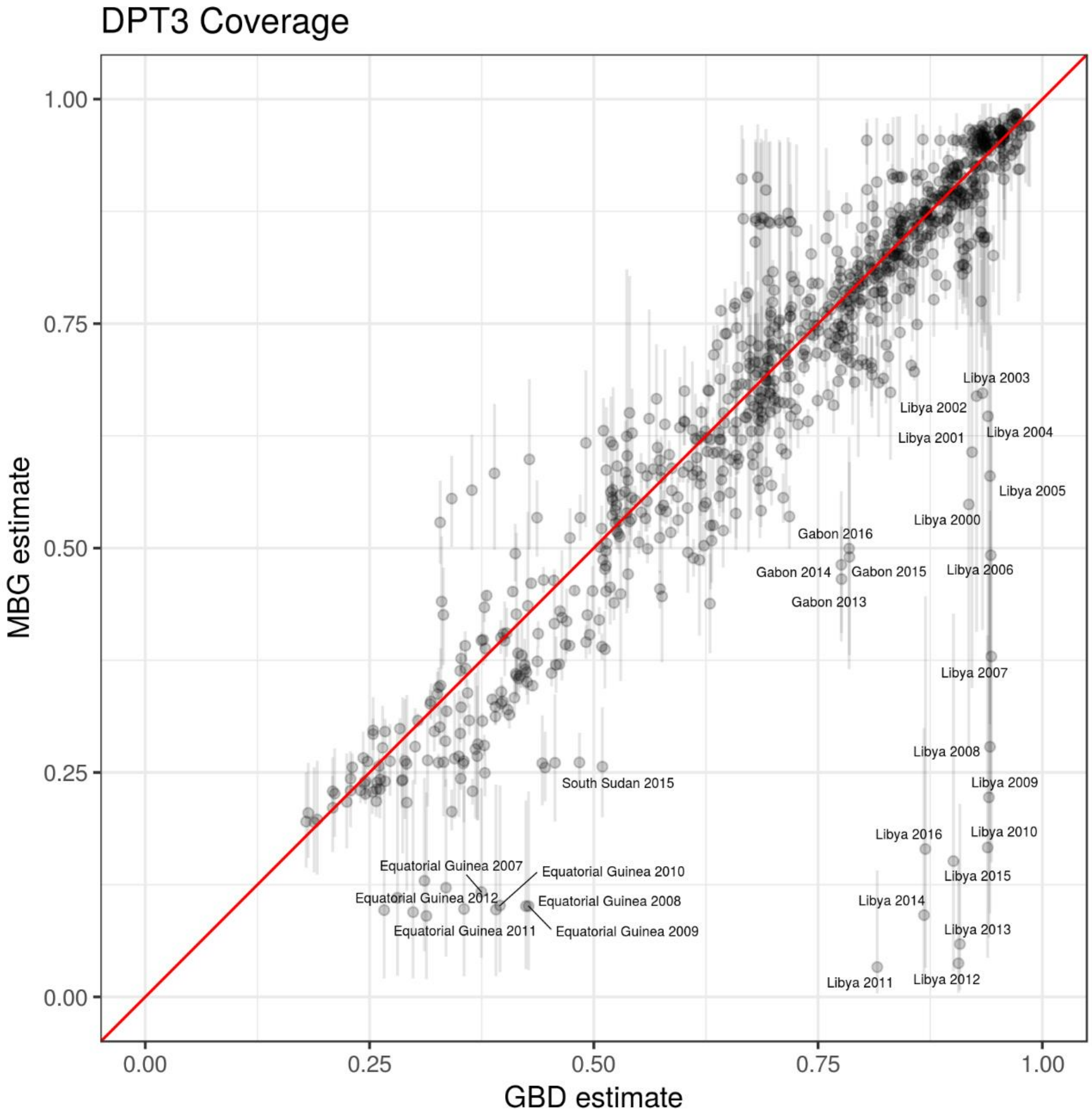

Supplementary Figure 34: Comparison of national-level estimates of DPT1 coverage from model-based geostatistics (MBG) and the Global Burden of Disease study (GBD).

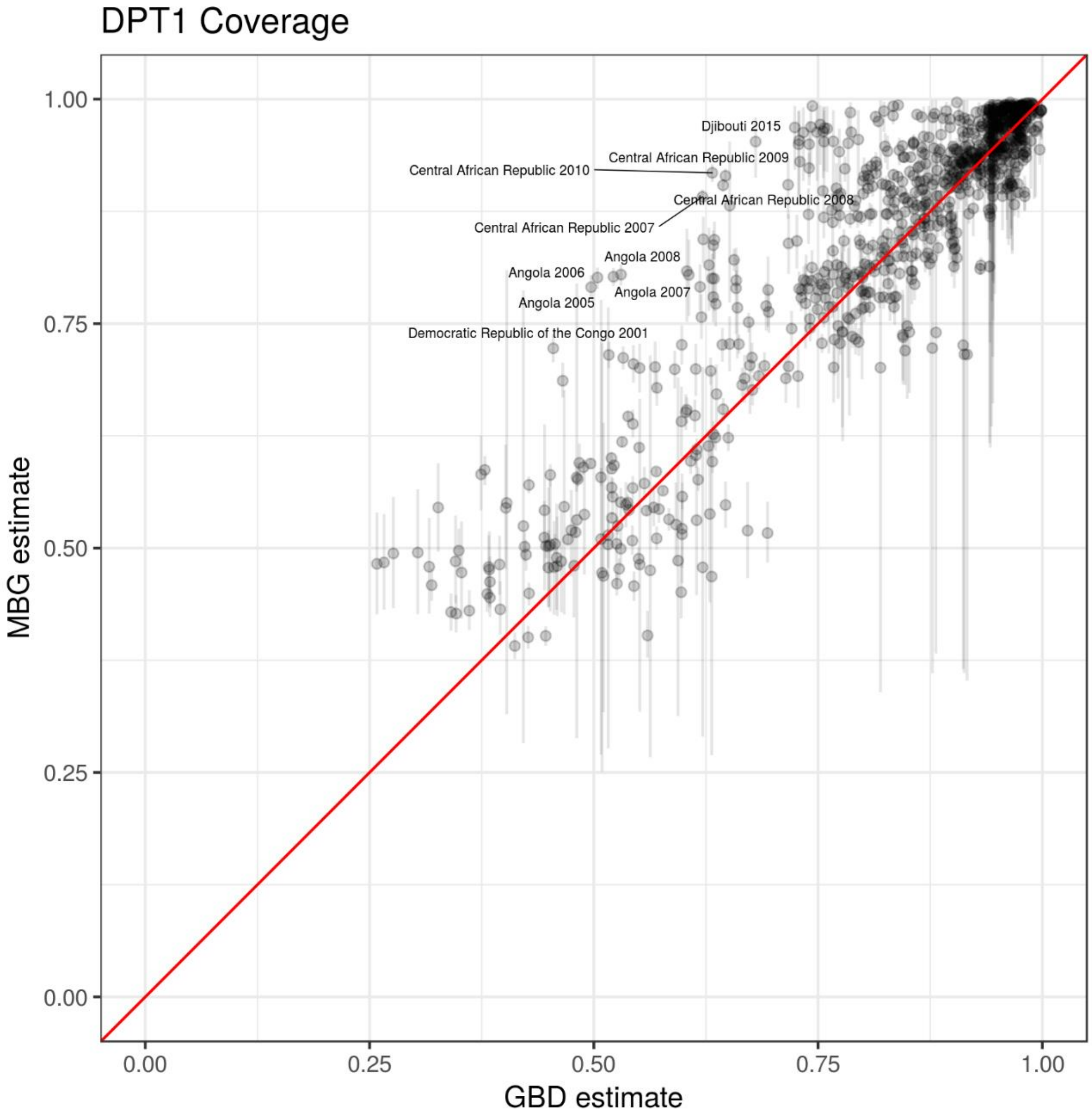

Supplementary Figure 35: Comparison of national-level estimates of DPT1-3 absolute dropout from model-based geostatistics (MBG) and the Global Burden of Disease study (GBD).

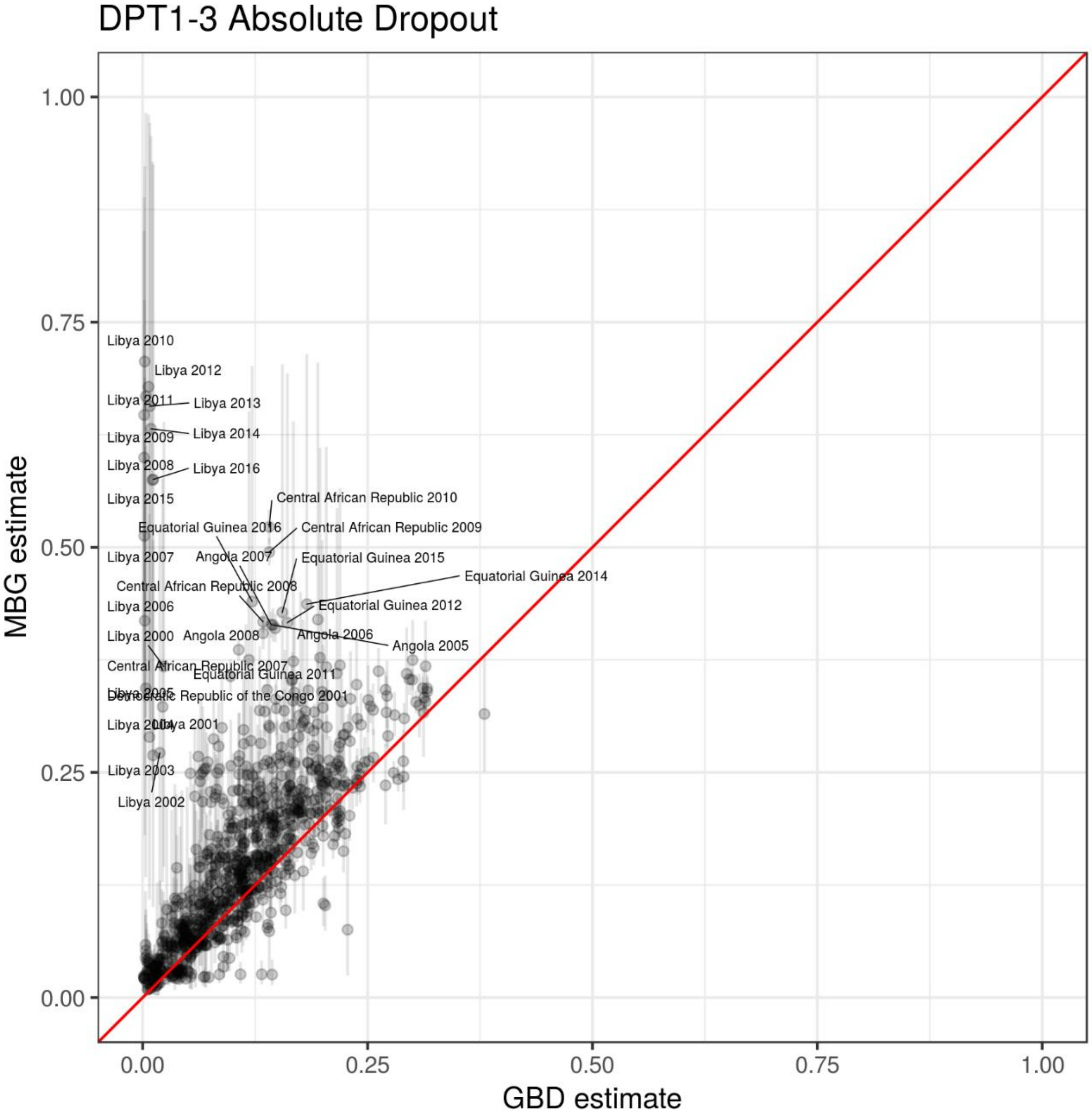

6.2 Comparison to DHS estimates

Supplementary Figures 36-38 compare estimates of DPT coverage in 12-23 month olds produced by the model-based geostatistical methods used in this study to corresponding estimates from DHS survey data, aggregated to the first administrative level. Each point in the graph represents a single administrative and DHS survey. MBG estimates are plotted with accompanying 95% certainty intervals, and separate graphs are provided for both MBG estimates both before (“uncalibrated”) and after (“calibrated”) calibration to GBD estimates.

Supplementary Figure 36: Comparison of calibrated model-based geostatistical (MBG) and DHS estimates of DPT3 coverage at the first administrative level.

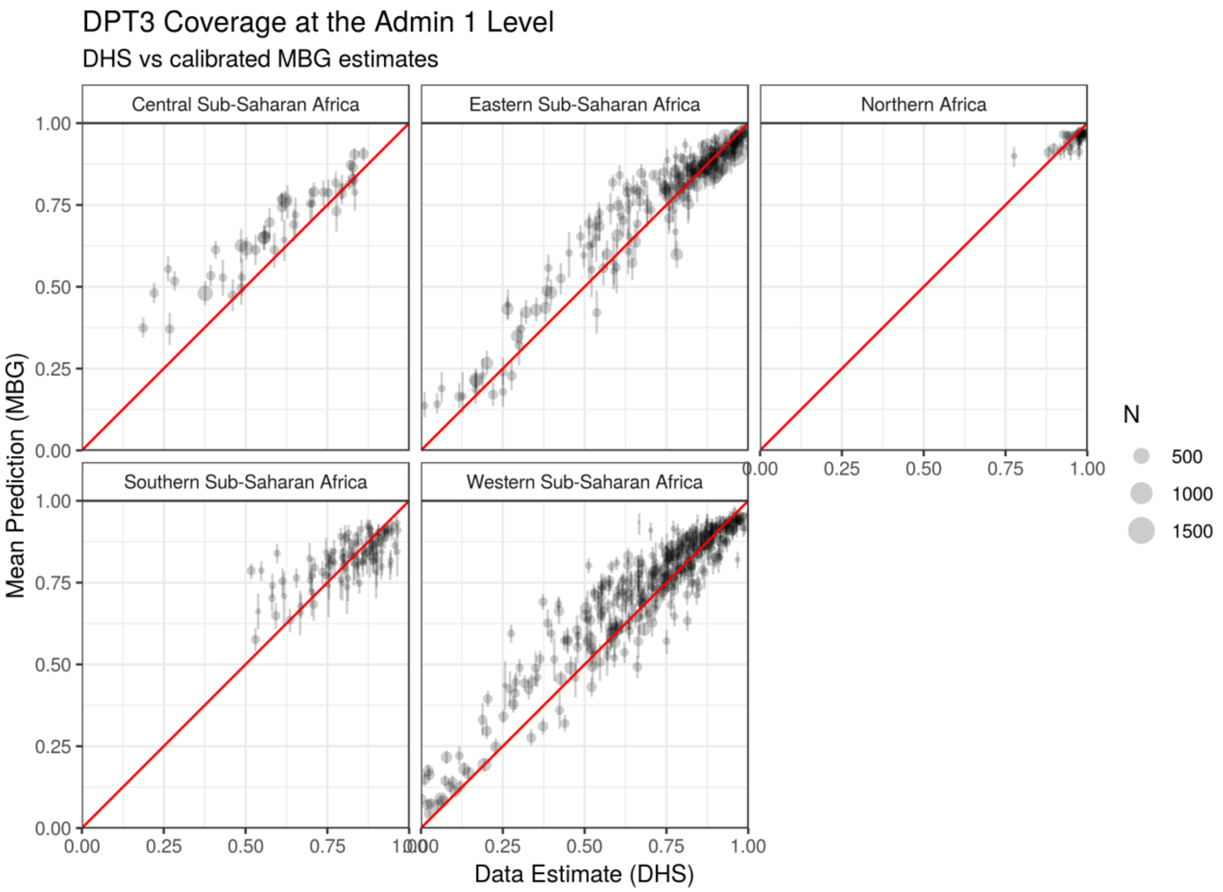

Supplementary Figure 37: Comparison of calibrated model-based geostatistical (MBG) and DHS estimates of DPT1 coverage at the first administrative level.

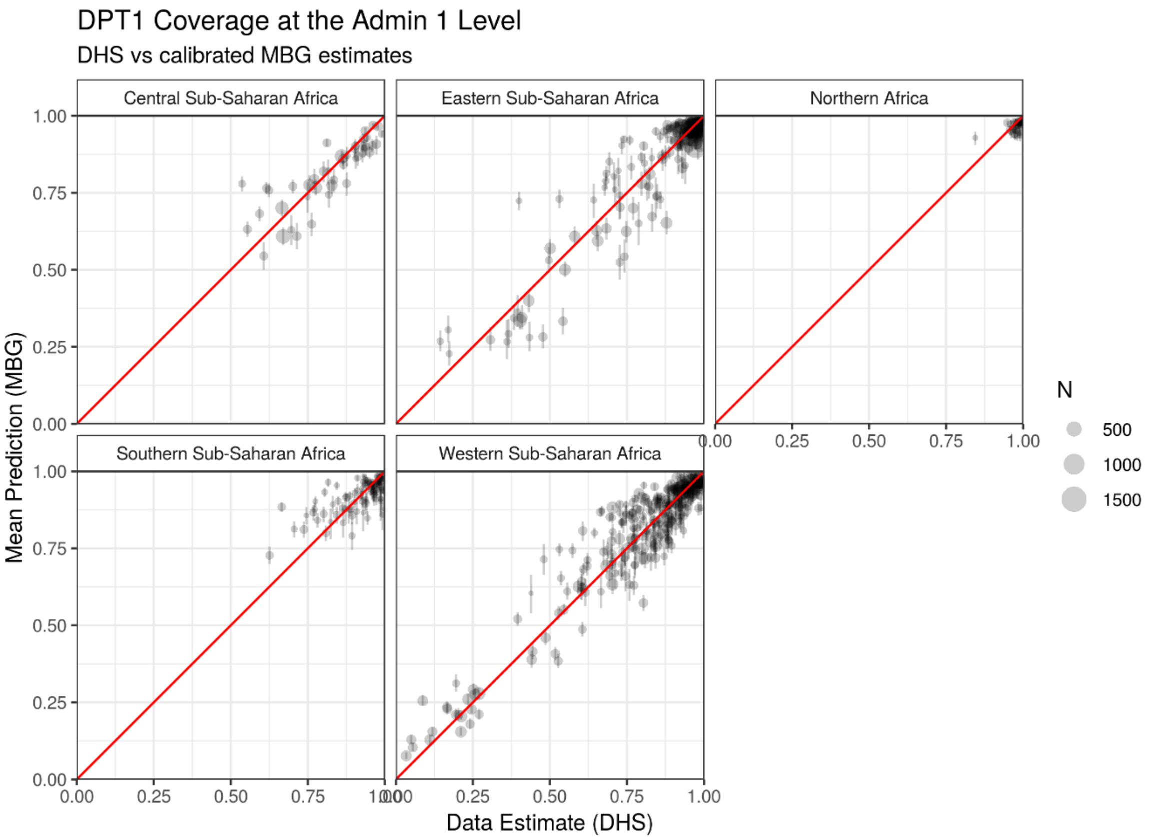

Supplementary Figure 38: Comparison of calibrated model-based geostatistical (MBG) and DHS estimates of DPT 1-3 absolute dropout at the first administrative level.

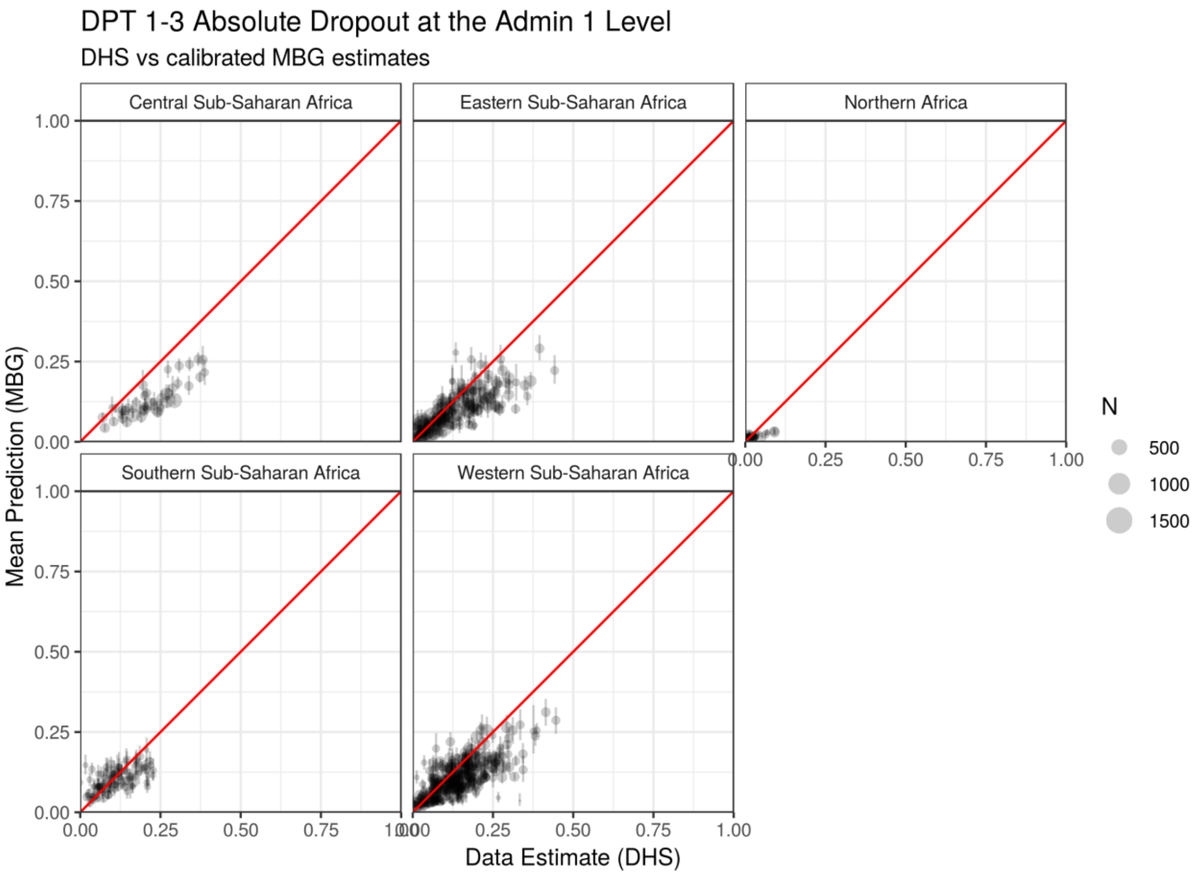

6.3 Comparison to Gavi Full Country Evaluations Project small area estimates

Supplementary Figures 39-43 compare estimates produced by the model-based geostatistical methods used in this study with the small area estimates of DPT3 coverage for varying administrative levels between 2000 and 2015 from the Gavi Full Country Evaluations project.[28] Each point represents a single administrative unit and year.

Supplementary Figure 39: Comparison of MBG and Gavi FCE second-administrative-level estimates of DPT3 coverage for Cameroon, 2000-2015.

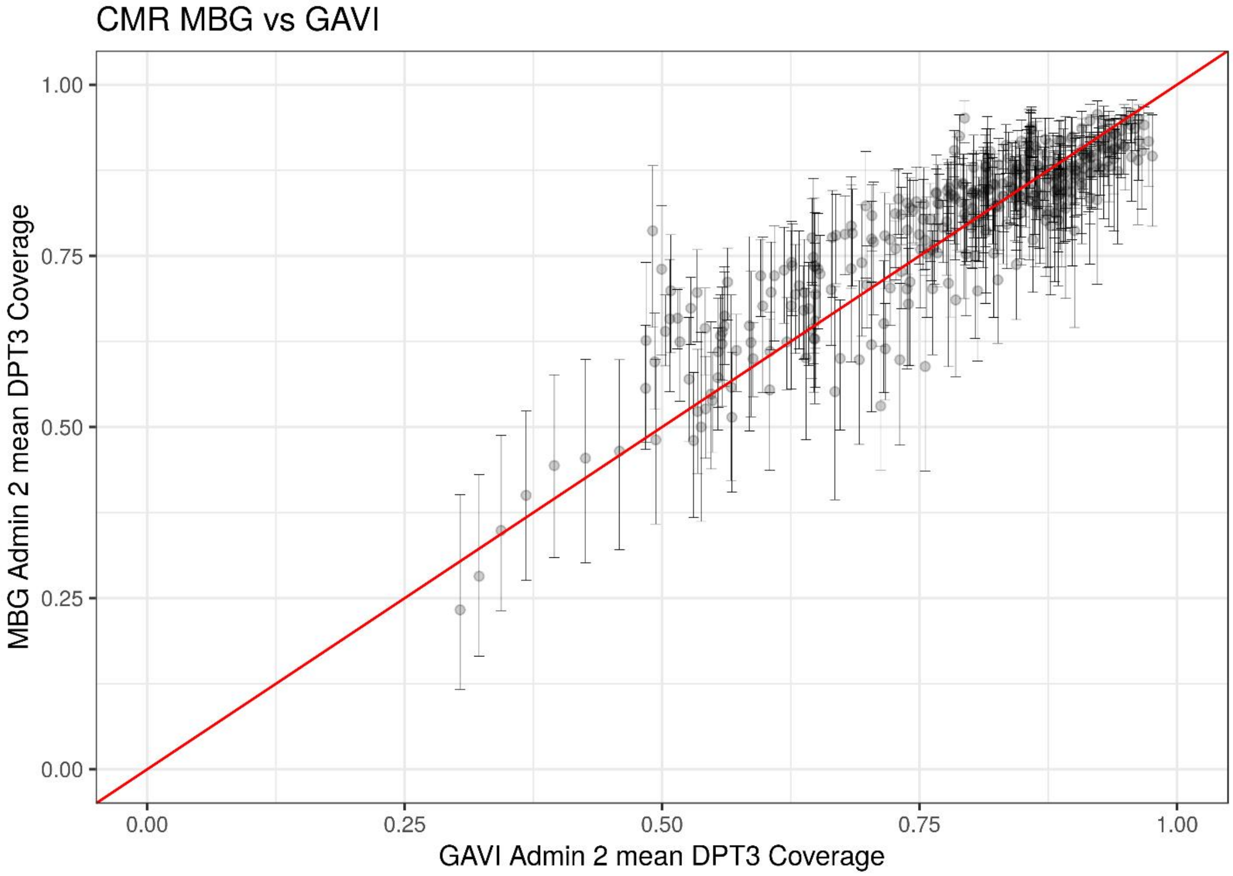

1106 **Supplementary Figure 40: Comparison of MBG and Gavi FCE second-administrative-level estimates**
1107 **of DPT3 coverage for Mozambique, 2000-2015.**
1108

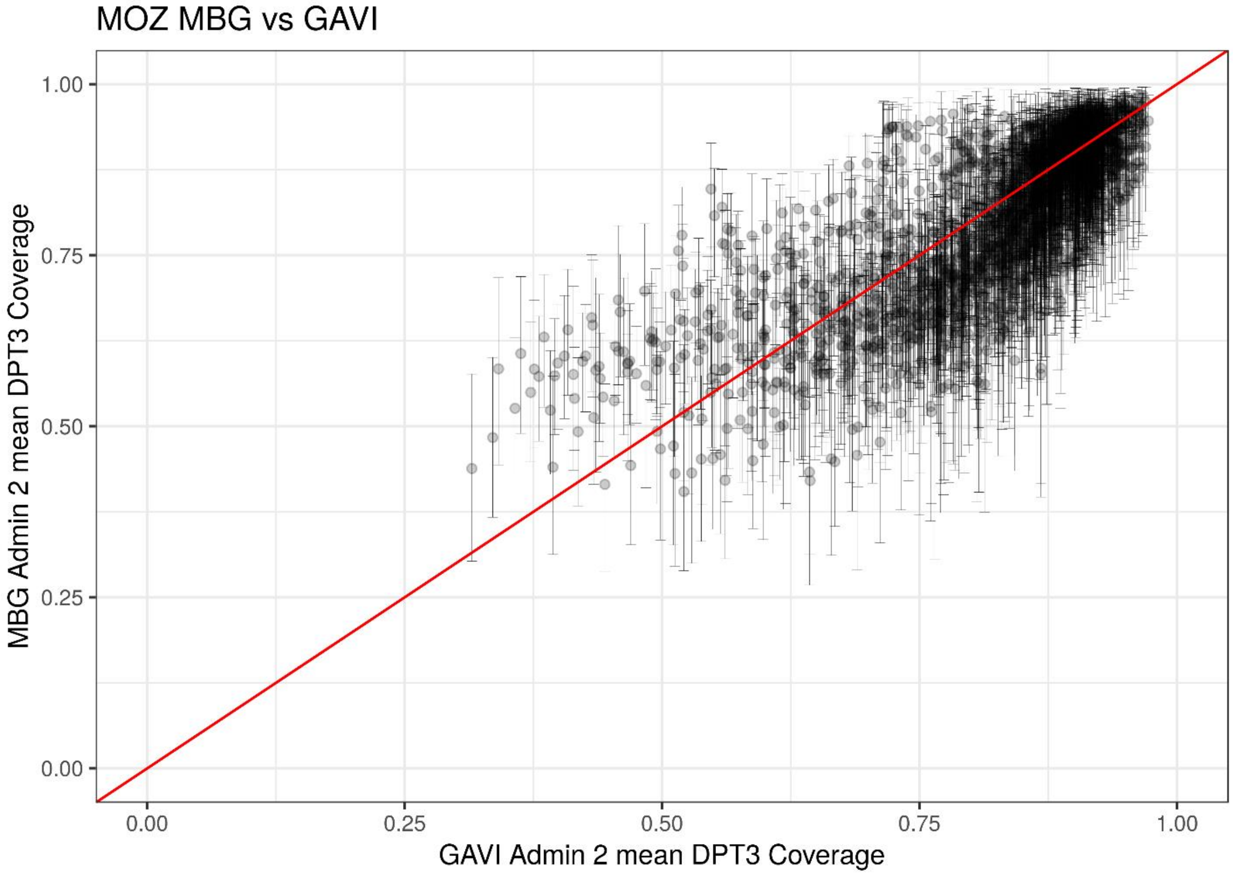

1109
1110

Supplementary Figure 41: Comparison of MBG and Gavi FCE second-administrative-level estimates of DPT3 coverage for Chad, 2000-2015.

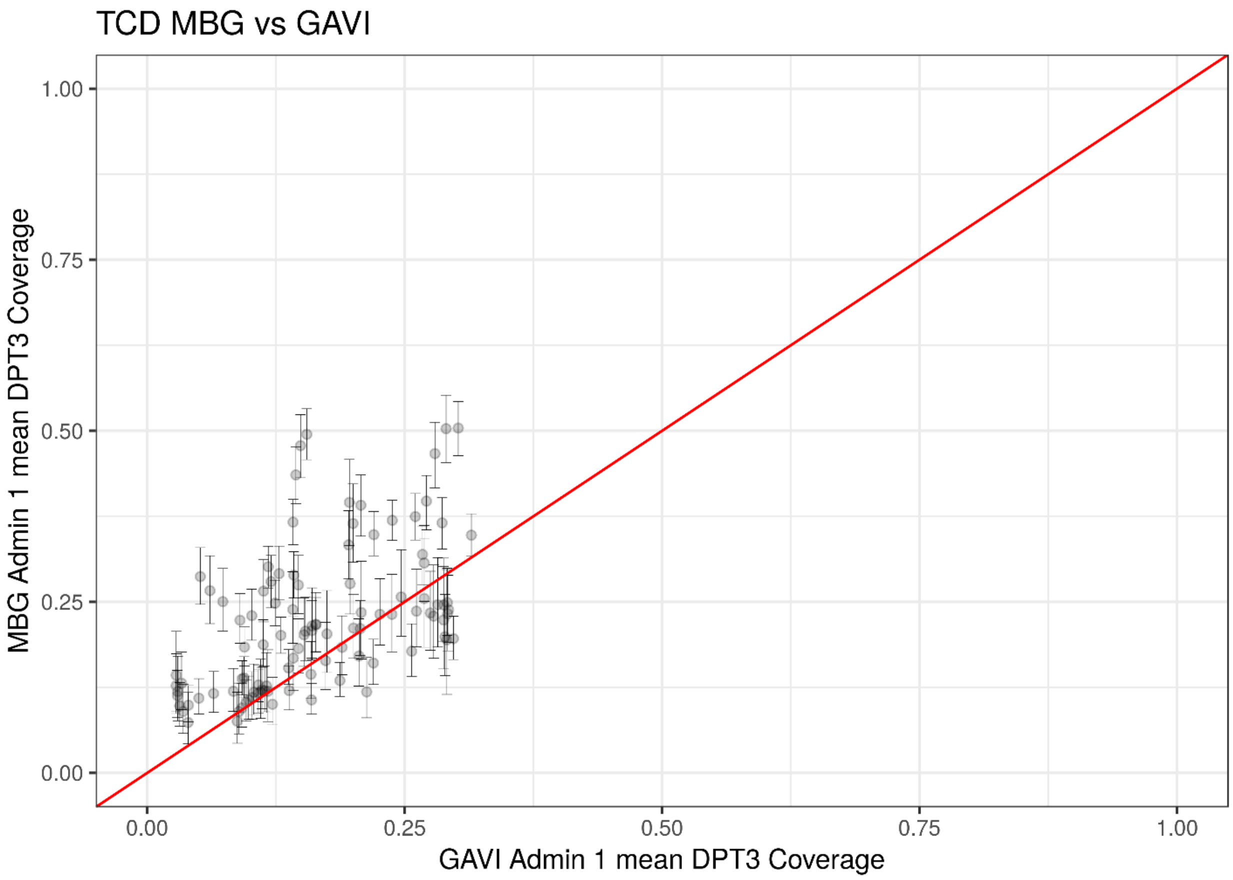

Supplementary Figure 42: Comparison of MBG and Gavi FCE second-administrative-level estimates of DPT3 coverage for Uganda, 2000-2015.

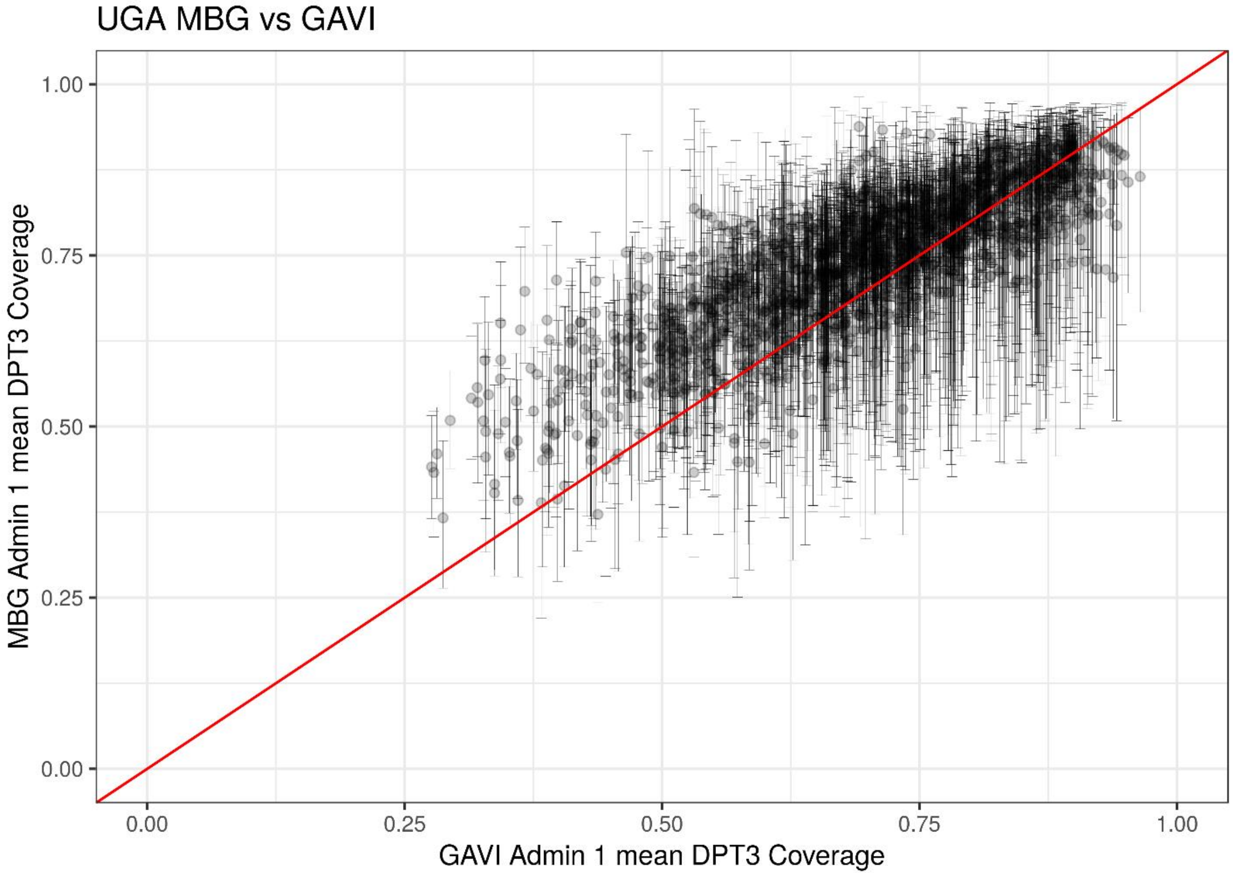

Supplementary Figure 43: Comparison of MBG and Gavi FCE second-administrative-level estimates of DPT3 coverage for Zambia, 2000-2015.

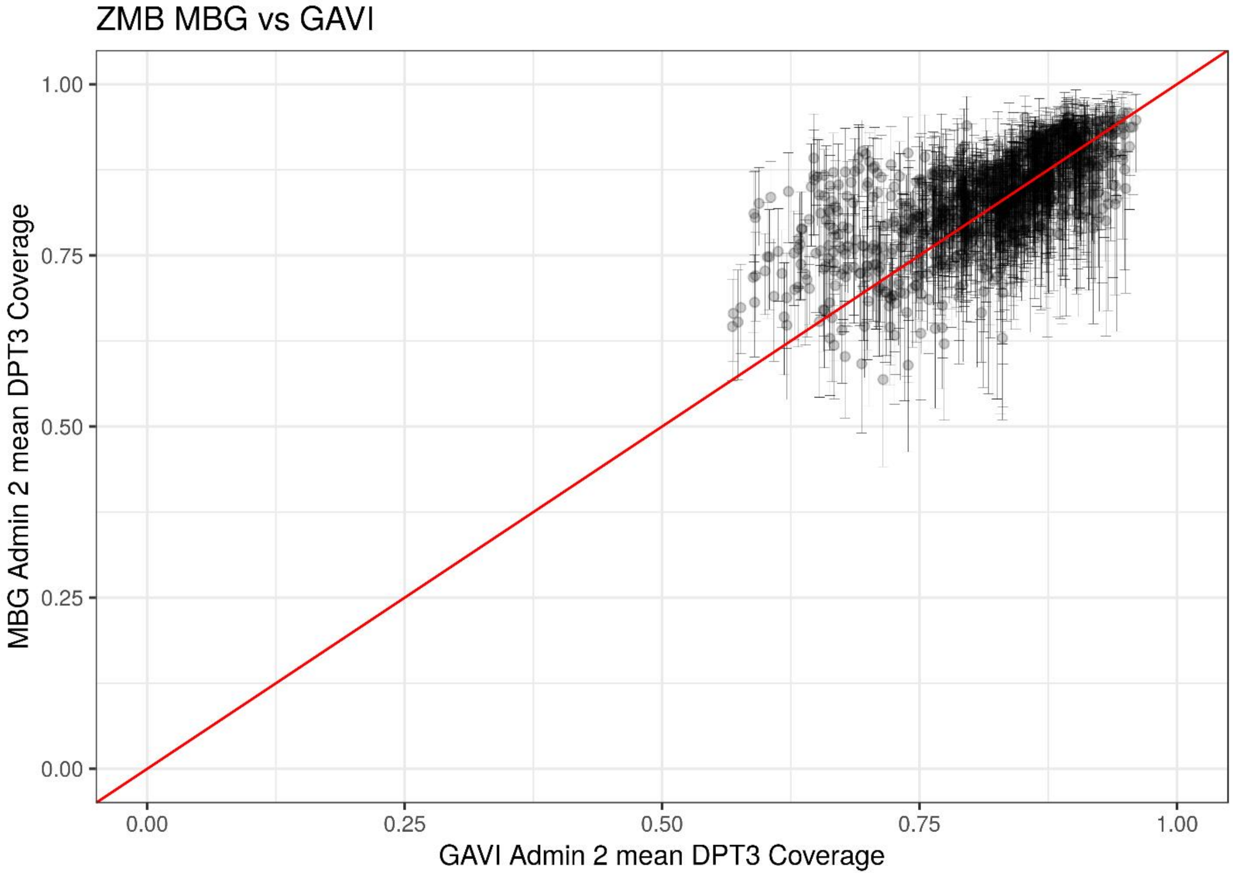

6.0 References

1. Tatem AJ. WorldPop, open data for spatial demography. *Scientific Data* 2017; 4: 170004.

2. S Channan, K. Collins, W. R. Emanuel. Global mosaics of the standard MODIS land cover type data. 2014. http://glcf.umd.edu/data/lc/ (accessed Mar 15, 2019).

3. GeoNetwork. Global Administrative Unit Layers (GAUL). 2007. http://www.fao.org/geonetwork/srv/en/metadata.show?id=12691 (accessed Mar 15, 2019).

4. Global Administrative Areas. GADM Database of Global Administrative Areas. 2018. http://gadm.org/data.html (accessed Mar 15, 2019).

5. Osgood-Zimmerman A, Millear AI, Stubbs RW, Shields C, Pickering BV, Earl L, et al. Mapping child growth failure in Africa between 2000 and 2015. *Nature* 2018; 555(7694): 41–7.

6. Graetz N, Friedman J, Osgood-Zimmerman A, Burstein R, Biehl MH, Shields C, et al. Mapping local variation in educational attainment across Africa. *Nature* 2018; 555(7694): 48–53.

7. Golding N, Burstein R, Longbottom J, Browne AJ, Fullman N, Osgood-Zimmerman A, et al. Mapping under-5 and neonatal mortality in Africa, 2000–15: a baseline analysis for the Sustainable Development Goals. *Lancet* 2017; 390(10108): 2171–82.

8. Lumley, T. Analysis of Complex Survey Samples. *J Stat Softw* 2004; 9(8): 1-19.

9. Lloyd CT, Sorichetta A, Tatem AJ. High resolution global gridded data for use in population studies. *Sci Data* 2017; 4: 170001.

10. WorldPop. WorldPop dataset. 2019. http://www.worldpop.org.uk/data/get_data/ (accessed 15 Mar 2019).

11. Naghavi M, Abajobir AA, Abbafati C, Abbas KM, Abd-Allah F, Abera SF, et al. Global, regional, and national age-sex specific mortality for 264 causes of death, 1980–2016: a systematic analysis for the Global Burden of Disease Study 2016. *Lancet* 2017; 390(10100): 1151–210.

12. Bhatt S, Cameron E, Flaxman SR, Weiss DJ, Smith DL, Gething PW. Improved prediction accuracy for disease risk mapping using Gaussian process stacked generalization. *J R Soc Interface* 2017; 14(134): 20170520.

13. Stein ML. Interpolation of Spatial Data: Some Theory for Kriging. New York: Springer-Verlag; 1999.

14. Rue H, Martino S, Chopin N. Approximate Bayesian inference for latent Gaussian models by using integrated nested Laplace approximations. *J R Stat Soc Ser B Stat Methodol*. 2009; 71(2): 319–92.

15. Martins TG, Simpson D, Lindgren F, Rue H avard. Bayesian computing with INLA: new features. *Comput Stat Data Anal.* 2013; 67: 68–83.

17. Lindgren F, Rue H, Lindström J. An explicit link between Gaussian fields and Gaussian Markov random fields: the stochastic partial differential equation approach. *J R Stat Soc Ser B Stat Methodol.* 2011; 73(4): 423–98.

18. Waller, L. & Carlin, B. Disease mapping. In: Handbook of Spatial Statistics. CRC Press; 2010. p. 217–43.

19. Hosmer DW, Lemeshow S, Sturdivant RX. Ordinal Logistic Regression Models. In: Applied logistic regression. Third edition. Hoboken, New Jersey: Wiley; 2013. p. 289–305.

20. Coffey MP, Feingold M, Bromberg J. A normed measures of variability among proportions. *Comput Stat Data Anal.* 1988; 7(2): 127–141.

21. Roberts DR, Bahn V, Ciuti S, Boyce MS, Elith J, Guillera-Arroita G, et al. Cross-validation strategies for data with temporal, spatial, hierarchical, or phylogenetic structure. *Ecography* 2017; 40(8): 913–29.

22. World Health Organization (WHO). WHO/UNICEF Joint Reporting Process. 2013. http://www.who.int/immunization/monitoring_surveillance/routine/reporting/reporting/en/ (accessed 14 Mar 2019).

23. Murray CJL, Shengelia B, Gupta N, Moussavi S, Tandon A, Thieren M. Validity of reported vaccination coverage in 45 countries. *Lancet* 2003; 362(9389): 1022–7.

24. Haddad S, Bicaba A, Feletto M, Fournier P, Zunzunegui MV. Heterogeneity in the validity of administrative-based estimates of immunization coverage across health districts in Burkina Faso: implications for measurement, monitoring and planning. *Health Policy Plan.* 2010; 25(5): 393–405.

25. Ng M, Freeman MK, Fleming TD, et al. Smoking prevalence and cigarette consumption in 187 countries, 1980-2012. *JAMA* 2014; 311(2): 183–92.

26. Ng M, Fleming T, Robinson M, Thomson B, Graetz N, Margono C, et al. Global, regional, and national prevalence of overweight and obesity in children and adults during 1980–2013: a systematic analysis for the Global Burden of Disease Study 2013. *Lancet* 2014; 384(9945): 766–81.

27. Wang H, Liddell CA, Coates MM, Mooney MD, Levitz CE, Schumacher AE, et al. Global, regional, and national levels of neonatal, infant, and under-5 mortality during 1990–2013: a systematic analysis for the Global Burden of Disease Study 2013. *Lancet* 2014; 384(9947): 957–79.

28. Cross-Country: Findings from the 2015 Gavi Full Country Evaluations. 2016. http://www.gavi.org/results/evaluations/full-country-evaluations/ (accessed 16 Mar 2019).

1196
